# Ammonia Oxidation with a Ruthenium Polypyridyl Complex: Mechanism-Guided Approach to Low Overpotential Electrocatalysis

**DOI:** 10.1021/jacsau.6c00733

**Published:** 2026-07-11

**Authors:** Maximilian Seiß, Elisa Dickgießer, Sebastian Dechert, Franc Meyer

**Affiliations:** † 9375 University of Göttingen, Institute of Inorganic Chemistry, Tammannstr. 4, 37077 Göttingen, Germany; ‡ University of Göttingen, International Center for Advanced Studies of Energy Conversion (ICASEC), Tammannstr. 6, 37077 Göttingen , Germany

**Keywords:** ammonia oxidation, electrocatalysis, homogeneous catalysis, ruthenium, mechanistic studies, PCET

## Abstract

The use of ammonia as a potential zero-carbon fuel has attracted high interest in the quest for novel sustainable energy carriers. This requires a deep understanding of the principles of the ammonia oxidation reaction (AOR) to guide the development of efficient catalysts. Here, we report the complex [Ru^II^(TPA)­(NH_3_)_2_]­(PF_6_)_2_ (**1­(PF**_**6**_ **)**_**2**_; TPA = tris­(2-pyridylmethyl)­amine), and we demonstrate that its 1e^–^ oxidation in MeCN is partially ligand centered and finally produces [Ru^II^(TPA)­(MeCN)­(NH_3_)]­(PF_6_)_2_ (**2­(PF**_**6**_ **)**_**2**_). Deprotonation of **1** ^**3+**^ (p*K*_A_ = 12.6) is derived as the rate-determining step, and bimolecular N–N coupling of the key intermediate [Ru­(TPA)­(NH_3_)­(NH_2_)]^2+^ (**4** ^**2+**^) is proposed based on ^15^N labeling experiments and electrochemical studies. **1­(PF**_**6**_ **)**_**2**_ is then investigated as a homogeneous AOR electrocatalyst, and the formation of N_2_ and H_2_ is confirmed during electrolysis at a moderate operating potential. DFT computations established a complete PCET map to evaluate potential reaction pathways and, in combination with spectroscopic and electrochemical studies as well as product analysis using ^15^N labeling, allowed us to propose a catalytic cycle where N–N bond formation occurs via intermolecular coupling (*I2M*) at an early amido stage (**4** ^**2+**^) of the PCET sequence. Circumventing higher metal oxidation states gives rise to a relatively low AOR overpotential of 0.96 V for **1** ^**2+**^. Complex **2** ^**2+**^ is identified as an important intermediate that is catalytically competent and initiates a second cycle, while [Ru^II^(TPA)­(MeCN)_2_]^2+^ (**XI** ^**2+**^) represents an off-cycle product leading to gradual loss of catalytic activity.

## Graphical abstract

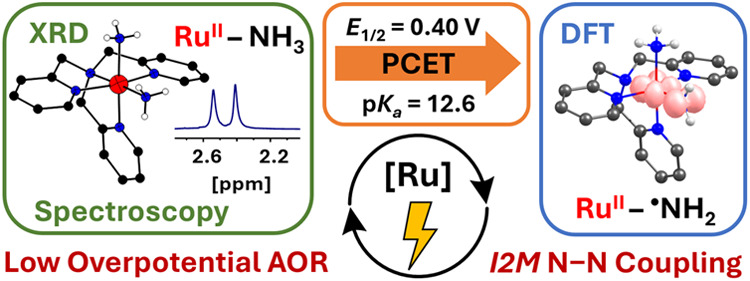

## Introduction

The global climate crisis caused by enormous emissions of greenhouse gases from fossil fuel consumption is a major threat to human civilization on Earth.^1^ While implementation of established renewable energy sources plays a key role en route to a sustainable energy economy, the rapid growth of novel energy consumers, such as AI data centers, raises the question of whether existing technologies can keep pace with global energy demand.^2^ In recent years, ammonia has attracted attention as a potential zero-carbon fuel, being particularly interesting due to its existing global supply chain, high energy density, and safety in large-scale handling.^3–5^ While engineers envision ammonia as a substrate for fuel cells^6–9^ or hydrogen-producing electrolyzers,^10,11^ the development of efficient catalysts for the ammonia oxidation reaction (AOR) remains challenging. The design and investigation of homogeneous catalysts for the oxidation of ammonia to dinitrogen offers the opportunity to understand mechanistic pathways and principles of the complex 6e^–^/6H^+^ transfer process, thus paving the way for rational catalyst design and improvement.^12–14^ A number of homogeneous AOR electrocatalysts based on first-row transition metals such as Mn,^15^ Fe,^16–20^ and Cu^21–23^ have been reported, yet the majority of catalysts rely on Ru as the active metal site. Figure 1 summarizes noteworthy examples for mononuclear ruthenium complexes applied as AOR electrocatalysts, including pioneering works by Smith and co-workers (**I** ^**2+**^)^24^ and Nishibayashi and co-workers (**II)**,^25^ as well as more recent studies by Llobet and co-workers (**III**),^26^ Liu and co-workers (**IV** ^**2+**^),^27^ Lau and co-workers (**V** ^**+**^),^28^ Zhang and co-workers (**VI**, **VIII**),^29,30^ and Li and co-workers (**VII**).^31^ Furthermore, we have recently reported the first example of a diruthenium AOR electrocatalyst featuring a highly preorganized dinuclear substrate binding site.^32^ Notably, all those catalysts, except for **V** ^**+**^, include polypyridyl ligand frameworks, which are popular in the field of oxidation electrocatalysis due to their oxidative ruggedness,^33^ and except for **V** ^**+**^ and **VII**, all systems displayed in Figure 1 contain a rigid bipyridine or terpyridine ligand backbone.

**Figure 1:**
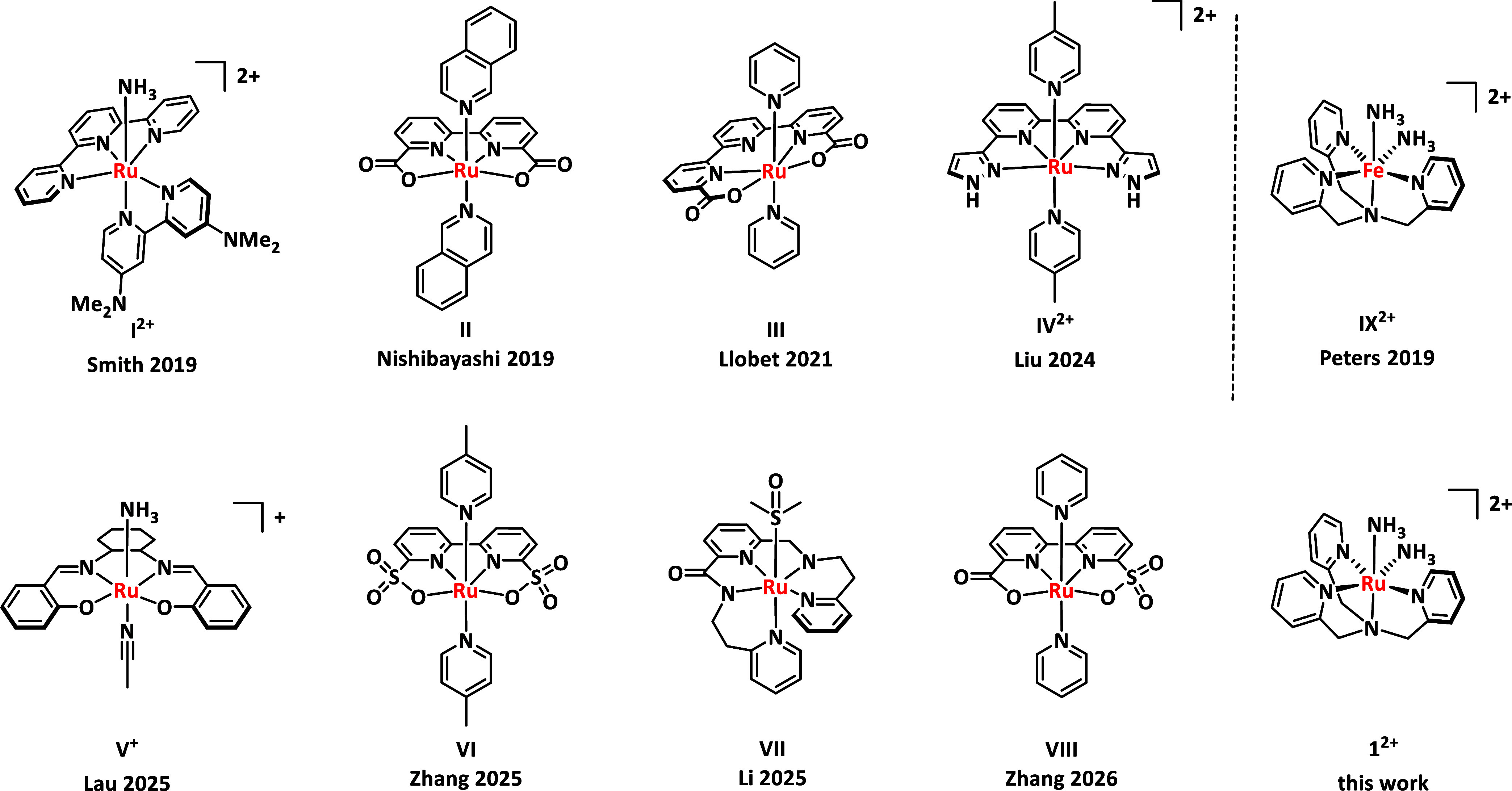
Selected mononuclear ruthenium complexes reported in the literature that mediate the AOR and the TPA-based iron AOR electrocatalyst reported by Peters and co-workers.

Different mechanistic scenarios have been proposed for the variety of reported catalysts. Two key pathways were discussed in great detail for the conceptually similar, but much more thoroughly studied metal-catalyzed water oxidation reaction (WOR): either water as a nucleophilic substrate attacks at a high-valent metal-oxo species (*WNA*) or two metal-oxo units couple intermolecularly (*I2M*), both scenarios eventually leading to the formation of an O–O bond.^34^ Analogous mechanisms can be operative for the AOR as summarized in Scheme 1, involving sequential proton-coupled oxidation of the ammonia complex to the respective amido, imido, and nitrido species. While nucleophilic attack of ammonia (*ANA*) is generally expected to occur no earlier than at the imido stage for orbital reasons,^12^ bimolecular coupling (*I2M*) may be encountered at any stage (amido, imido, and nitrido), thus not being limited to high-valent intermediates as is the case for the WOR. In fact, both mechanistic pathways have been observed in the context of electrocatalytic AOR. Complexes **III** and **VI**–**VIII** were proposed to proceed via the *ANA* mechanism,^26,29–31^ while complex **II** is assumed to undergo *I2M*.^25^ Complexes **IV** ^**2+**^ and **V** ^**+**^ can follow both pathways (depending on conditions and transitioning from chemical to electrocatalysis).^27,28^ An *ANA* scenario was originally also proposed for **I** ^**2+**^,^24^ but a recent detailed mechanistic study using voltammetry coupled electrospray ionization mass spectrometry (VESI-MS) identified key intermediates during electrocatalytic AOR with **I** ^**2+**^ and revealed that the catalytic cycle may follow multiple reaction trajectories depending on ammonia and local proton concentrations as well as applied potential, where the *I2M* and *ANA* pathways (from {Ru^III^(NH_2_)} and {Ru^IV^(NH)} intermediates, respectively) compete as N–N bond-forming steps.^35^ From those studies, it can be concluded that the delocalization of the unpaired electron from Ru to N in the amido species {Ru^III^(NH_2_)} will favor *I2M* already after the first PCET step.

**Scheme 1:**
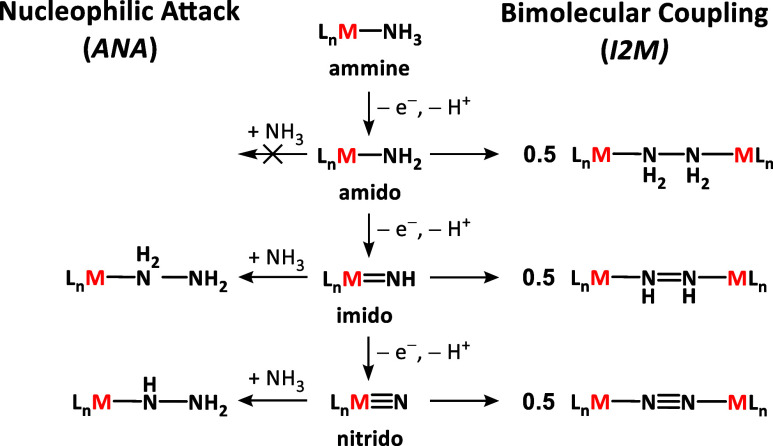
Mechanistic Pathways for the Metal-Catalyzed Ammonia Oxidation Reaction (AOR): Ammonia Nucleophilic Attack (ANA, Left) and Bimolecular Coupling (I2M, Right)

While the number of reported homogeneous AOR electrocatalysts is continuously growing, there is no systematic study on the influence of different metals in comparable ligand environments on catalyst performance and mechanism. In 2019, Peters and co-workers developed an early example of a Fe-based AOR catalyst by employing the tris­(2-pyridylmethyl)­amine (TPA) ligand framework (**IX** ^**2+**^, Scheme 1).^16^ This catalyst operates with a remarkably fast rate (*k*_obs_ = 3.7 × 10^7^ M^–1^s^–1^) but at a substantially high onset potential of 0.7 V vs Fc^+/0^ (i.e, at high overpotential), and it was assumed that N–N bond formation occurs after generation of some Fe^IV^ species. Later, the same group reported an improved system featuring a more rigid, stronger field ligand environment by replacing the sp^3^ nitrogen and one pyridine of TPA with a bipyridine group. The resulting iron complex is an active AOR electrocatalyst with increased stability, 50 times faster AOR rate, and an onset potential lowered by 0.25 V compared to the TPA system **IX** ^**2+**^.^20^ In this work, we employ the TPA ligand platform to synthesize a related ruthenium complex **1­(PF**_**6**_ **)**_**2**_, explore its performance as an AOR electrocatalyst, and gain insights into potential mechanistic scenarios by a combined experimental and computational approach. This work aims at elucidating the distinct AOR pathways as well as thermodynamic and kinetic foundations for Ru vs Fe catalysts in the same ligand settings, with a particular focus on a mechanism-based lowering of overpotentials.

## Results and Discussion

### Synthesis of a Ruthenium­(II) Tris­(2-pyridylmethyl)­amine Diammine Complex and Its Oxidation Chemistry

The reaction of [RuCl_2_(*p*-cymene)]_2_ (*p*-cymene = 1-methyl-4- isopropylbenzene)^36^ with TPA in MeCN affords complexes [Ru^II^(TPA)­L_2_]­Cl_*x*_ (**X**, L = MeCN, Cl^–^) with MeCN and chloride ligands being in solution equilibrium, as earlier reported by Britovsek and co-workers.^37^ Complex mixture **X** was dissolved in a concentrated aqueous NH_3_ solution and heated to 100 °C overnight in a closed reaction vessel. Upon addition of NH_4_PF_6_, the novel complex [Ru^II^(TPA)­(NH_3_)_2_]­(PF_6_)_2_ (**1­(PF**_**6**_ **)**_**2**_, Scheme 2) was isolated as a yellow precipitate.

**Scheme 2:**
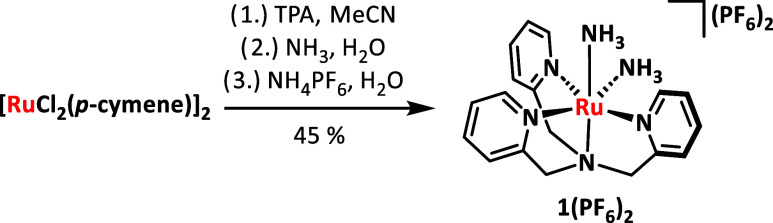
Synthetic Route for the Preparation of Diammine Complex **1­(PF**_**6**_ **)**_**2**_. Reaction Conditions: (1) Reflux, 12 h; (2) Reflux, 12 h; (3) rt, 10 min

Compound **1­(PF**_**6**_ **)**_**2**_ was fully characterized using NMR and UV/vis spectroscopy, ESI-MS, and Elemental Analysis (see SI, section 2.1). The ^1^H NMR spectrum of complex **1­(PF**_**6**_ **)**_**2**_ shows two broad singlets at 2.54 and 2.41 ppm (Figure 2, top) assigned to the two ammine ligands based on the corresponding correlations in a ^1^H–^15^N HSQC NMR experiment (see Figure S9). Yellow block-shaped crystals suitable for X-ray diffraction analysis were grown by slow diffusion of Et_2_O into a solution of **1­(PF**_**6**_ **)**_**2**_ in MeCN at 5 °C. The molecular structure of the cation **1** ^**2+**^ is shown in Figure 3 (top left). It confirms the presence of two chemically inequivalent ammonia molecules coordinated to the ruthenium center in accordance with the NMR spectroscopic findings. Despite the different *trans* donors, the two Ru–NH_3_ bond lengths are essentially identical (2.1599(16) and 2.1547(16) Å), but they are significantly longer than the Ru–N bonds involving the TPA ligand (2.057–2.074 Å; see Figure S34). When reacted with 1.0 equiv. of the tris­(4-bromophenyl)­ammoniumyl radical (*Magic Blue*) as a strong oxidizing agent, complex **1­(PF**_**6**_ **)**_**2**_ unexpectedly does not undergo metal-centered 1e^–^ oxidation to the corresponding Ru^III^ species but instead is transformed into [Ru^II^(TPA)­(MeCN)­(NH_3_)]­(PF_6_)_2_ (**2­(PF**_**6**_ **)**_**2**_, Scheme 3). Compound **2­(PF**_**6**_ **)**_**2**_ was characterized by NMR and UV–vis spectroscopy as well as ESI-MS (see SI, section 2.3). The ^1^H NMR spectrum (Figure 2, middle) shows a singlet at 2.42 ppm assigned to the coordinated ammonia molecule (supported by the corresponding correlation in the ^1^H–^15^N HSQC NMR spectrum; see Figure S26) as well as a singlet at 2.70 ppm assigned to coordinated MeCN. It thus indicates that one of the two ammine ligands of **1­(PF**_**6**_ **)**_**2**_ is selectively replaced by MeCN. Yellow block-shaped crystals suitable for X-ray analysis were grown by slow diffusion of Et_2_O into a solution of **2­(PF**_**6**_ **)**_**2**_ in MeCN at 5 °C. The molecular structure of the cation **2** ^**2+**^ is shown in Figure 3 (top right) and reveals that the ammine ligand *cis* to the bridgehead sp^3^ N atom of TPA is preserved during the reaction. Its Ru–NH_3_ bond (2.142(3) Å) is slightly shortened compared to **1** ^**2+**^ (2.1547(16) Å) while the Ru–N^NCMe^ bond is the shortest of all Ru–N bonds (2.039(3) Å; Figure S35), as expected.

**Figure 2:**
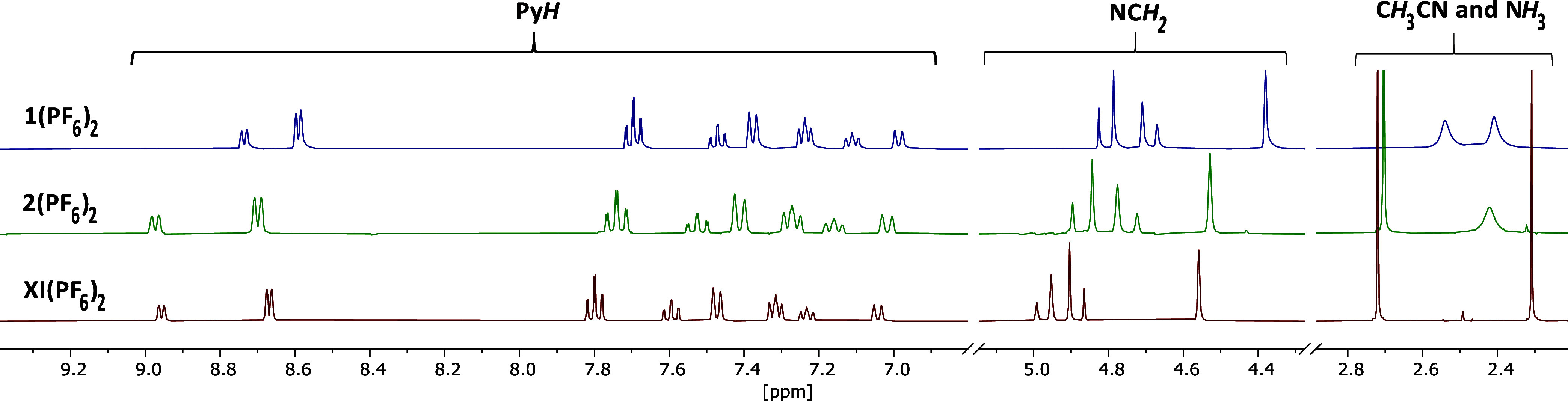
^1^H NMR spectra of compounds **1­(PF**_**6**_ **)**_**2**_ (top), **2­(PF**_**6**_ **)**_**2**_ (middle), and **XI­(PF**_**6**_ **)**_**2**_ (bottom) in MeCN-*d*_3_.

**Figure 3:**
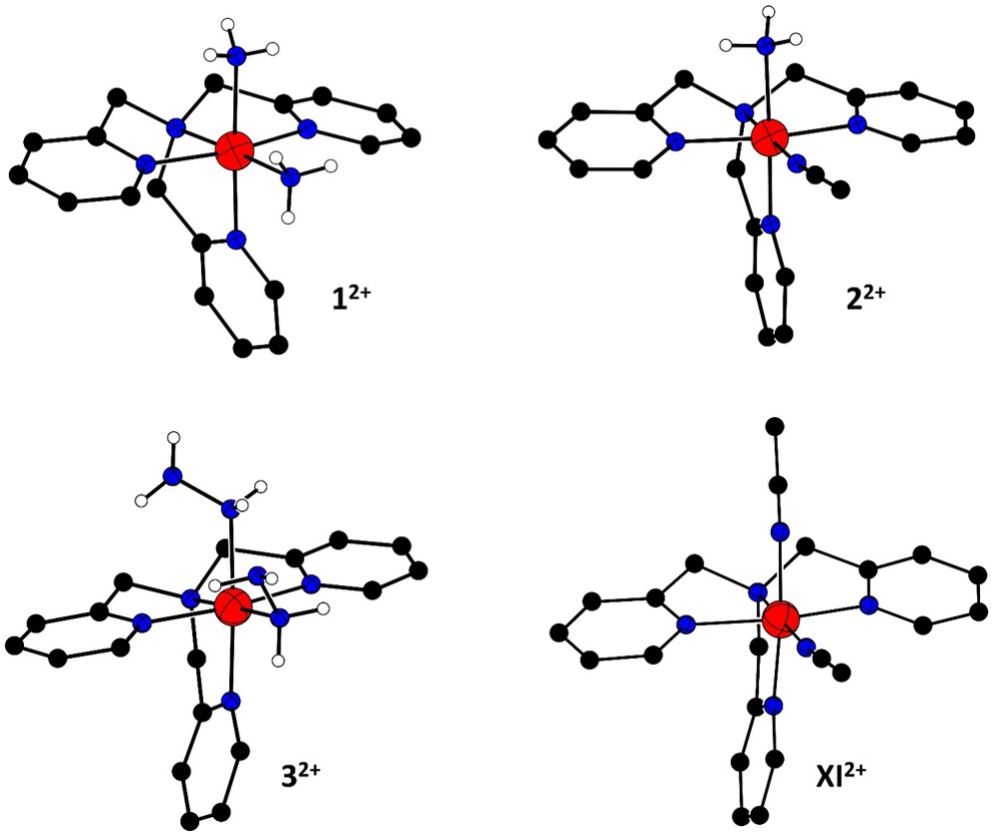
Molecular structures of the cations of **1­(PF**_**6**_ **)**_**2**_, **2­(PF**_**6**_ **)**_**2**_, **3­(BPh**_**4**_ **)**_**2**_, and **XI­(PF**_**6**_ **)**_**2**_ ^37^ determined by X-ray crystallography (most hydrogen atoms and cocrystallized solvent molecules are omitted for clarity). Color code: Ru, red; C, black; N, blue; H, white.

**Scheme 3:**
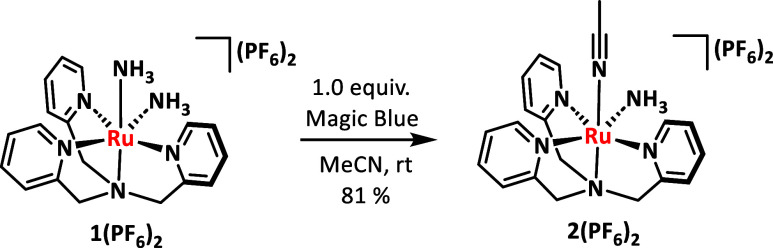
Oxidation of **1­(PF**_**6**_ **)**_**2**_ to Give Monoacetonitrile Complex **2­(PF**_**6**_ **)**_**2**_

When reacting **2­(PF**_**6**_ **)**_**2**_ with another 1.0 equiv. of *Magic Blue* in MeCN at rt, formation of the known^37^ bis­(acetonitrile) complex [Ru^II^(TPA)­(MeCN)_2_]­(PF_6_)_2_ (**XI­(PF**_**6**_ **)**_**2**_) is observed. This indicates another oxidation event leading to the replacement of the second ammine ligand instead of a metal-centered oxidation to a Ru^III^ species. For comparative reasons, **XI­(PF**_**6**_ **)**_**2**_ has also been synthesized independently following a modification of the procedure reported by Britovsek and co-workers,^37^ and it was characterized using ^1^H NMR and UV–vis spectroscopy (see SI, section 2.5). The ^1^H NMR spectrum shows two distinct singlets at 2.72 and 2.31 ppm assigned to the methyl groups of the two coordinated acetonitrile ligands (Figure 2, bottom). The molecular structure of the cation **XI** ^**2+**^ as determined by X-ray crystallography by Britovsek and co-workers^37^ is included in Figure 3 for comparison (bottom, right).

### Mechanistic Scenario for the Oxidation of 1­(PF_6_)_2_

The formation of Ru^II^ complex **2­(PF**_**6**_ **)**_**2**_ upon 1e^–^ oxidation of Ru^II^ complex **1­(PF**_**6**_ **)**_**2**_ indicates that the oxidation event is taking place in the ligand sphere of the complex. We propose a metal-mediated oxidation of the coordinated ammonia as a plausible mechanistic scenario, as elaborated in Scheme 4. Here, 1e^–^ oxidation of **1** ^**2+**^ is coupled to a deprotonation event yielding the Ru-amido complex **4** ^**2+**^, which can be described as a resonance hybrid of a Ru^III^/amido anion and a Ru^II^/amido radical species. This is reminiscent of the situation in our previously reported diruthenium AOR catalyst, for which it was shown that such formal Ru^III^–NH_2_ species can exhibit significant N-centered radical character, leading to a facilitated N–N bond formation with a low energy barrier.^32^ Delocalization of the unpaired electron over the Ru ion and NH_2_ ligand has also been proposed for the amido intermediate resulting from 1e^–^/1H^+^ oxidation of **I** ^**2+**^.^35^ Bimolecular radical coupling of **4** ^**2+**^ then affords compound [Ru­(TPA)­(NH_3_)]_2_(μ-N_2_H_4_) (**5** ^**4+**^), which can further react via hydrazine replacement or disproportionation to give the experimentally observed product **2** ^**2+**^ after coordination of MeCN from solvent. The ultimate release of a base (hydrazine or the disproportionation product ammonia, respectively) favors the deprotonation event upon oxidation, contributing to the driving force. The same mechanism involving the second ammine ligand is proposed for the further oxidation of **2** ^**2+**^ forming **XI** ^**2+**^.

**Scheme 4:**
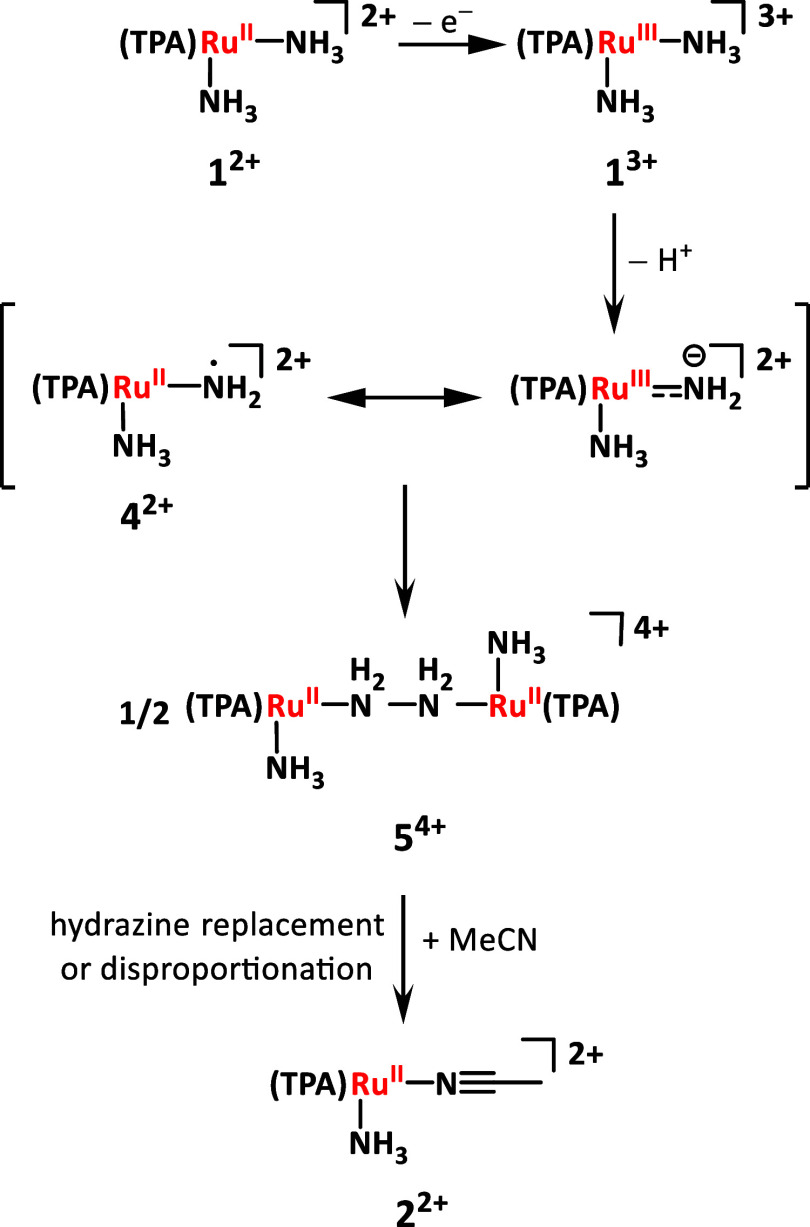
Proposed Mechanistic Scenario for the Oxidation of **1** ^**2+**^ Leading to the Formation of **2** ^**2+**^

EPR experiments (see SI, Section 5) were conducted to detect intermediate radical species formed upon oxidation of **1­(PF**_**6**_ **)**_**2**_ with *Magic Blue*. To that end, a solution of **1­(PF**_**6**_ **)**_**2**_ (10 mM) in MeCN was placed in an EPR tube under N_2_ atmosphere, and 0.5 equiv of *Magic Blue* in MeCN was added. After rapid mixing, the sample was immediately (within a few seconds) frozen by immersion of the tube into liquid N_2_. An intense EPR signal, which does not correspond to *Magic Blue*, was observed for the resulting frozen solution at 120 K. While detailed assignment of the spectrum is challenging (see SI), it clearly indicates the presence of one or multiple transient paramagnetic species (likely **1** ^**3+**^ and **4** ^**2+**^) upon oxidation of **1** ^**2+**^, as suggested by the mechanism depicted in Scheme 4. When repeating the experiment but keeping the sample at room temperature for 10 min after the addition of *Magic Blue*, the EPR signal almost vanished, in accordance with the proposed coupling to give **5** ^**4+**^ and formation of the final product **2** ^**2+**^.

To probe whether simple replacement of the bridging hydrazine ligand from **5** ^**4+**^ occurs, the mixture obtained after reacting **1** ^**2+**^ with *Magic Blue* was analyzed photometrically using the hydrazine determination method developed by Watt and Chrisp (based on *p*-dimethylaminobenzaldehyde as reagent).^38^ No characteristic absorption at 458 nm could be observed, indicating the absence of free hydrazine. Furthermore, we performed the reaction of **1** ^**2+**^ with *Magic Blue* in the presence of an excess of phenyl isocyanate, which is stable under the reaction conditions and reacts with hydrazine readily and fast to phenyl semicarbazide.^39,40^ However, no phenyl semicarbazide could be observed by means of ^1^H NMR spectroscopy, in agreement with the result of the photometric test. The alternative reaction pathway mentioned in Scheme 4 is the metal-induced disproportionation of the coordinated hydrazine. Despite being a rather unusual reactivity, it has previously been proposed^41^ and demonstrated^42^ for transition metal complexes. The expected ultimate disproportionation products of hydrazine are N_2_ and NH_3_ (eq 1).

1 $$3\;N_{2}H_{4}\to 4\;{\mathrm{NH}}_{3}+N_{2}$$

According to the mechanism shown in Scheme 4, 1.0 equiv of **1** ^**2+**^ forms 0.5 equiv of hydrazine upon 1e^–^ oxidation, resulting in the formation of 0.67 equiv of NH_3_ (eq 1). Since also 1.0 equiv of protons is released, the formed NH_3_, being the strongest available base, will be present in its protonated form (NH_4_^+^). When performing the reaction of **1­(PF**_**6**_ **)**_**2**_ with 1.0 equiv. of *Magic Blue* in MeCN-*d*_3_ and measuring a ^1^H NMR spectrum of the reaction mixture, a 1:1:1 triplet at 5.9 ppm, indicative of NH_4_^+^, can indeed be observed (Figure 4). Integration of this signal relative to the product signals of **2** ^**2+**^ reveals the formation of 0.84 equiv of NH_4_^+^ and thus slightly more than ideally expected.

**Figure 4:**
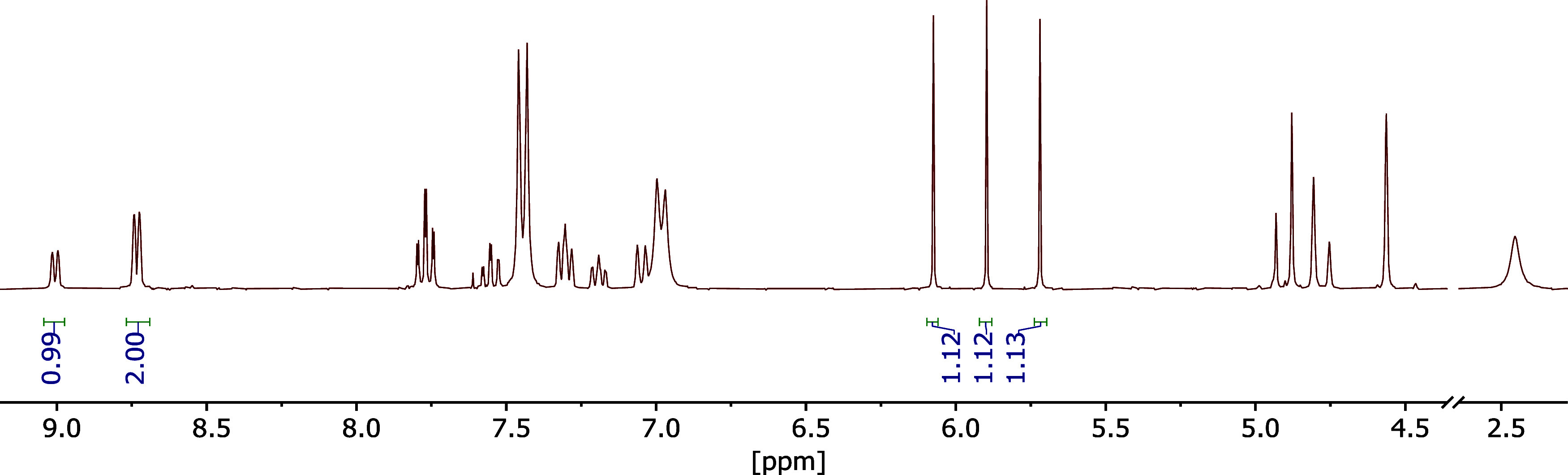
^1^H NMR spectrum of the reaction mixture after treatment of **1­(PF**_**6**_ **)**_**2**_ with 1.0 equiv. of *Magic Blue* in MeCN-*d*_3_.

In order to prove the formation of N_2_ as the second disproportionation product, we have prepared the isotopically labeled compound [Ru­(TPA)­(^15^NH_3_)_2_]­(PF_6_)_2_ (^**15N**^ **1­(PF**_**6**_ **)**_**2**_). This allowed to test for gaseous reaction products using a mass spectrometric gas analyzer. For this purpose, we performed the reaction of **1­(PF**_**6**_ **)**_**2**_ with 1.0 equiv of *Magic Blue* in MeCN three times: (a) only with ^**14N**^ **1­(PF**_**6**_ **)**_**2**_, (b) with a 1:1 mixture of ^**14N**^ **1­(PF**_**6**_ **)**_**2**_ and ^**15N**^ **1­(PF**_**6**_ **)**_**2**_, and (c) only with ^**15N**^ **1­(PF**_**6**_ **)**_**2**_. After completion of the reaction, which is indicated by the disappearance of the blue color, the headspace of the reaction vessel was opened to the mass spectrometric gas analyzer (*t* = 0). Figure 5 shows the ion currents for *m*/*z* = 29 and *m*/*z* = 30 that are assigned to ^14,15^N_2_ and ^15,15^N_2_, respectively, for the three experiments. After an initial spike of ion current due to a pressure change after exposing the analyzer to the reaction vessel’s headspace, the signal evolution was monitored over several minutes. When using only ^**14N**^ **1­(PF**_**6**_ **)**_**2**_ (Figure 5a), no ^14,15^N_2_ or ^15,15^N_2_ can be observed. In the case of the 1:1 mixture of ^**14N**^ **1­(PF**_**6**_ **)**_**2**_ and ^**15N**^ **1­(PF**_**6**_ **)**_**2**_ (Figure 5b), ^14,15^N_2_ and ^15,15^N_2_ are observed with an intensity ratio of roughly 2:1. When using only ^**15N**^ **1­(PF**_**6**_ **)**_**2**_ (Figure 5c), ^15,15^N_2_ but no ^14,15^N_2_ is observed. This confirms the formation of N_2_ from the NH_3_ ligands of **1­(PF**_**6**_ **)**_**2**_ upon oxidation, likely as a product of hydrazine disproportionation. Furthermore, the occurrence of mixed-isotopic ^14,15^N_2_ in the expected 2:1 ratio when using a mixture of ^**14N**^ **1­(PF**_**6**_ **)**_**2**_ and ^**15N**^ **1­(PF**_**6**_ **)**_**2**_ (Figure 5b) supports the proposed bimolecular mechanism (Scheme 4).

**Figure 5:**
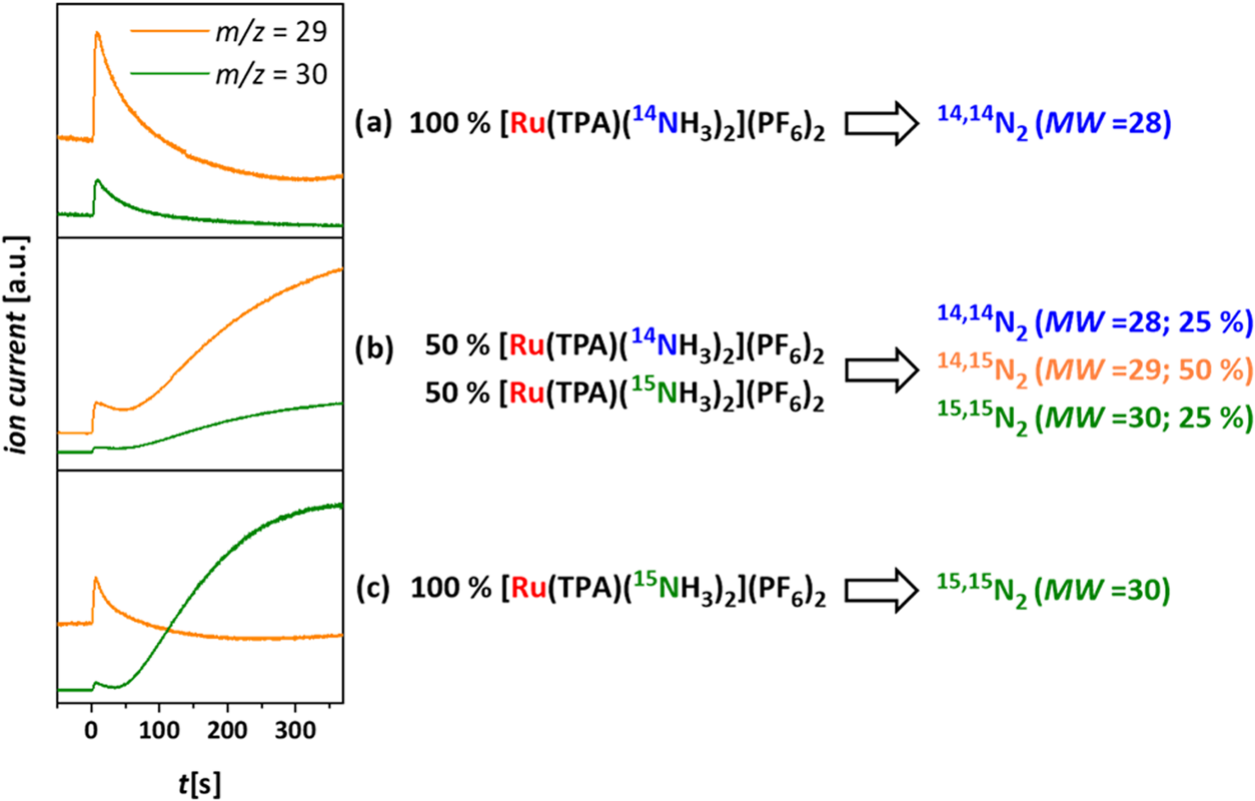
Mass spectrometric analysis of gaseous products after reaction of **1­(PF**_**6**_ **)**_**2**_ with 1.0 equiv of *Magic Blue* in MeCN using (a) only ^**14N**^ **1­(PF**_**6**_ **)**_**2**_, (b) a 1:1 mixture of ^**14N**^ **1­(PF**_**6**_ **)**_**2**_ and ^**15N**^ **1­(PF**_**6**_ **)**_**2**_, and (c) only ^**15N**^ **1­(PF**_**6**_ **)**_**2**_. The evolution of ion currents at *m*/*z* = 29 and 30 is shown, being assigned to ^14,15^N_2_ and ^15,15^N_2_, respectively.

While we were not able to isolate the proposed hydrazine-bridged diruthenium intermediate **5** ^**4+**^, we have independently prepared the complex [Ru­(TPA)­(N_2_H_4_)_2_]­(BPh_4_)_2_ (**3­(BPh**_**4**_ **)**_**2**_) bearing two terminal hydrazine ligands. It was synthesized by exchanging the anions of **1­(PF**_**6**_ **)**_**2**_ to obtain **1­(BPh**_**4**_ **)**_**2**_, which was then reacted with 1 M N_2_H_4_ in THF at reflux for 1 day and characterized using NMR spectroscopy (see SI, section 2.4). Yellow block-shaped crystals suitable for X-ray diffraction analysis were grown by layering a solution of **3­(BPh**_**4**_ **)**_**2**_ in MeCN with Et_2_O at rt under N_2_ atmosphere. The molecular structure of the cation **3** ^**2+**^ is shown in Figure 3 (bottom left). In this case, the two Ru–N^N2H4^ bond lengths are significantly different, the one *trans* to the bridgehead sp^3^ N atom of TPA being longer (2.157(2) vs 2.129(2) Å) but similar to the two Ru–NH_3_ bond lengths in **1** ^**2+**^ (see Figure S36). Under strictly inert conditions, **3­(BPh**_**4**_ **)** is stable in MeCN solution but decomposes upon exposure to air over the course of several hours (see SI, section 4). While decomposition products could not be identified, this reactivity suggests redox processes involving the hydrazine ligands, which may render related hydrazine-bridged species, such as **5** ^**4+**^, inherently unstable.

### Electrochemical Investigations

Cyclic voltammetry (CV) experiments were performed to gain a deeper understanding of processes during oxidation of **1­(PF**_**6**_ **)**_**2**_. Figure 6 shows the CVs of complexes **1­(PF**_**6**_ **)**_**2**_ (blue), **2­(PF**_**6**_ **)**_**2**_ (red), and **XI­(PF**_**6**_ **)**_**2**_ (gray), all measured in MeCN with 0.1 M TBAPF_6_ as a supporting electrolyte at a scan rate of 100 mVs^–1^. Complexes **2­(PF**_**6**_ **)**_**2**_ and **XI­(PF**_**6**_ **)**_**2**_ show reversible redox processes at half-wave potentials of 0.62 and 0.90 V vs Fc^+/0^, respectively. The CV of complex **1­(PF**_**6**_ **)**_**2**_ shows a redox event at a half-wave potential of 0.40 V vs Fc^+/0^.

**Figure 6:**
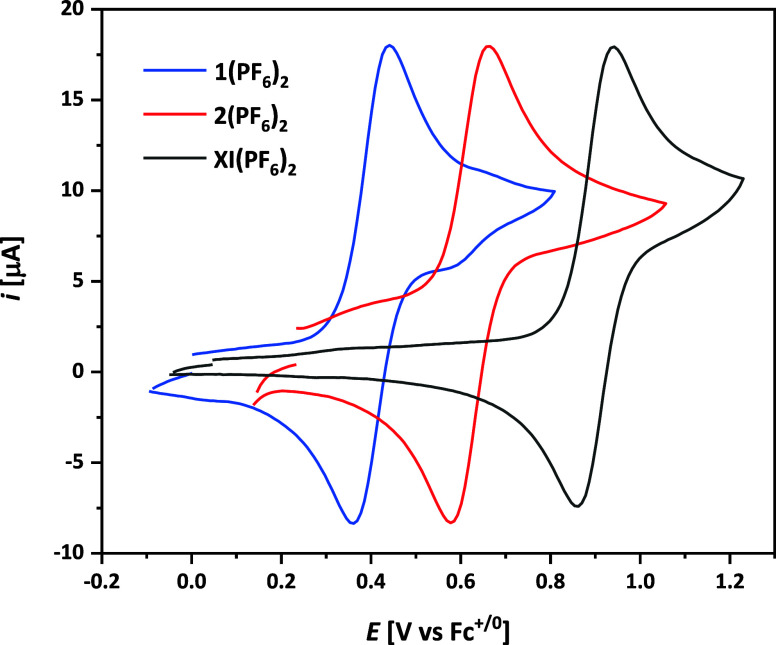
CVs of **1­(PF**_**6**_ **)**_**2**_, **2­(PF**_**6**_ **)**_**2**_, and **XI­(PF**_**6**_ **)**_**2**_ (each 1.0 mM) in MeCN with 0.1 M TBAPF_6_ as the supporting electrolyte at a scan rate of 100 mVs^–1^.

To confirm the one-electron nature of this oxidation process, we have determined the diffusion coefficient of **1** ^**2+**^ as *D* = 9.67 × 10^–6^ cm^2^s^–1^ using DOSY NMR (see SI, Figure S10). When plugging this value into the Randles–Ševčík equation (eq 2)^43^ together with peak current *i*_p_ = 18 μA, electrode surface area *A* = 7 mm^2^, complex concentration *c* = 1.0 mM, scan rate ν = 100 mVs^–1^, temperature *T* = 298 K, Faraday constant *F*, and gas constant *R*, the number of electrons transferred can be obtained as *n* = 0.98 ≈ 1.

2 $$i_{p}=0.446\cdot n\cdot F\cdot A\cdot c\cdot {\left(\frac{n\cdot F\cdot \nu \cdot D}{R\cdot T}\right)}^{1/2}$$

In the CV of **1­(PF**_**6**_ **)**_**2**_, an additional redox feature appears at a slightly higher potential of around 0.6 V vs Fc^+/0^, which corresponds to the oxidation potential of **2­(PF**_**6**_ **)**_**2**_. This observation is in line with the formation of **2­(PF**_**6**_ **)**_**2**_ upon chemical oxidation of **1­(PF**_**6**_ **)**_**2**_. In fact, when varying the scan rate of the CV measurement (Figure 7, top), the second redox feature disappears when increasing the scan rate and gets more prominent at lower scan rates. This supports the proposed *EC* mechanism as depicted in Scheme 4. When recording the CV using different concentrations of **1­(PF**_**6**_ **)**_**2**_ but at a constant scan rate of 100 mVs^–1^ (Figure 7, bottom), the second redox feature assigned to **2­(PF**_**6**_ **)**_**2**_ becomes more prominent for higher complex concentrations. This is consistent with the proposed bimolecular nature of the follow-up chemical step of the *EC* process, leading to the formation of **2­(PF**_**6**_ **)**_**2**_. Although the chemical oxidation of **2­(PF**_**6**_ **)**_**2**_ delivered **XI­(PF** **_6_)**_**2**_, no corresponding feature is observed in the CV of **2­(PF**_**6**_ **)**_**2**_, which indicates that the reaction leading to **XI­(PF**_**6**_ **)**_**2**_ proceeds much more slowly than the time scale of the CV experiment. However, while bulk electrolysis of **1** ^**2+**^ at 0.51 V vs Fc^+/0^ selectively delivered **2** ^**2+**^, electrolysis at 0.65 V vs Fc^+/0^ gave mostly **XI** ^**2+**^ (see SI, section 9.1).

**Figure 7:**
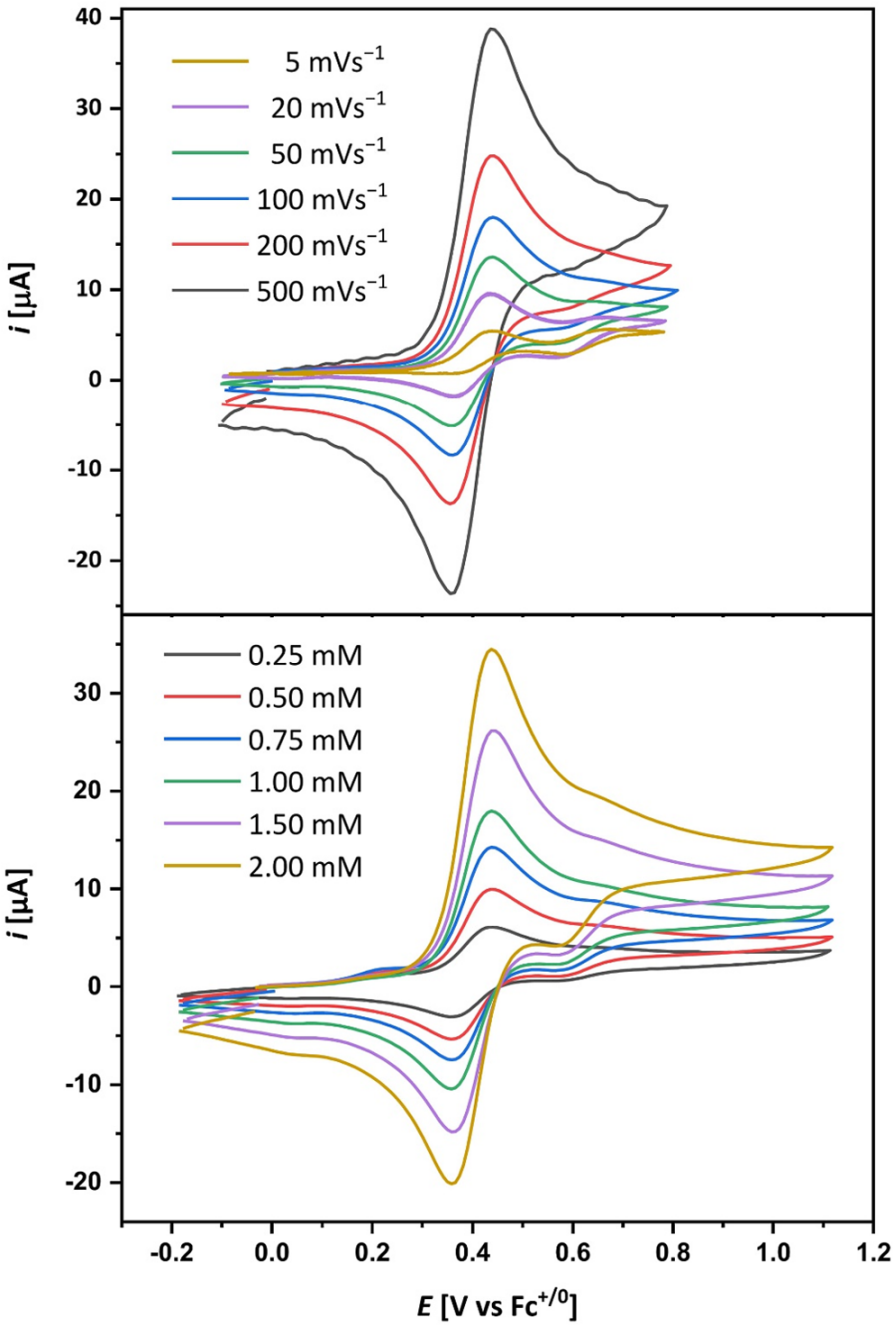
CVs of **1­(PF**_**6**_ **)**_**2**_ in MeCN with 0.1 M TBAPF_6_ as the supporting electrolyte at a concentration of 1.0 mM and various scan rates (top) and at a scan rate of 100 mVs^–1^ and various concentrations (bottom).

In order to confirm the consistency between the suggested mechanism depicted in Scheme 4 and the shape of the CV of **1­(PF**_**6**_ **)**_**2**_, we have performed numerical CV simulations (for details, refer to SI, section 6.4). For this purpose, the mechanism was simplified by summarizing the bimolecular coupling and the subsequent disproportionation in a single chemical step:

$$E_{1},1^{2+}\to 1^{3+}+e^{-}$$

$$C_{1},1^{3+}\to 4^{2+}+H^{+}$$

$$C_{2},2\;4^{2+}\to \to \to 2\;2^{2+}$$

$$E_{2},2^{2+}\to 2^{3+}+e^{-}$$

Figure 8 shows the comparison between the experimental CV of **1­(PF**_**6**_ **)**_**2**_ at a scan rate of 100 mVs^–1^ (gray) and the simulated CV (red; for simulations at various scan rates, refer to the SI, Figures S47–S51). The general shape of the CV could only be reproduced by considering the deprotonation step C_1_ as rate determining with a relatively low first-order rate constant of *k*_1_ = 0.055 s^–1^, while the follow-up intermolecular chemical step C_2_ has a large second-order rate constant (*k*_2_ = 15 × 10^3^ M^–1^s^–1^). This is in accordance with findings from a kinetic analysis of the chemical oxidation of **1­(PF**_**6**_ **)**_**2**_ with *Magic Blue* using UV–vis spectroscopy (see SI, section 7), which are consistent with a first-order rather than second-order dependence of the formation of **2** ^**2+**^ on the concentration of **1** ^**3+**^.

**Figure 8:**
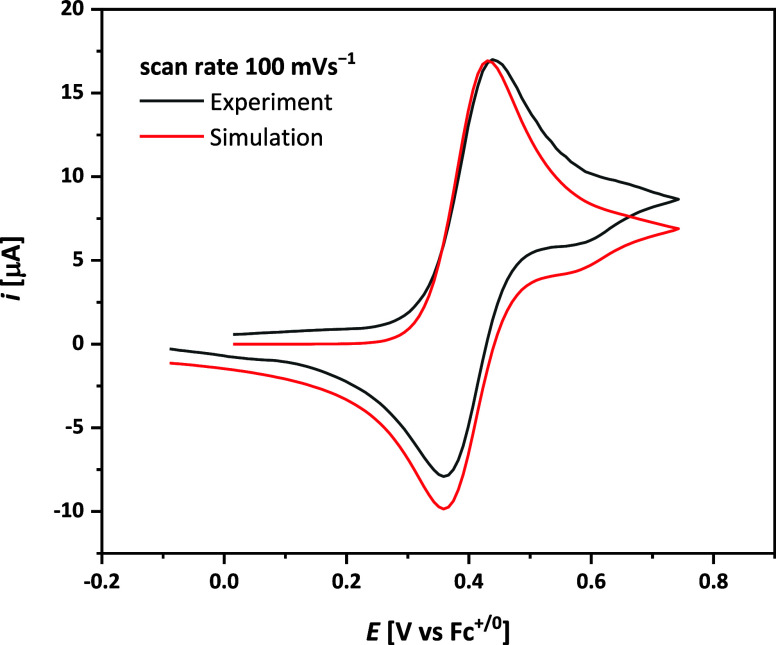
Experimental (gray) and simulated (red) CV of complex **1­(PF**_**6**_ **)**_**2**_ (1.0 mM) in MeCN with 0.1 M TBAPF_6_ as the supporting electrolyte at a scan rate of 100 mVs^–1^ (see SI, section 6.4 for details).

UV/vis spectroelectrochemistry (UV/vis-SEC) was carried out to further support the mechanistic hypothesis. A slow CV experiment (scan rate 5 mVs^–1^) was performed while monitoring spectral changes at the Pt honeycomb working electrode. Figure 9 (top left) shows the obtained voltammogram. While qualitatively exhibiting the same feature as the CVs measured using a GC working electrode (Figure 7), this CV reveals a completed *EC* process leading to a chemically fully reversible second wave, while the first wave in the anodic scan lost its corresponding reduction peak in the cathodic scan. When ramping up the potential, two consecutive processes (a) and (b) are observed (Figure 9, top and middle right), both distinguished by characteristic isosbestic points at (a) 257 and 323 nm and (b) 254 and 311 nm, respectively. According to the mechanism discussed before, they are assigned to (a) the formation of **2** ^**2+**^ from **1** ^**2+**^ and (b) the oxidation of **2** ^**2+**^ to **2** ^**3+**^. After reversing the direction of the CV scan and progressing to more reductive potentials, a process (c) is observed (Figure 9, bottom right) with isosbestic points at 254 and 309 nm and a reversal of the spectral changes of process (b), indicating the reduction of **2** ^**3+**^ to **2** ^**2+**^. Figure 9 (bottom left) summarizes the UV/vis spectra at the start of the experiment and after completion of each process. The lower intensity of the spectrum assigned to **2** ^**2+**^ after process (a) compared to that after process (c) is most likely due to incomplete transformation of **1** ^**2+**^ to **2** ^**2+**^ on the experimental time scale, as the spectra were acquired during a progressing CV scan.

**Figure 9:**
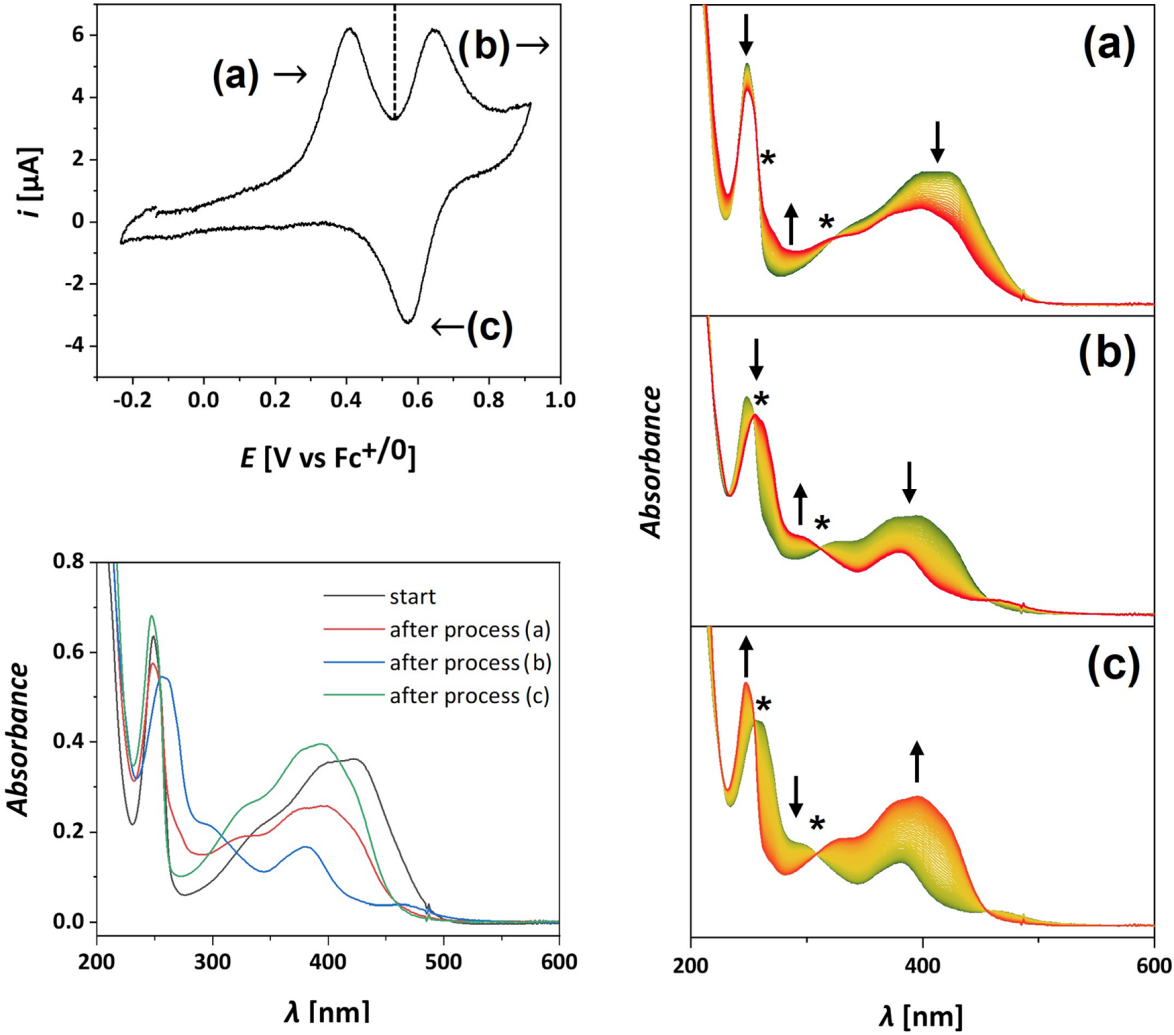
UV/vis-SEC investigation of complex **1­(PF**_**6**_ **)**_**2**_ by performing a slow CV scan (scan rate, 5 mVs^–1^) while recording a UV/vis spectrum each second. The obtained voltammogram is shown on the top left. Subsequently, observed spectral processes are plotted individually from top to bottom on the right. A summary of observed spectra is shown on the bottom left. Isosbestic points are indicated by asterisks.

Since the proposed mechanism (Scheme 4) involves a potentially coupled electron and proton transfer, the influence of bases of different strengths on the CV of complex **1­(PF**_**6**_ **)**_**2**_ was investigated. According to the Bordwell equation (eq 3), increasing the basicity of an external base lowers the oxidation potential when the electron transfer is proton-coupled.^44^

3 $$BDFE_{sol}=1.37pK_{a}+23.06E^{0}+C_{G,sol}$$

A selection of 16 suitable bases in a p*K*_a_ range from 6.89 to 26.02 in MeCN (see SI, section 6.2) was employed by adding 1.05 equiv of the respective base before measuring the CV of **1­(PF**_**6**_ **)**_**2**_. Depending on the individual basicity, the voltammetric response can be influenced drastically (for plots of all measured CVs, refer to the SI, Figures S41–S43). Importantly, the first oxidation wave is shifted to lower potentials when using medium or strong bases. Figure 10 shows a plot of the half-wave potential of the first observed redox process vs the p*K*_a_ value of the respective base. For weak bases, no influence is observed; the half-wave potential of the first wave is 0.41 V vs Fc^+/0^ and thus equal to that of **1­(PF**_**6**_ **)**_**2**_ in the absence of any external base. For stronger bases, the half-wave potential decreases linearly with the p*K*_a_ of the base. A linear regression gives a slope of –40 mV/p*K*_a_, close to (but slightly differing from) the value of –59 mV/p*K*_a_ ideally expected for a 1e^–^/1H^+^ process. This difference might be explained by the involvement of proton and electron transfers also in a chemical follow-up step, for instance, involving the *in situ* formed complex **2** ^**2+**^. The intersection between the two regimes in Figure 12 occurs at p*K*_a_ = 12.6, which thus displays the p*K*_a_ of the oxidized, nondeprotonated species [Ru­(TPA)­(NH_3_)_2_]^3+^ (**1** ^**3+**^).

**Figure 10:**
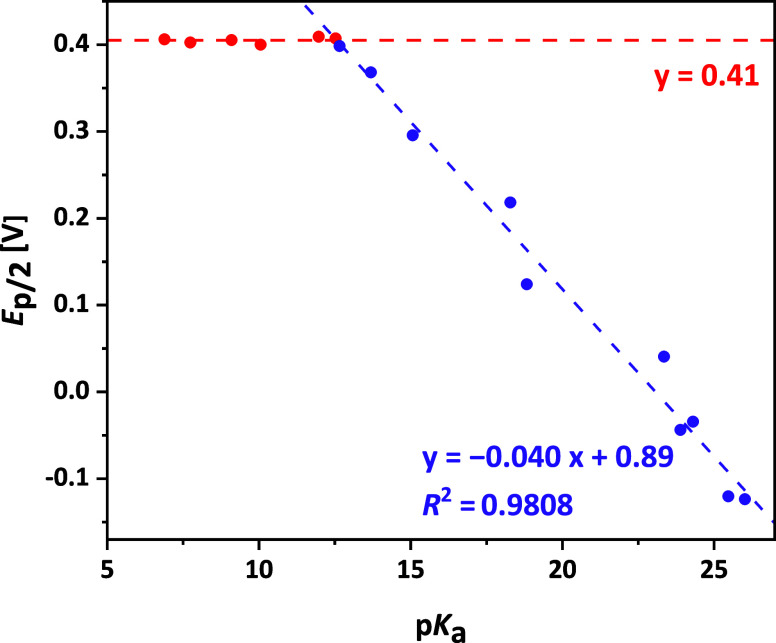
Plot of half-wave potential E_p/2_ in the CV of **1­(PF**_**6**_ **)**_**2**_ vs the p*K*_a_ of the added external base.

### Electrocatalytic Ammonia Oxidation

We then investigated the electrochemical response of complexes **1­(PF**_**6**_ **)**_**2**_ and **2­(PF**_**6**_ **)**_**2**_ in the presence of an excess of ammonia by means of CV in order to examine their suitability as electrocatalysts for the AOR. Figure 11 shows the CVs of 1 mM solutions of complexes **1­(PF**_**6**_ **)**_**2**_ (blue) and **2­(PF**_**6**_ **)**_**2**_ (red) in 1.3 M NH_3_/MeCN as well as the CV of an identical 1.3 M NH_3_/MeCN solution in the absence of any complex (green) as a control experiment. The oxidation waves of both complexes are shifted cathodically by ∼0.4 V compared to the CVs recorded without NH_3_ (Figure 6), being consistent with the proposed PCET nature of the oxidation process. For both waves, the peak currents (53 μA and 44 μA, respectively) are significantly increased compared to the one-electron oxidations in the absence of NH_3_ (18 μA), pointing toward the appearance of a catalytic event at potentials much below the noncatalytic oxidation of NH_3_ at the glassy carbon working electrode (green CV curve).

**Figure 11:**
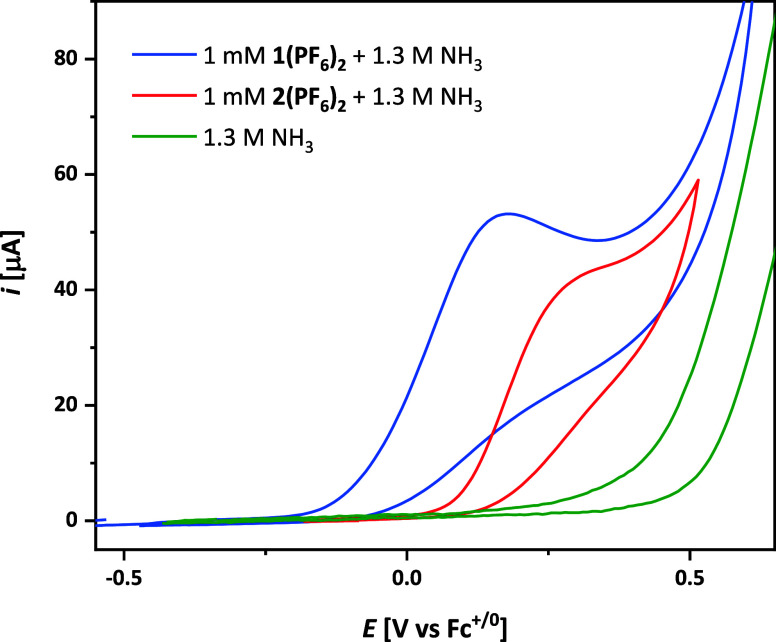
CVs of 1 mM solutions of **1­(PF**_**6**_ **)**_**2**_ (blue) and **2­(PF**_**6**_ **)**_**2**_ (red) in 1.3 M NH_3_/MeCN and of 1.3 M NH_3_/MeCN in the absence of any complex (green), all with 0.1 M TBAPF_6_ as the supporting electrolyte at a scan rate of 100 mVs^–1^.

Varying the concentration of ammonia from 0.61 to 1.3 M (Figure 12) while keeping the concentration of **1­(PF**_**6**_ **)**_**2**_ at 1 mM reveals no strong influence on the catalytic current under the given electrochemical conditions within the tested concentration range. The observation of pseudo-zero-order kinetics with respect to substrate concentration is plausible when dealing with a large substrate excess and can be related to a saturation of the active centers or mass transport limitation.

**Figure 12:**
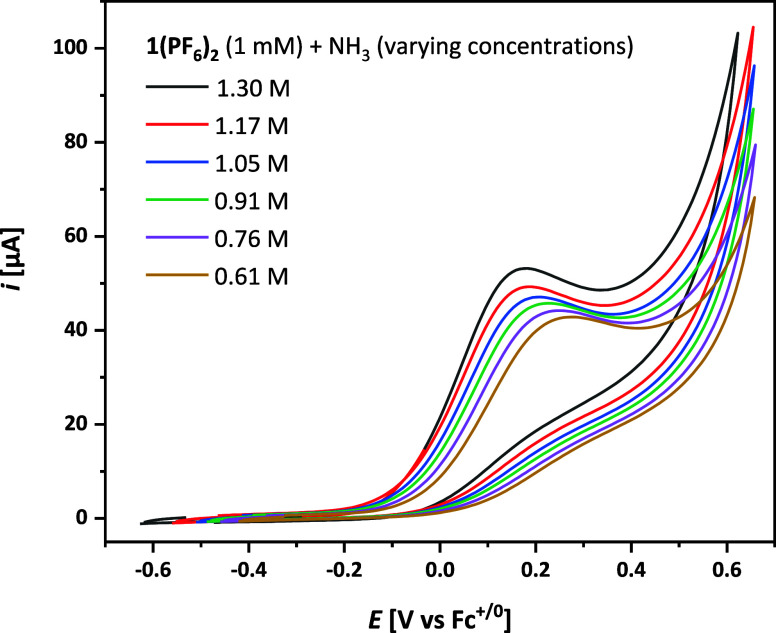
Dependence of the CV response on the ammonia concentration with 1 mM **1­(PF**_**6**_ **)**_**2**_ in MeCN and 0.1 M TBAPF_6_ as a supporting electrolyte at a scan rate of 100 mVs^–1^.

Varying the concentration of **1­(PF**_**6**_ **)**_**2**_ from 0.25 mM to 2.00 mM while keeping the ammonia concentration at 1.3 M leads to a change of the catalytic current (Figure 13, top), which is observed to scale linearly with catalyst concentration (Figure 13, bottom), indicating a first-order dependence. This is in accordance with the deprotonation being the rate-determining step within the suggested bimolecular mechanism (Scheme 4), as it was deduced from CV data simulation (vide supra).

**Figure 13:**
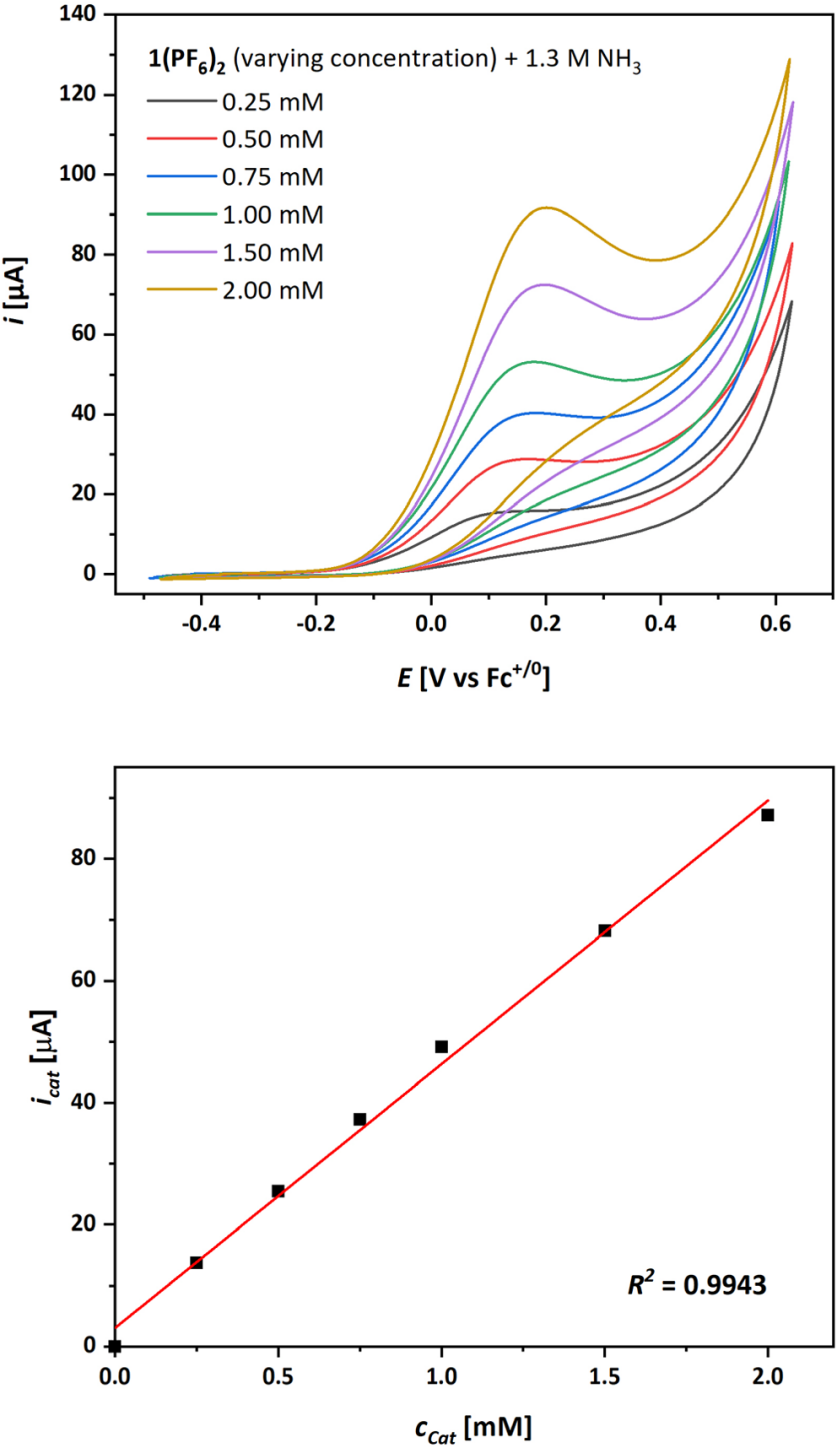
Dependence of the CV response on the concentration of **1­(PF**_**6**_ **)**_**2**_ in 1.3 M NH_3_/MeCN with 0.1 M TBAPF_6_ as the supporting electrolyte at a scan rate of 100 mVs^–1^ (top) and the plot of the catalytic current versus the catalyst concentration (bottom).

For complex **1­(PF**_**6**_ **)**_**2**_, the onset potential of the catalytic wave was determined as −0.09 V vs Fc^+/0^ and its half-wave potential as 0.02 V vs Fc^+/0^. For complex **2­(PF**_**6**_ **)**_**2**_, the onset and half-wave potentials of the catalytic wave were found to be 0.10 and 0.18 V vs Fc^+/0^, respectively (for a description of the procedure of determining onset potentials, refer to the SI, section 6.3). Across the literature, the overpotential of the electrocatalytic processes is inconsistently determined from either onset or half-wave potentials although the use of half-wave potentials is occasionally considered the most precise methodology.^45^ Since the thermodynamic potential for the oxidation of ammonia in MeCN was reported as −0.94 V vs Fc^+/0^,^12,46^ the overpotential for catalyst **1­(PF**_**6**_ **)**_**2**_ is 0.85 V (with respect to onset potential) or 0.96 V (with respect to half-wave potential). Both values fall within the viability zone of a MeCN-based *Direct Ammonia Fuel Cell* (DAFC) as defined by Berry and co-workers,^47^ which requires the oxidative overpotential to be lower than 1.17 V to achieve a net power output when coupling the AOR to the Oxygen Reduction Reaction (ORR).

### Controlled Potential Electrolysis

Controlled potential electrolysis (CPE) experiments were performed to confirm the activity of **1­(PF**_**6**_ **)**_**2**_ as a homogeneous electrocatalyst for the AOR. These experiments were performed in a two-compartment electrochemical cell using a glassy carbon foam as the working electrode and a platinum coil as the counter electrode to facilitate H_2_ evolution by cathodic proton reduction (for details, refer to the SI, section 9.2). CPE of a 1 mM solution of **1­(PF**_**6**_ **)**_**2**_ in 1.3 M NH_3_/MeCN with TBAPF_6_ as the supporting electrolyte was performed at 0.4 V vs Fc^+/0^, which corresponds to a potential at which both catalytic AOR processes involving **1** ^**2+**^ or *in situ* formed **2** ^**2+**^, respectively, are expected to proceed, but noncatalytic NH_3_ oxidation at the glassy carbon working electrode is negligible. Over the course of 1 h, a total charge of around 6.5 C was transferred: three independent runs (see SI, section 9.2) yielded 7.3, 5.2, and 6.8 C, respectively. The observed variation is attributed to small, difficult-to-control differences in the effective surface area of the glassy-carbon-foam electrode. Control experiments were conducted to exclude significant background processes: when performing the CPE under the same conditions, but in the absence of complex **1­(PF**_**6**_ **)**_**2**_, the charge transferred over 1 h amounts to 0.47 C only, indicating the crucial role of **1­(PF**_**6**_ **)**_**2**_ as an ammonia oxidation electrocatalyst.

A gas chromatographic analysis of the headspace of the electrolysis cell after CPE was performed to identify and quantify gaseous reaction products (for details on the experiment and data analysis, refer to the SI, section 10). H_2_ and N_2_ were formed in a molar ratio of 2.6 (3 would ideally be expected for the oxidation of NH_3_; the deviation is likely caused by the diffusive loss of some H_2_ from the electrolysis cell) with a turnover number (TON) of 6 (molecules N_2_ formed per molecule of the catalyst) and a Faradaic efficiency (FE) of 90% (for details on the calculation of TON and FE, refer to the SI, section 11). In order to confirm that N_2_ derives from ammonia, a CPE experiment at 0.4 V vs Fc^+/0^ was performed in the presence of ^15^NH_3_, and the formation of ^15^N_2_ was evidenced by mass spectrometric analysis of the headspace (SI, section 12).

To exclude the occurrence of heterogeneous catalytic processes after possible degradation of **1­(PF**_**6**_ **)**_**2**_ at the electrode surface, leading to the formation of potentially electroactive deposits, a rinse test was performed. For this purpose, the glassy carbon foam working electrode used in a CPE experiment for 1 h at 0.4 V vs Fc^+/0^ was thoroughly rinsed with MeCN and reused for CPE in fresh ammonia solution without complex **1­(PF**_**6**_ **)**_**2**_ under otherwise identical conditions. Over 1 h, a charge of only 0.32 C (instead of around 6.5 C in the presence of **1­(PF**_**6**_ **)**_**2**_) was transferred, corroborating the role of **1­(PF**_**6**_ **)**_**2**_ as a homogeneous electrocatalyst (SI, Figure S63). Furthermore, an XPS analysis of the electrode surface after CPE gave no indication of ruthenium deposition upon electrolysis (SI, section 13).

To identify potential deactivation pathways of catalyst **1­(PF**_**6**_ **)**_**2**_, we analyzed the composition of the reaction solution after CPE by ^1^H NMR spectroscopy. Complexes **2** ^**2+**^ and **XI** ^**2+**^ were found as the main species (SI, Figure S64). Then, complex **XI­(PF**_**6**_ **)**_**2**_ was tested for its ability to exchange MeCN ligands with ammonia when exposed to a large excess of NH_3_ in CD_3_CN. ^1^H NMR spectra were acquired 10 min and again 24 h after the addition of NH_3_ (SI, Figure S55). No spectral changes can be observed, indicating the inertness of coordinated MeCN toward ligand exchange. As compound **XI** ^**2+**^ is not oxidized at the relevant operating potential, it therefore represents a dead end for catalysis under the given experimental conditions.

### Computational Studies

DFT calculations were performed to complement the experimental work and gain further mechanistic insights, particularly regarding the PCET steps involved. All calculations use the B3LYP functional in conjunction with the def2-TZVP (Ru) and def2-svp (C, N, H) basis sets, respectively (for details on the methodology, refer to SI, Section 14). We computed p*K*_a_ values and redox potentials in MeCN for all relevant species [Ru­(TPA)­(NH_*x*_)­(NH_*y*_)]^n+^ to evaluate plausible reaction pathways under the operating conditions. *BDFE* values were derived from computed p*K*_a_ values and redox potentials using the Bordwell equation (eq 3). Figure 14 summarizes possible PCET reaction pathways for complex **1** ^**2+**^. The first oxidation of **1** ^**2+**^ is computed to happen at 0.42 V vs Fc^+/0^, which aligns well with the experimental value of 0.40 V vs Fc^+/0^. Subsequently, two deprotonation events can potentially occur, either on the NH_3_ *trans* (Figure 14 top; p*K*_a_ = 12.1) or *cis* (Figure 14 bottom; p*K*_a_ = 13.4) to the bridgehead sp^3^ N atom of TPA. The computational finding of the *trans*-NH_3_ being more acidic than the *cis*-NH_3_ is in line with the experimental observation that the *trans*-NH_3_ is selectively transformed upon first oxidation of **1** ^**2+**^ to give **2** ^**2+**^. Furthermore, the predicted p*K*_a_ value matches well with the value derived from electrochemical experiments (12.6; Figure 10). Hence, the first *BDFE*_N–H_ of **1** ^**2+**^ is calculated to be 78.9 kcal mol^–1^. When plugging the experimental values for the redox potential of the first oxidation (0.40 V) and the p*K*_a_ value of the oxidized species (12.6) into the Bordwell equation (eq 3), an experimental *BDFE*_N–H_ of 79.1 kcal mol^–1^ results, in excellent agreement with the DFT calculated value. To further support this result, we have reacted **1­(PF**_**6**_ **)**_**2**_ with 1.0 equiv. of the 2,4,6-tri*tert-*butylphenoxy radical (*BDFE*_O–H_ of the corresponding phenol: 77.1 kcal-mol^–1^ ^44^ ). No reaction could be observed at room temperature, setting the experimental lower boundary for the first *BDFE*_N–H_ of **1** ^**2+**^ as 77.1 kcal mol^–1^.

**Figure 14:**
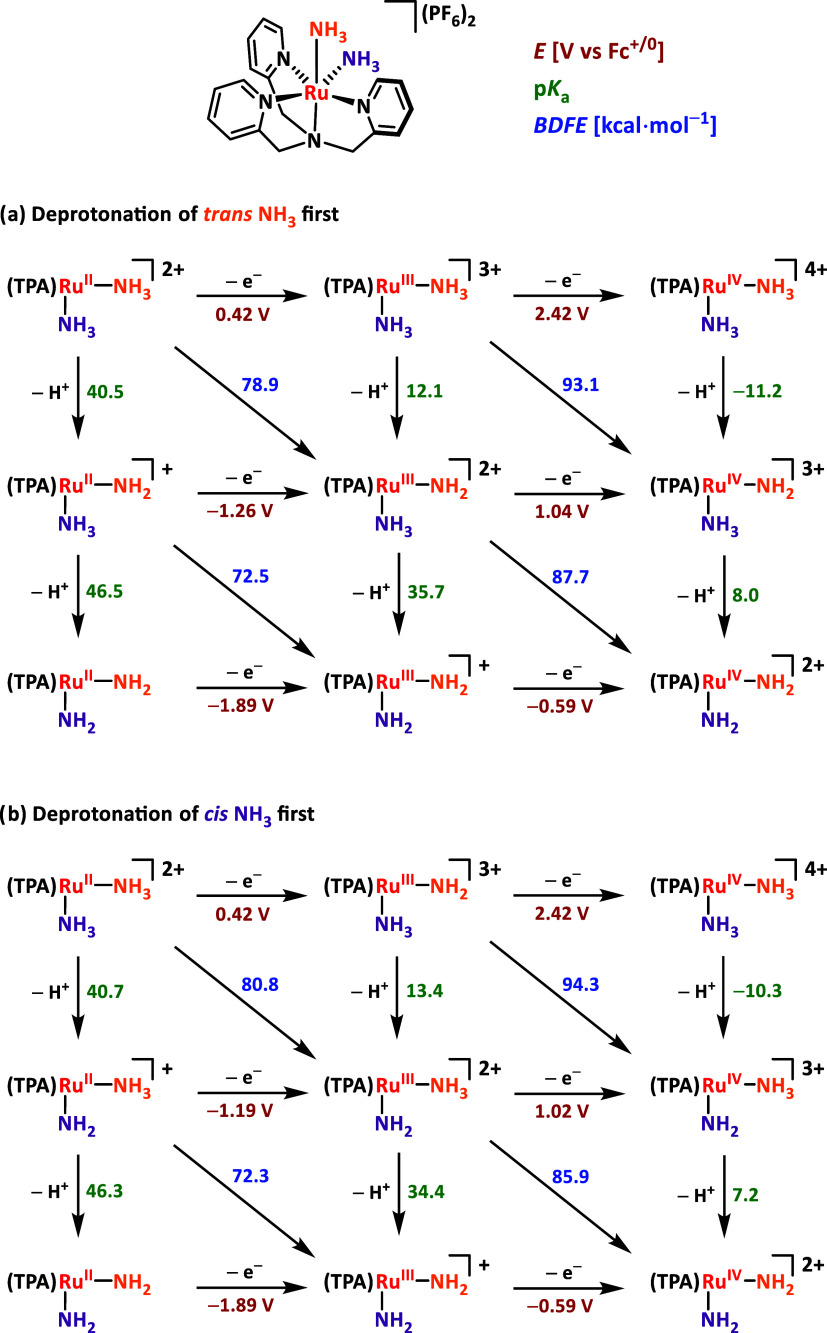
PCET map depicting all relevant intermediates upon stepwise oxidation and deprotonation of **1** ^**2+**^, with first deprotonation occurring on either the NH_3_ *trans* (top) or *cis* (bottom) to the bridgehead sp^3^ N atom of TPA. Computed redox potentials, p*K*_a_ values, and *BDFE*_N–H_ values are given in dark red, green, and blue, respectively.

Thermodynamically unfeasible reaction pathways in the PCET map (Figure 14) can be ruled out by taking into account the p*K*_a_ value of ammonia as the relevant base (experimental 16.5 ^48^) and the working potential during catalysis (0.4 V vs Fc^+/0^). If deprotonation and oxidation occur coupled, the highest *BDFE*_N–H_ that can possibly be overcome amounts to 84.4 kcal mol^–1^ by plugging these values into the Bordwell equation (eq 3). Comparing this number with those obtained from the DFT study (Figure 14) suggests that under catalytic conditions, no further oxidation/deprotonation is expected after the formation of [Ru­(TPA)­(NH_3_)­(NH_2_)]^2+^ (**4** ^**2+**^).

Similarly, we have investigated the first PCET sequence upon oxidation of **2** ^**2+**^ by means of DFT, as summarized in Figure 15. The first oxidation is computed to occur at 0.61 V vs Fc^+/0^ (experimental value: 0.62 V vs Fc^+/0^). Together with the p*K*_a_ value of the oxidized species (13.5), a *BDFE*_N–H_ of 85.1 kcal mol^–1^ is derived for the first PCET process, which is (within DFT accuracy) on the upper edge of N–H bond strengths that can be overcome under experimental catalytic conditions.

**Figure 15:**
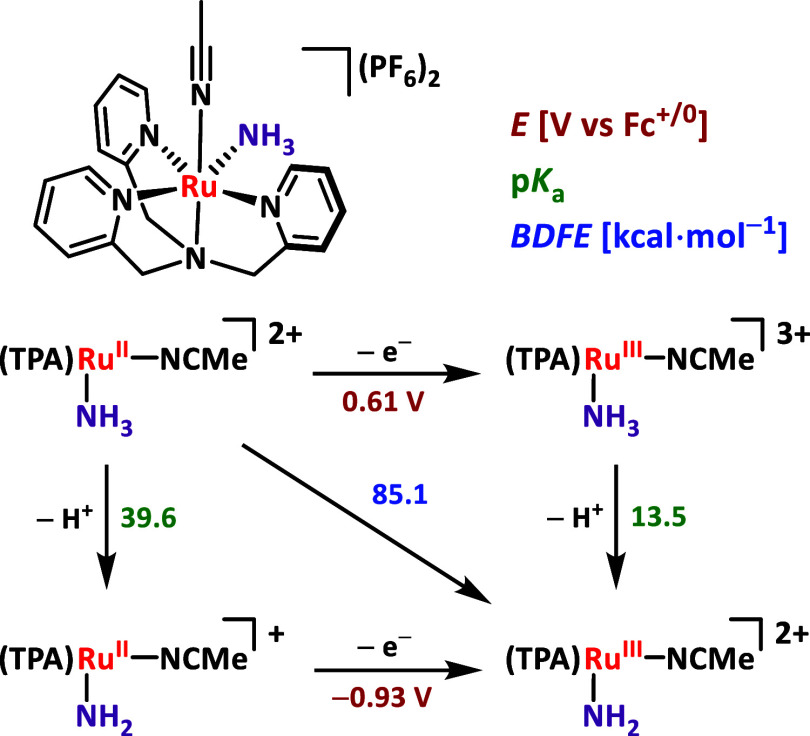
PCET square depicting all relevant intermediates upon the first oxidation and deprotonation of **2** ^**2+**^. Computed redox potentials, p*K*_a_ values, and *BDFE*_N–H_ values are given in dark red, green, and blue, respectively.

The electronic structure of the key intermediate [Ru­(TPA)­(NH_3_)­(NH_2_)]^2+^ (**4** ^**2+**^) was analyzed to validate the proposed mechanism (Scheme 4) that involves an N–N coupling step. The spin density plot of the formal Ru^III^ species **4** ^**2+**^ (Figure 16 left) reveals a significant spin delocalization onto the NH_2_ group. The Mulliken spin population on the N atom of 0.48 as well as the Mayer bond order (Ru–NH_2_) of 1.16 support our proposed description of the {RuNH_2_} fragment as a resonance hybrid of {Ru^III^=NH_2_^–^} and {Ru^II^–NH_2_^•^}. That is, the unpaired electron is delocalized over the Ru and the N atoms of the amido ligand, which is crucial for the following bimolecular radical coupling step. Similar findings for the electronic structure of [Ru­(TPA)­(MeCN)­(NH_2_)]^2+^ (**6** ^**2+**^; spin density plot shown in Figure 16 right; Mulliken spin population on the N atom: 0.56; Mayer bond order of the Ru–NH_2_: 1.22) support the assumption of a bimolecular radical coupling for this intermediate as well.

**Figure 16:**
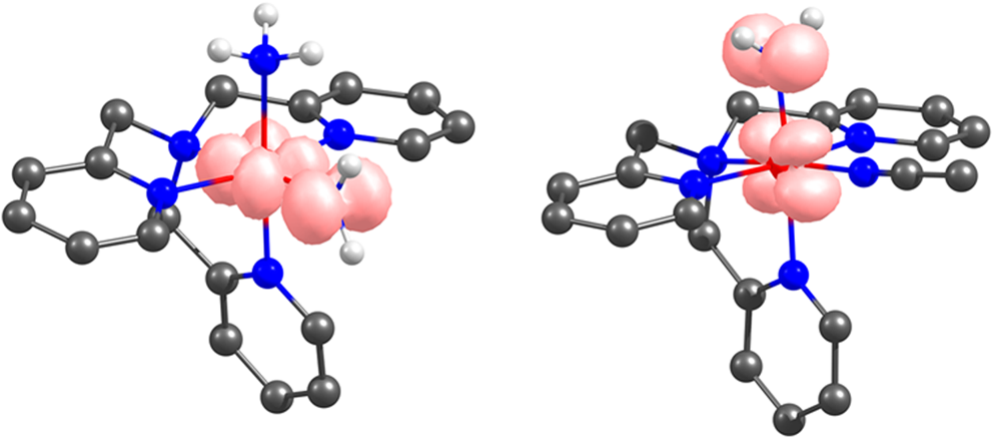
Spin density plots for key intermediates [Ru­(TPA)­(NH_3_)­(NH_2_)]^2+^ (**4** ^**2+**^; left) and [Ru­(TPA)­(MeCN)­(NH_2_)]^2+^ (**6** ^**2+**^; right).

We also computed the transition state for the bimolecular coupling of two molecules **4** ^**2+**^ to form a dimeric complex **5** ^**4+**^. The calculated activation barrier for this process is only 6.2 kcal mol^–1^, consistent with the experimental finding that this step is very fast and, therefore, not rate-determining (vide supra).

### Establishing a Catalytic Cycle

On the basis of the experimental investigations on the chemical oxidation of **1­(PF**_**6**_ **)**_**2**_ and **2­(PF**_**6**_ **)**_**2**_ and their electrochemistry in the absence and presence of NH_3_, as well as complementary computational studies, a mechanism for the electrocatalytic oxidation of ammonia with complex **1­(PF**_**6**_ **)**_**2**_, as shown in Figure 17, can be proposed.

**Figure 17:**
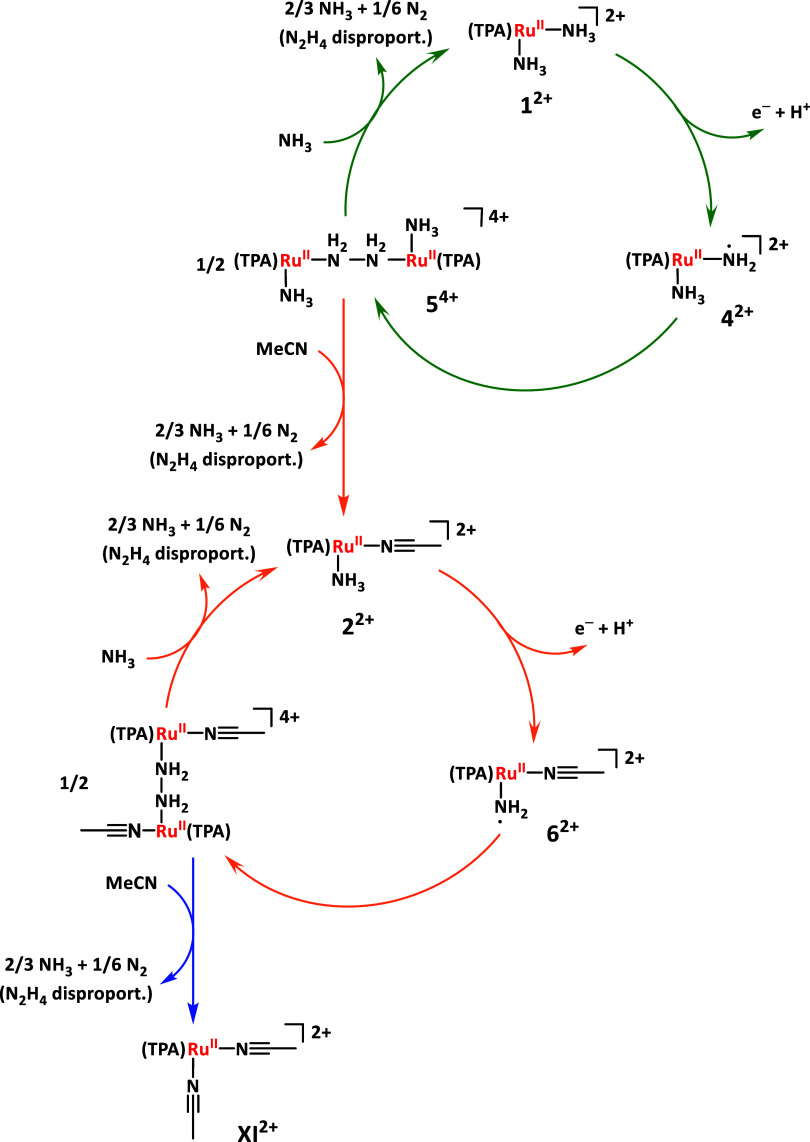
Proposed catalytic cycle for the oxidation of NH_3_ with complex **1** ^**2+**^, including a deactivation pathway via stepwise formation of **2** ^**2+**^ and eventually **XI** ^**2+**^.

In the primary catalytic cycle (green), starting complex **1** ^**2+**^ is oxidized and deprotonated in a strongly coupled 1e^–^/1H^+^ step, with the deprotonation being rate determining for the catalytic cycle. The resulting {Ru­(NH_2_)} species exhibits significant N-centered radical character that promotes N–N bond formation upon bimolecular coupling. The thus formed N_2_H_4_-bridged diruthenium species is proposed to disproportionate spontaneously to give N_2_ and NH_3_. The resulting vacant coordination site at the ruthenium center can now either be occupied by fresh NH_3_ substrate to close the green cycle by reformation of **1** ^**2+**^ or by a solvent molecule (MeCN) to give **2** ^**2+**^. This compound itself is active in a secondary catalytic cycle (orange), undergoing the same mechanistic transformation at the second NH_3_ ligand positioned *cis* to the bridgehead sp^3^ N atom of the TPA. Upon disproportionation of the bridging N_2_H_4_ unit, again either NH_3_ can bind reforming **2** ^**2+**^ or a second MeCN molecule from solvent binds to give bis­(acetonitrile) complex **XI** ^**2+**^, which was shown to be catalytically inactive under the operating conditions (*E* = 0.4 V vs Fc^+/0^) and thus represents a relevant deactivation pathway.

### Comparison with Other AOR Electrocatalysts

Homogeneous AOR electrocatalysts based on Ru have widely been studied over the last years and were, in many cases, thoroughly examined mechanistically. While, in principle, two main pathways for N–N bond formation have been discussed (*ANA* and *I2M*, see Scheme 1), the majority of systems were found to follow *ANA* routes (e.g., complexes **III** and **VI**–**VIII**)^26,29–31^ while examples for *I2M* remain scarce. Complex **II** reported by Nishibayashi and co-workers is an early representative for this reactivity pattern, with bimolecular coupling occurring at the nitrido stage of the PCET sequence,^25^ and both *ANA* and *I2M* were found to compete for **I** ^**2+**^.^35^ We have recently reported the first example of a diruthenium AOR electrocatalyst featuring a highly preorganized dinuclear substrate binding site, which promotes intramolecular *I2M-*type N–N coupling at the early amido stage of the PCET cycle.^32^ This has been explained by spin delocalization over the Ru and N atoms of the {RuNH_2_} fragment, leading to a barrierless bond formation through radical coupling.

In this work, we introduce complex **1­(PF**_**6**_ **)**_**2**_ as an active AOR electrocatalyst and elucidate its mechanism through combined experimental and computational studies. Together with the analogous iron complex [Fe­(TPA)­(NH_3_)_2_]­(OTf)_2_ (**IX­(OTf)**_**2**_) reported by Peters and co-workers,^16^ this study allows, for the first time, a direct comparison between homogeneous AOR electrocatalysts featuring identical ligand spheres but different metal centers (from the same group of the periodic table). The iron complex was shown to mediate the AOR with a fast catalytic rate (*k*_*obs*_ = 3.7 × 10^7^ M^–1^s^–1^) but at a relatively high onset potential of 0.7 V vs Fc^+/0^. While the mechanistic picture remained incomplete, the iron amido species [Fe^III^(TPA)­(NH_3_)­(NH_2_)]^2+^ was identified as the product of the electrochemical 1e^–^ oxidation coupled to a deprotonation event, analogous to the behavior observed for **1** ^**2+**^. However, while we could demonstrate [Ru^III^(TPA)­(NH_3_)­(NH_2_)]^2+^ to undergo rapid bimolecular N–N bond formation due to a significant spin density localized at the NH_2_ unit, the iron analogue does not show catalytic turnover at this stage of the PCET sequence. Instead, an additional oxidation step is required, delivering high-valent intermediates such as [Fe^IV^(TPA)­(NH_3_)­(NH_2_)]^3+^. This species or related oxidation/deprotonation products are likely candidates for the catalytic key intermediate that initiates N–N bond formation via nucleophilic attack of NH_3_ (*ANA*) or the inter- or intramolecular coupling of NH_*x*_ units (*I2M*). In a follow-up work, Peters and co-workers modified their AOR catalyst by replacing the weak-field sp^3^ amine and one pyridine of the TPA ligand with a bipyridine group. This ligand architecture enhances the ligand field splitting, resulting in a higher low-spin contribution at the iron center, which leads to an improved catalyst stability, higher AOR rate, and a lower onset potential of 0.45 V. However, the mechanistic scenario appeared to remain similar to the TPA-based system (**IX** ^**2+**^), requiring at least a 2e^–^ oxidation to achieve catalytic turnover.^20^ The necessity of a second oxidation to reach catalytic turnover is a fundamental difference to the Ru system and accounts for the ∼0.8 V lower catalytic onset potential of **1** ^**2+**^ compared to the Fe-based system **IX** ^**2+**^. Although the presence of two *cis* coordination sites for NH_3_ binding was part of the original catalyst design of **1** ^**2+**^ and **IX** ^**2+**^ to potentially enable intramolecular N–N coupling pathways, no evidence for such reactivity was found for the Ru complex **1­(PF**_**6**_ **)**_**2**_ in the present study.

## Summary and Conclusions

We herein present the novel Ru^II^ complex [Ru­(TPA)­(NH_3_)_2_]­(PF_6_)_2_ (**1­(PF**_**6**_ **)**_**2**_), which has been examined with regard to its oxidation chemistry and has been demonstrated to mediate the electrocatalytic ammonia oxidation reaction (AOR). When reacting **1­(PF**_**6**_ **)**_**2**_ with 1.0 equiv of *Magic Blue* as an oxidant, the formation of [Ru^II^(TPA)­(MeCN)­(NH_3_)]­(PF_6_)_2_ (**2­(PF**_**6**_ **)**_**2**_) was observed. We propose a mechanistic scenario for the oxidation of **1** ^**2+**^ involving the formation of a partially ligand-centered radical intermediate [Ru­(TPA)­(NH_3_)­(NH_2_)]^2+^ (**4** ^**2+**^) that reacts via a bimolecular radical coupling pathway to give a hydrazine-bridged diruthenium species. This undergoes rapid decomposition, resulting in the formation of **2** ^**2+**^, with N_2_ and NH_3_ being released as disproportionation products of the N_2_H_4_ intermediate. The proposed mechanism was evidenced by a bouquet of methods including EPR and NMR spectroscopies, gas-phase mass spectrometry experiments with ^15^N isotope labeling, as well as electrochemical and spectroelectrochemical methods. Numerical simulation of CV data revealed the deprotonation of **1** ^**3+**^ as the rate-determining step. An investigation on the influence of various bases on the CV of **1** ^**2+**^ allowed us to derive a p*K*_a_ value (12.6) for this deprotonation step. Complex **2­(PF**_**6**_ **)**_**2**_ was found to exhibit similar oxidation chemistry upon treatment with *Magic Blue*, leading to the formation of the literature-known species [Ru^II^(TPA)­(MeCN)_2_]­(PF_6_)_2_ (**XI­(PF**_**6**_ **)**_**2**_).

When performing CV experiments on **1­(PF**_**6**_ **)**_**2**_ or **2­(PF**_**6**_ **)**_**2**_ in the presence of excess ammonia, the emergence of a catalytic current can be observed in both cases. For complex **1­(PF**_**6**_ **)**_**2**_, this current was found to behave first order with respect to the complex concentration. Its catalytic onset potential was determined as −0.09 V vs Fc^+/0^ and its half-wave potential as 0.02 V vs Fc^+/0^, which corresponds to an overpotential of 0.85 V (with respect to onset potential) or 0.96 V (with respect to half-wave potential). Controlled potential electrolysis experiments at 0.4 V vs Fc^+/0^ followed by gas chromatographic headspace analysis evidenced the formation of H_2_ and N_2_ in a molar ratio of 2.6 with a TON of 6 (molecules N_2_ formed per molecule catalyst) after 1 h and a Faradaic Efficiency of 90%. The formation of N_2_ from NH_3_ was further confirmed by a mass spectrometric ^15^N isotope labeling experiment.

A DFT study was conducted to develop a PCET map involving all potential intermediates formed upon oxidation or deprotonation. The analysis of oxidation potentials and p*K*_a_ values allowed the evaluation of possible reaction pathways and ruled out unfeasible routes under the experimental operating conditions. Furthermore, the proposed partially ligand-centered radical character of key intermediates **4** ^**2+**^ and **6** ^**2+**^ could be confirmed by the computational electronic structure analysis. A low energy barrier for the N–N bond formation step of 6.2 kcalmol^–1^ was calculated.

A complete mechanistic scenario was developed based on our combined experimental and computational findings. Starting from complex **1­(PF**_**6**_ **)**_2_, intermolecular N–N bond formation (*I2M-*type mechanism) is featured at an early stage of the potential PCET sequence, viz., already after the first 1H^+^/1e^–^ step when the metal-amido intermediate is formed. Complex **2­(PF**_**6**_ **)**_**2**_, being catalytically active itself, was identified as an important side product of the primary catalytic cycle, while complex **XI­(PF**_**6**_ **)**_**2**_ is an inactive off-cycle product leading to gradual catalyst deactivation.

Having established the mechanistic picture of **1** ^**2+**^ as an AOR electrocatalyst, we were able to draw a direct comparison with the iron analogue [Fe­(TPA)­(NH_3_)_2_]^2+^ (**IX** ^**2+**^) reported by Peters and co-workers.^16^ We found a substantially different mechanistic behavior between the Fe and Ru catalysts despite their identical ligand environments. While the present Ru system undergoes rapid *I2M* at an early stage of the PCET sequence, viz., after 1e^–^/1H^+^ oxidation to the amido species {Ru­(NH_2_)}, due to significant delocalization of the unpaired electron over the Ru and N atoms, the Fe system requires the generation of some Fe^IV^ species for N–N bond formation that likely follows an *ANA* mechanism. Consequently, the new Ru system operates at a significantly reduced catalytic onset potential (−0.09 V for **1** ^**2+**^ vs 0.70 V for **IX** ^**2+**^). By comparison with other Ru AOR electrocatalysts, we found that complexes following this *I2M* reactivity pattern from the amido stage exhibit comparably low catalytic onset potentials. Both our previously reported dinuclear Ru catalyst (onset –0.18 V vs Fc^+/0^)^32^ and complex **I** ^**2+**^ (onset 0.0 V vs Fc^+/0^)^24,35^ were demonstrated to undergo N–N coupling already after 1e^–^/1H^+^ oxidation, promoted by a significant spin density located at the {NH_2_} unit, comparable to **1** ^**3+**^. On the other hand, mononuclear complexes following *I2M*-type pathways at higher oxidation states (such as **II**, onset 0.2 V vs Fc^+/0^),^25^ or undergo *ANA* type mechanisms (such as **III**, onset 0.52 V;^26^ **VI**, onset 0.2 V;^29^ **VII**, onset 0.73 V;^31^ and **VIII**, onset 0.1 V^30^) exhibit an onset potential that is several 100 mV higher. This may provide valuable information for the future rational AOR catalyst design, where enhanced spin delocalization on an NH_3_-derived {NH_2_} unit will favor rapid N–N formation at low onset potentials, ideally promoted in preorganized bimetallic systems to enable intramolecular *I2M* even in dilute solutions or when the catalysts are site-isolated.^32^ An alternative bimetallic approach for electrocatalytic AOR at low overpotentials was reported by Berry and co-workers, where a metal–metal bonded diruthenium unit with three-center Ru–Ru–NH_2_ interaction enables N–N bond formation via an *ANA* mechanism without the need to access higher oxidized Ru-imido or Ru-nitrido species.^47^

While further lowering of the overpotential remains an important challenge, from comparison of the series of known Ru-based AOR electrocatalysts (Figure 1), we conclude that introducing anionic donors into the ligand environment to reduce the overall charge of the complexes and lower the oxidation potentials may lead to counterintuitive effects (e.g., **II, III**, **VI** – **VIII**). This modification will stabilize higher-valent Ru species, thereby diminishing spin localization and N-centered radical character at the Ru^III^ state, ultimately resulting in a change in mechanism to *ANA* and increased onset potentials. Ongoing work in our laboratory aims at ligand design that imparts both low redox potentials of the {Ru^II^(NH_3_)} complex and pronounced delocalization of the unpaired electron onto the N atom after the first PCET step, in the formal {Ru^III^(NH_2_)} intermediate.

For the present system, the identification of **XI­(PF**_**6**_ **)**_**2**_ as an off-cycle product also leaves room for further improvement. The competing binding of fresh NH_3_ substrate vs MeCN solvent molecules could likely be mitigated by performing the catalysis in less coordinating solvents, necessitating changes in catalyst solubility. However, the choice of solvent may also influence the mechanistic scenario, particularly the disproportionation step.

## Supplementary Material





## Notes

Experimental details and synthetic procedures, additional spectroscopic and analytical data, crystallographic information, details of DFT calculations (PDF)
xyz coordinates of DFT calculated structures (PDF)
