# Supplementary material for: Ammonia Oxidation with a Ruthenium Polypyridyl Complex: Mechanism-Guided Approach to Low Overpotential Electrocatalysis
Source: JACS Au. 2026 Jul 11;6(7):4253–68. doi: 10.1021/jacsau.6c00733 (PMC13417302; doi:10.1021/jacsau.6c00733)
Supplement: Supplementary file 1 [file au6c00733_si_001.pdf]

Supporting Information for

Ammonia Oxidation with a Ruthenium Polypyridyl Complex: Mechanism-Guided Approach to Low Overpotential Electrocatalysis

Maximilian Seiß,[1] Elisa Dickgießer,[1] Sebastian Dechert,[1] and Franc Meyer[1,2]*

[1] University of Göttingen, Institute of Inorganic Chemistry, Tammannstr. 4, 37077 Göttingen, Germany;

[2] University of Göttingen, International Center for Advanced Studies of Energy Conversion (ICASEC), Tammannstr. 6, 37077 Göttingen, Germany.

Table of Contents

1. Materials and Methods S2
2. Synthetic Procedures and Characterization S4
   2.1 Complex $[Ru(TPA)(NH_3)_2](PF_6)_2$ ($1(PF_6)_2$) S4
   2.2 Complex $[Ru(TPA)(^{15}NH_3)_2](PF_6)_2$ (**$^{15N}1(PF_6)_2$**) S11
   2.3 Complex $[Ru(TPA)(MeCN)(NH_3)](PF_6)_2$ (**$2(PF_6)_2$**) S12
   2.4 Complex $[Ru(TPA)(N_2H_4)_2](BPh_4)_2$ (**$3(BPh_4)_2$**) S19
   2.5 Complex $[Ru(TPA)(MeCN)_2](PF_6)_2$ (**$XI(PF_6)_2$**) S20
3. Crystallographic Data S21
4. Stability of **$3(BPh_4)_2$** S24
5. EPR Spectroscopy S25
6. Cyclic Voltammetry S26
   6.1 Scan Rate Dependent CVs of Complexes **$2(PF_6)_2$** and **$XI(PF_6)_2$** S26
   6.2 CV of Complex **$1(PF_6)_2$** in the Presence of Various Bases S27
   6.3 CV of Complexes **$1(PF_6)_2$** and **$2(PF_6)_2$** in the Presence of Ammonia S28
   6.4 CV Simulation S30
7. UV-Vis Kinetic Analysis of the Reaction of **$1(PF_6)_2$** with Magic Blue S32
8. Reactivity of **$XI(PF_6)_2$** towards Ammonia S34
9. Controlled Potential Electrolysis S35
   9.1 Electrolysis Experiments with Complex **$1(PF_6)_2$** in the Absence of Ammonia S35
   9.2 Electrolysis Experiments for Electrocatalytic Ammonia Oxidation S36
10. Gas Chromatography S39
11. Calculation of Faradaic Efficiencies and Turnover Numbers S41
12. Labelling Experiment S42
13. Electrode Surface Analysis (XPS) S42
14. Computational Details S43
References S45

1. Materials and Methods

General Considerations.
All experiments were carried out under ambient conditions or under N_2 or Ar atmosphere using standard Schlenk and glovebox techniques if noted. Solvents were degassed by thoroughly sparging with N_2 and dried by passing a column of activated aluminium oxide using the solvent purification system by MBraun. Additionally, acetonitrile was dried over CaH_2. THF and diethylether were dried over sodium and benzophenone. Acetonitrile was stored over activated 3 Å molecular sieves, all other solvents over activated 4 Å molecular sieves. For experiments that required a nitrogen-free environment (nitrogen evolution experiments), solvents were thoroughly sparged with Ar prior to use. Saturated solutions of ammonia in acetonitrile (*c* = 1.3 M) were prepared as reported by Ahmed *et al.*[1] Unless noted otherwise, all reagents were purchased from commercial sources and used without further purification. Complex precursor [$RuCl_2$(*p*-cymene)]$_2$ was prepared as described in the literature.[2,3] ^{15}N-labeled NH_3 solution in MeCN for catalytic experiments was prepared by treating a 1 M solution of ^{15}N-labeled NH_4PF_6 (prepared from labeled NH_4Cl purchased from *Deutero*) in MeCN with 1 equivalent of DBU. All volatile components were condensed into another flask to obtain a ca. 1 M solution of ^{15}N-enriched NH_3 in MeCN. ^{15}N-labeled NH_3 solution in water for the synthesis of labeled complex was prepared by heating a 2:1 mixture of solid ^{15}N-labeled NH_4Cl and $Ca(OH)_2$ and guiding the evolving gas stream into water.

Electrochemistry.
Cyclic Voltammetry (CV) experiments were carried out at room temperature under N_2 atmosphere using a Gamry Reference-620 potentiostat and a three-electrode setup. A glassy carbon disk electrode (3 mm diameter) was used as the working electrode and polished with aluminium oxide (0.05 µm particle size). A platinum coil and a Ag/$TBAPF_6$ (0.1 M)/acetonitrile electrode were used as counter and pseudo-reference electrode, respectively. For experiments in the presence of ammonia, a Ag/$AgNO_3$(0.01 M)/$TBAPF_6$ (0.1 M)/acetonitrile electrode was used as reference electrode. $TBAPF_6$ (0.1 M in acetonitrile) was employed as supporting electrolyte. All redox potentials in this work are reported versus the $Fc^{+/0}$ couple. All measurements were performed applying IR compensation by compensating 90 % of the measured resistance.
CV simulations were carried out using the DigiElch 7 Professional software package by ElchSoft.[4]
Controlled potential electrolysis (CPC) experiments were carried out at room temperature under Ar atmosphere using a Gamry Interface 1010E potentiostat and a three-electrode setup. Glassy carbon foam or a glassy carbon rod was used as working electrode, as specified individually. A platinum coil and a Ag/$AgNO_3$(0.01 M)/$TBAPF_6$ (0.1 M)/acetonitrile electrode were used as counter and reference electrode, respectively. $TBAPF_6$ (0.1 M in acetonitrile) was employed as supporting electrolyte.

NMR spectroscopy.
^{1}H, ^{13}C, ^{31}P, ^{19}F and ^{15}N NMR spectra were recorded on BrukerAvance III HD 300 or 400 or 500 or 600 spectrometers. ^{1}H and ^{13}C chemical shifts are reported in parts per million relative to residual proton and carbon signals of the solvents (MeCN-d$_3$; δ_H = 1.94 ppm; δ_C = 1.32, 118.26 ppm). Phosphorous, fluorine and nitrogen chemical shifts are reported relative to an external H_3PO_4, $CFCl_3$ or $MeNO_2$ standard, respectively.

UV-Vis spectroscopy and spectroelectrochemistry.
UV-Vis spectra were recorded on an Agilent Cary 8454 spectrophotometer equipped with a magnetic stirrer using quartz cuvettes with an attached tube and a septum. UV/Vis spectroelectrochemistry

experiments were carried using the same spectrophotometer and a quartz cell with a platinum honeycomb working electrode, a platinum counter electrode and a Ag/$TBAPF_6$ (0.1 M)/acetonitrile pseudo-reference electrode. $TBAPF_6$ (0.1 M in acetonitrile) was employed as supporting electrolyte.

ESI-Mass Spectrometry.
ESI-Mass spectra were recorded using a Bruker Esquire 3000 Plus spectrometer. HR-ESI-MS spectra were recorded using a Thermo Scientific LTQ Orbitrap XL. Simulated spectra were generated using the Mass Spectrum Simulator by Prot Pi.

Elemental analysis.
Elemental analyses (C, H, N) were performed by the analytical laboratory of the Institute of Inorganic Chemistry at the University of Göttingen using an Elementar VarioEL III instrument.

X-ray crystallography.
X-ray crystallographic data were collected on a BRUKER D8-QUEST diffractometer (monochromated Mo−K_α radiation, λ = 0.71073 Å) by use of ω or ω and ϕ scans at low temperature. The structure was solved with SHELXT[5] and refined on F^2 using all reflections with SHELXL.[6] Face-indexed absorption corrections were performed by the multiscan method with SADABS.[7]

EPR spectroscopy.
EPR spectra were measured with a Bruker E500 ELEXSYS X-band spectrometer equipped with a standard cavity (ER4102ST, 9.43 GHz). The sample temperature was maintained constant with an Oxford instrument Helium flow cryostat (ESP910) and an Oxford temperature controller (ITC-4). The microwave frequency was measured with the built-in frequency counter and the magnetic field was calibrated by using an NMR field probe (Bruker ER035M). EPR spectra were simulated using Easy-Spin.[8]

Gas chromatography.
Gas chromatograms were recorded using a Shimadzu GC-2014 equipped with a Thermal Conductivity Detector (TCD) and a 4 Å molecular sieve column. Methane was used as internal standard for quantitative detection of H_2 and N_2.

Mass spectrometric gas analysis.
Mass spectrometric analysis of gaseous samples were performed using a Pfeiffer Vacuum ThermoStar GSD320.

X-ray photoelectron spectroscopy (XPS).
A Kratos AXIS Supra, SPECS PHOIBOS 100 hemispherical XPS analyzer with a monochromatic X-ray source (SPECS FOCUS 500 monochromator, Al Kα radiation, 1486.74 eV) was applied to determine the elemental composition of the powders as well as of the thin films. The binding energy scale was corrected by shifting the C 1s main peak energy to 284.9 eV with the charge neutralizer. CasaXPS software was used to calculate the atomic percentages of core levels. For all elements, a Shirley background was used. Measurement details: Pass energy: 160 eV; collimation: slot; lens: hybrid; step size: 1 eV; sweep time: 240 s; No. of sweeps: 2; emission current: 15 mA; with charge neutralizer current emission of 0.45 mA.

2. Synthetic Procedures and Characterization

2.1 Complex [Ru(TPA)(NH_3)$_2$](PF_6)$_2$ (1(PF_6)$_2$)

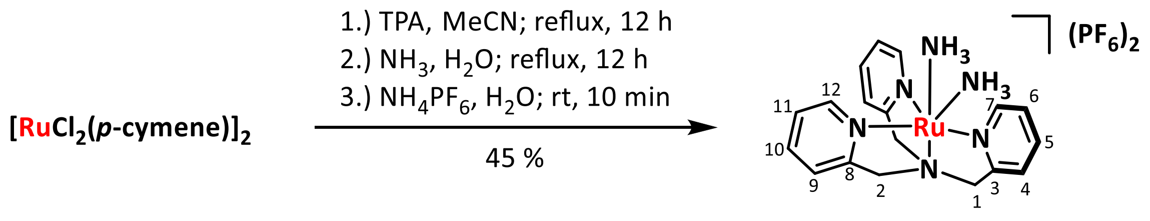

1(PF_6)$_2$

Synthesis.
[RuCl$_2$(*p*-cymene)]$_2$ (320 mg, 0.523 mmol, 0.50 equiv.)[2] was dissolved in MeCN (10 mL). A solution of TPA (305 mg, 1.05 mmol, 2.00 equiv.) in MeCN (10 mL) was added. The mixture was heated to reflux for 12 h. After cooling to room temperature, the solvent was removed under reduced pressure and the solid residue was redissolved in DCM (5 mL) and precipitated by addition of pentane (30 mL). This step was repeated three times and the resulting solid was dried under reduced pressure to yield an orange powder (410 mg). As reported by Whiteoak *et al.*,[3] the obtained substance is not a pure compound but a mixture of the species [Ru(TPA)Cl$_2$], [Ru(TPA)(MeCN)Cl]Cl and [Ru(TPA)(MeCN)]Cl$_2$ that exist in equilibrium in MeCN solution. The powder was suspended in a concentrated, aqueous NH_3 solution (25 %, 50 mL) and heated to reflux for 12 h in a closed reaction vessel. After cooling to room temperature, NH_4PF_6 (1.06 g, 6.56 mmol) was added and the formation of a yellow precipitate was observed. The mixture was stirred for an additional hour after which the solid was filtered off, washed with water and dried under reduced pressure to obtain **1**(PF_6)$_2$ as a yellow solid (339 mg, 0.474 mmol, 45 % over all steps). Yellow block-shaped crystals suitable for X-ray analysis were grown by slow diffusion of Et_2O into a solution of **1(PF_6)$_2$** in MeCN at 5 °C.
^{1}H NMR (400 MHz, MeCN-d$_3$): δ = 8.74 (d, $^3J_{HH}$ = 5.3 Hz, 1 H, 12-H), 8.59 (d, $^3J_{HH}$ = 5.6 Hz, 2 H, 7-H), 7.70 (td, $^3J_{HH}$ = 7.8 Hz, $^4J_{HH}$ = 1.5 Hz, 2 H, 5-H), 7.47 (td, $^3J_{HH}$ = 7.8 Hz, $^4J_{HH}$ = 1.5 Hz, 1 H, 10-H), 7.38 (d, $^3J_{HH}$ = 6.5 Hz, 2 H, 4-H), 7.24 (t, $^3J_{HH}$ = 6.4 Hz, 2 H, 6-H), 7.11 (t, $^3J_{HH}$ = 5.9 Hz, 1 H, 11-H), 6.99 (d, $^3J_{HH}$ = 7.2 Hz, 1 H, 9-H), 4.81 (d, $^2J_{HH}$ = 16 Hz, 2 H, 1-H), 4.69 (d, $^2J_{HH}$ = 16 Hz, 2 H, 1'-H), 4.38 (s, 2 H, 2-H), 2.54 (s, 3 H, NH_3), 2.41 (s, 3H, NH_3) ppm.
^{13}C NMR (101 MHz, MeCN-d$_3$): δ = 165.0 (s, 3-C), 163.3 (s, 8-C), 154.6 (s, 7-C), 153.3 (s, 12-C), 137.5 (s, 5-C), 136.3 (s, 10-C), 125.1 (s, 6-C), 124.1 (s, 11-C), 123.4 (s, 4-C), 120.6 (s, 9-C), 70.5 (s, 1-C), 70.0 (s, 2-C).
^{31}P NMR (203 MHz, MeCN-d$_3$): δ = −144.6 (sept, $^1J_{PF}$ = 708 Hz).
^{19}F NMR (471 MHz, MeCN-d$_3$): δ = −72.8 (d, $^1J_{FP}$ = 707 Hz).
ESI-MS (ESI(+), MeOH): *m/z* = 571.1 [M − PF_6]$^+$, 554.1 [M − NH_3 − PF_6]$^+$, 514.1 [*not identified*], 428.1 [M − NH_3 − 2 PF_6 + F*]$^+$. (** Fluoride likely originates from partial PF_6 hydrolysis in MeOH*)
UV-Vis (MeCN): λ_{max} = 249, 344, 427 nm.
Elem. Anal.: Calculated C, 30.22; H, 3.38; N, 11.75. Found C, 29.70; H, 3.64; N, 11.45 %.

NMR spectra.

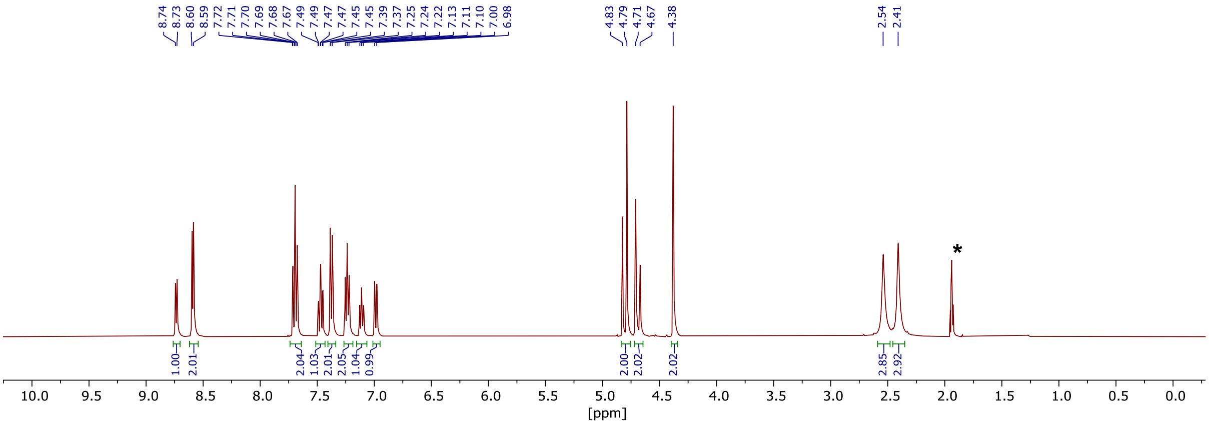

Figure S1. ^{1}H NMR spectrum (400 MHz, MeCN-d_3) of complex **1(PF$_6$)$_2$**. The residual solvent signal (MeCN) is marked with a black asterisk.

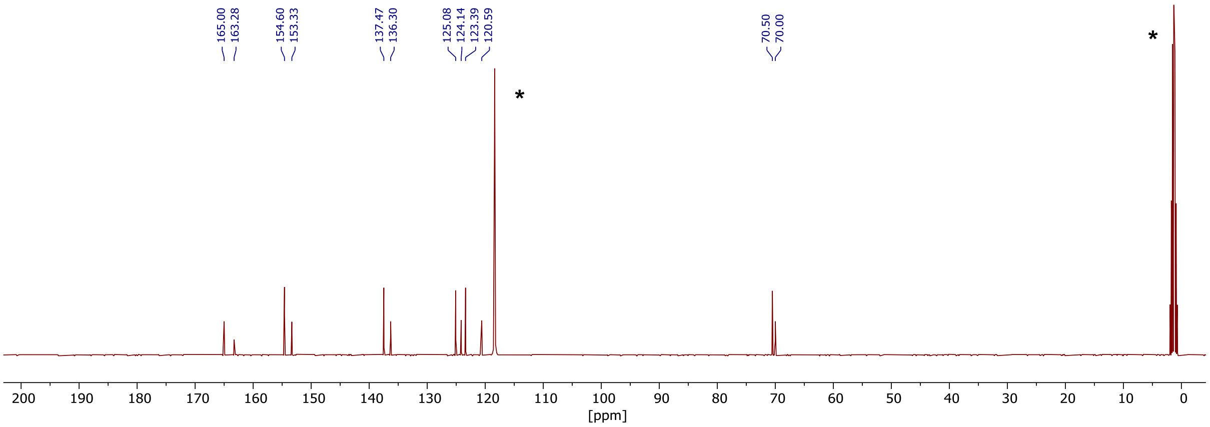

Figure S2. ^{13}C NMR spectrum (101 MHz, MeCN-d_3) of complex **1(PF$_6$)$_2$**. The residual solvent signals (MeCN) are marked with black asterisks.

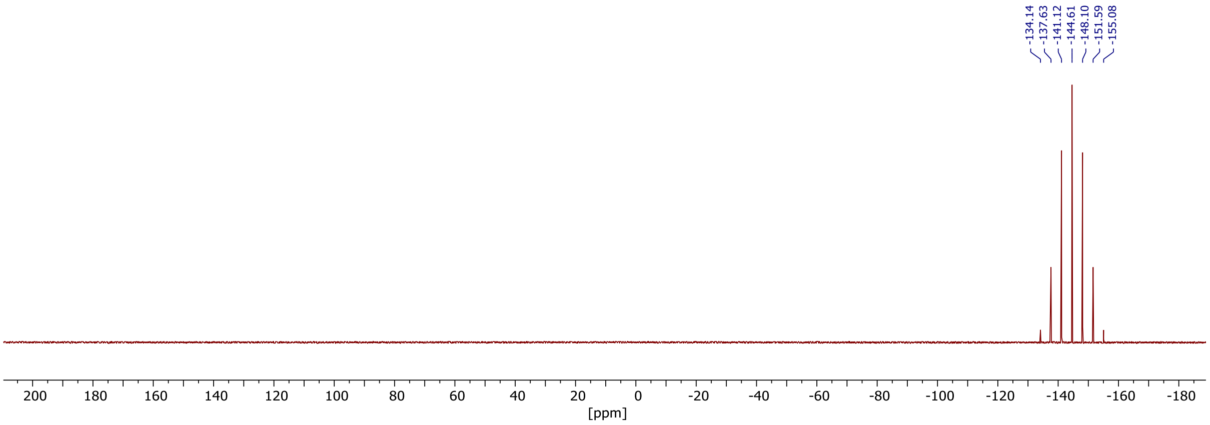

Figure S3. ^{31}P NMR spectrum (203 MHz, MeCN-d_3) of complex **1(PF$_6$)$_2$**.

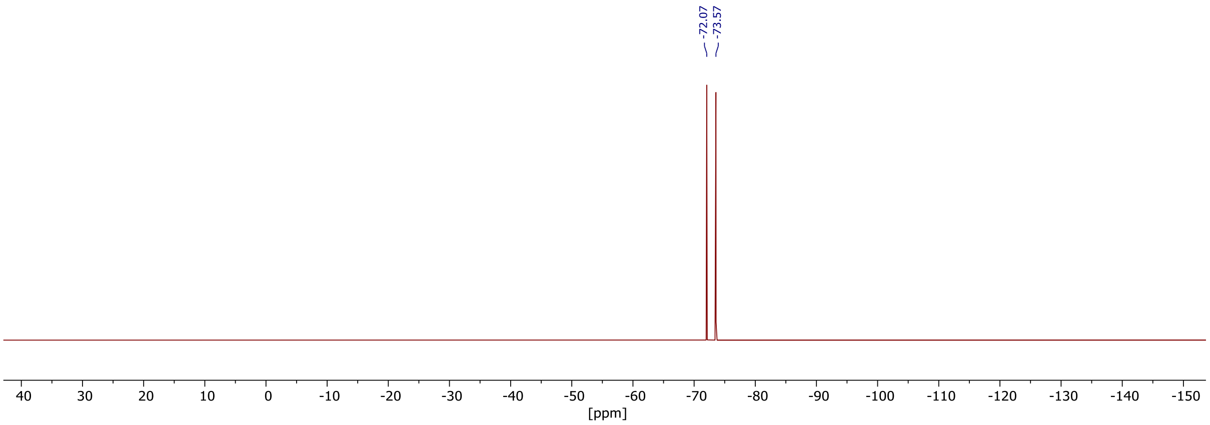

Figure S4. ^{19}F NMR spectrum (471 MHz, MeCN-d$_3$) of complex **1(PF$_6$)$_2$**.

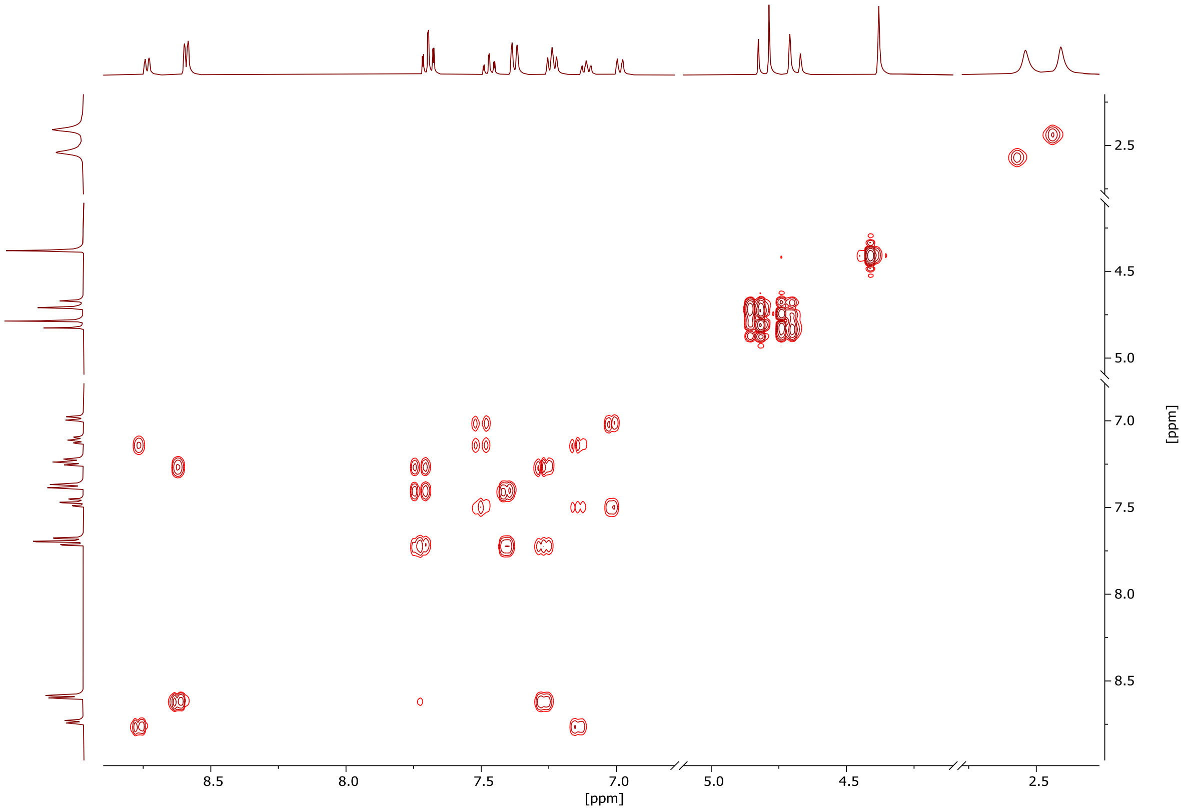

Figure S5. ^{1}H-^{1}H COSY NMR spectrum (MeCN-d$_3$) of complex **1(PF$_6$)$_2$**.

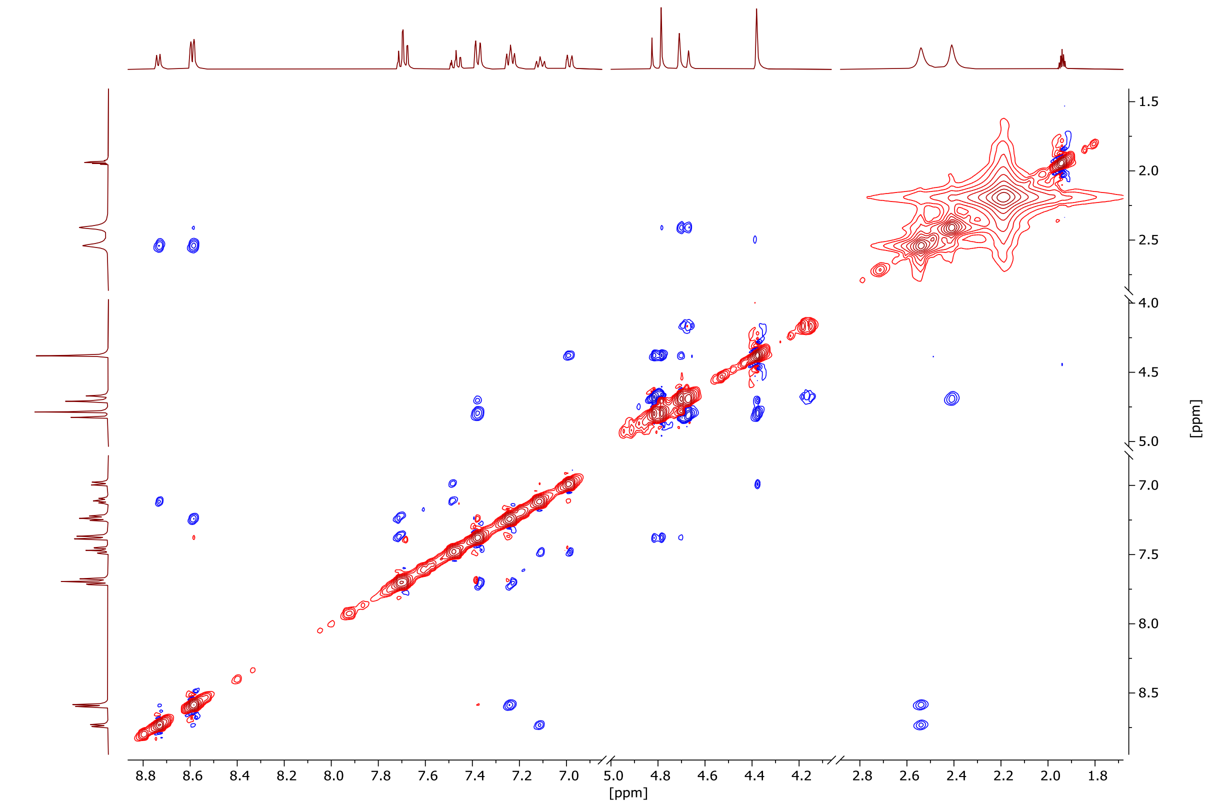

Figure S6. ^{1}H-^{1}H NOESY NMR spectrum (MeCN-d_3) of complex **1(PF$_6$)$_2$**.

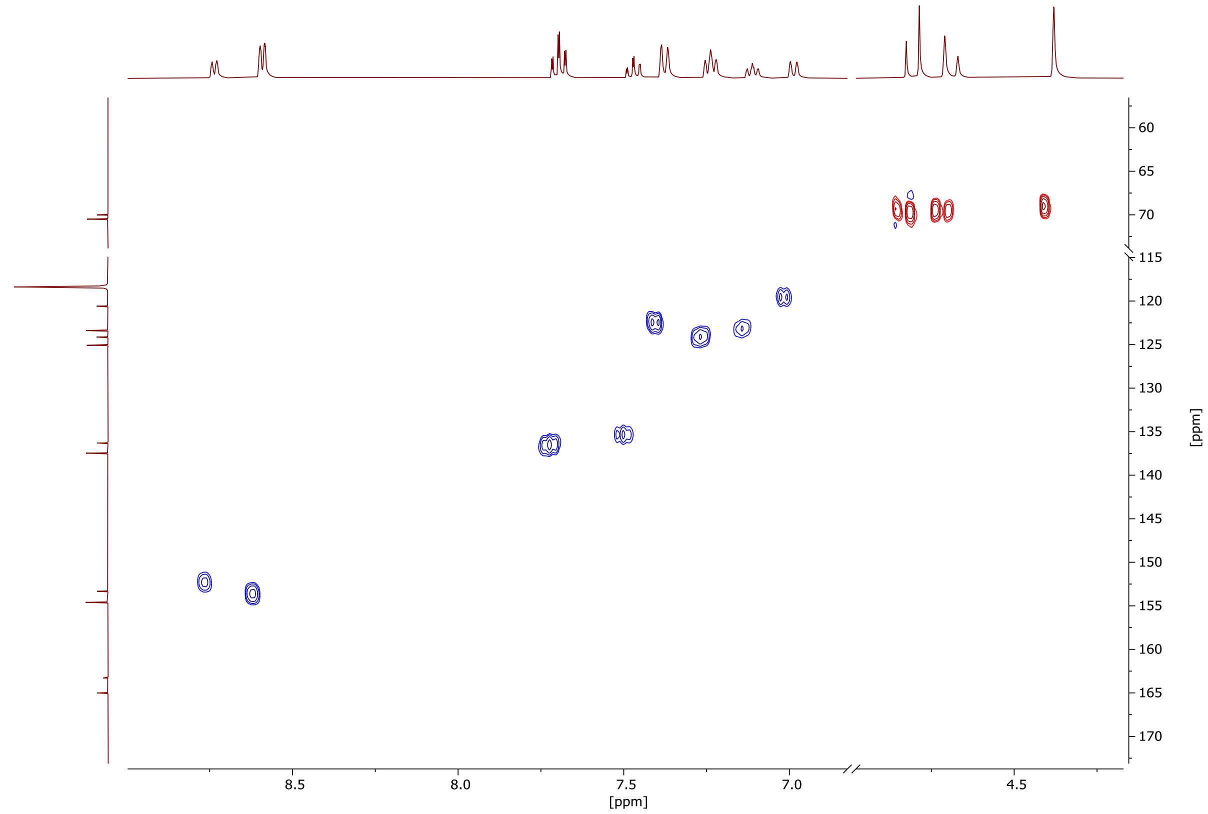

Figure S7. ^{1}H-^{13}C HSQC NMR spectrum (MeCN-d_3) of complex **1(PF$_6$)$_2$**.

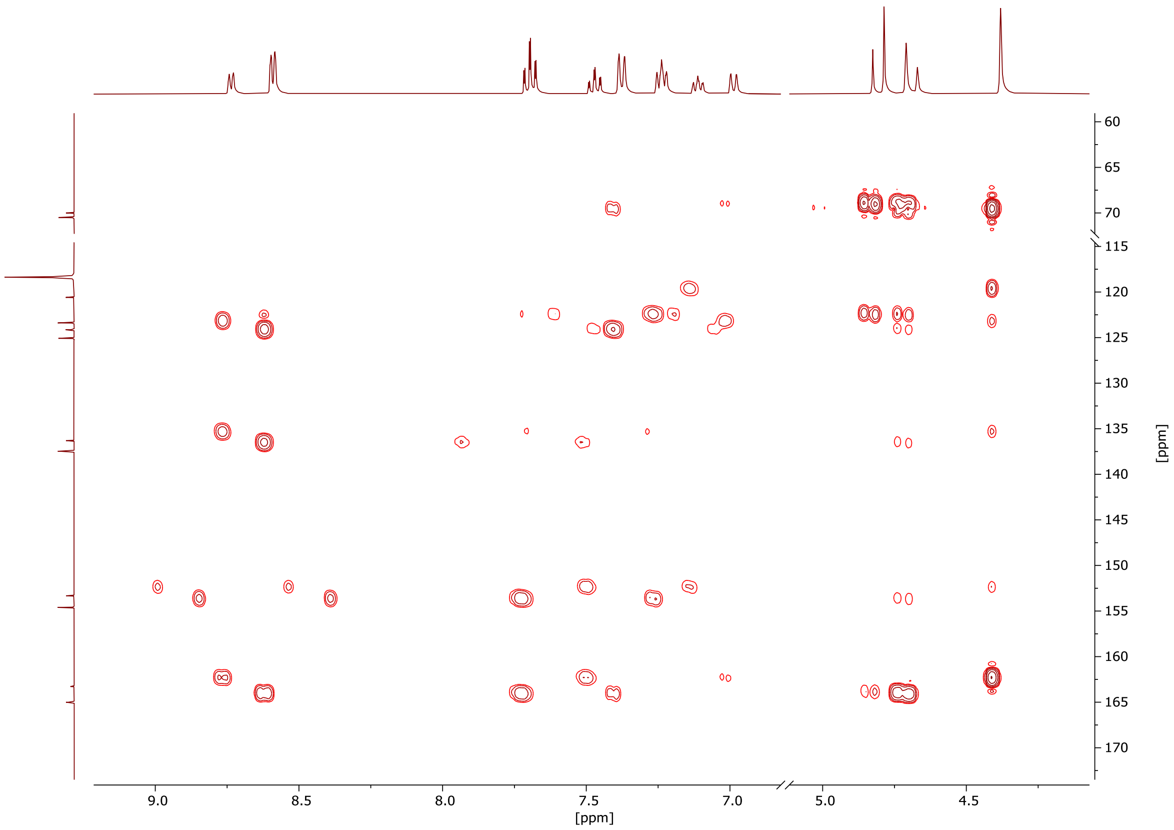

Figure S8. ^{1}H-^{13}C HMBC NMR spectrum (MeCN-d_3) of complex **1(PF$_6$)$_2$**.

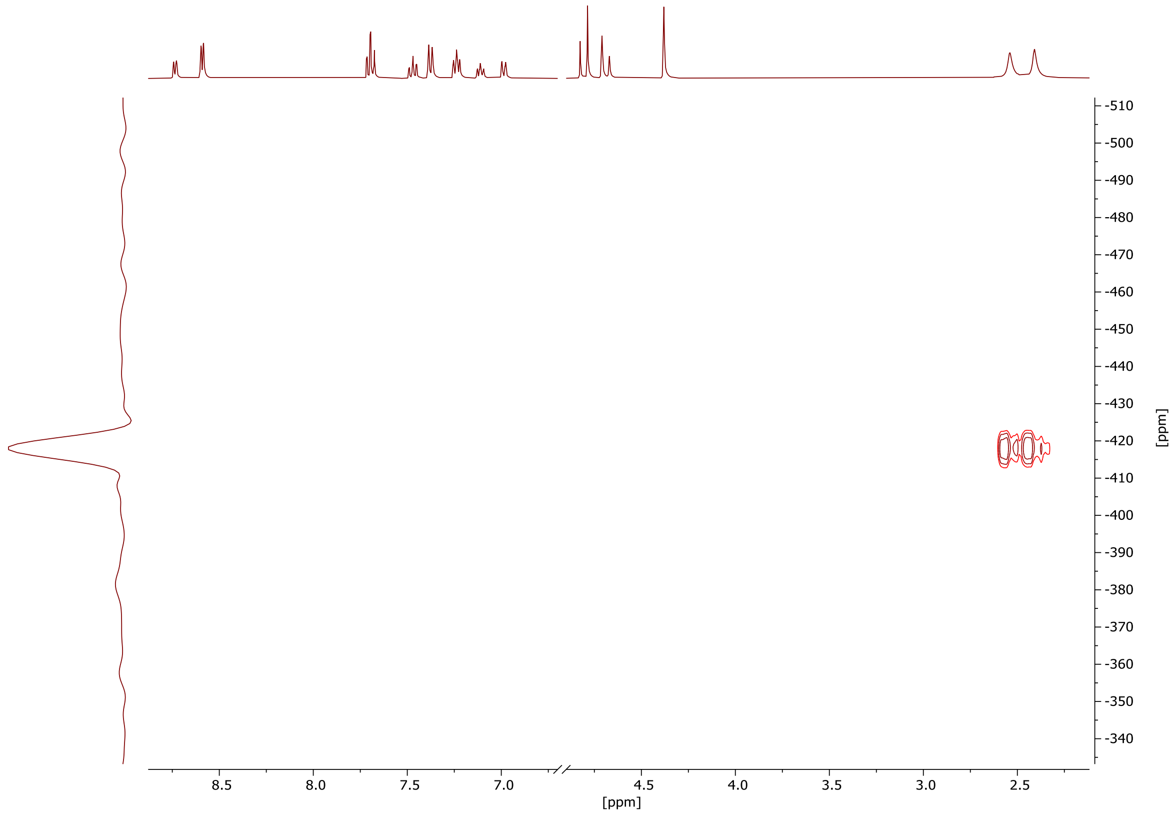

Figure S9. ^{1}H-^{15}N HSQC NMR spectrum (MeCN-d_3) of complex **1(PF$_6$)$_2$**.

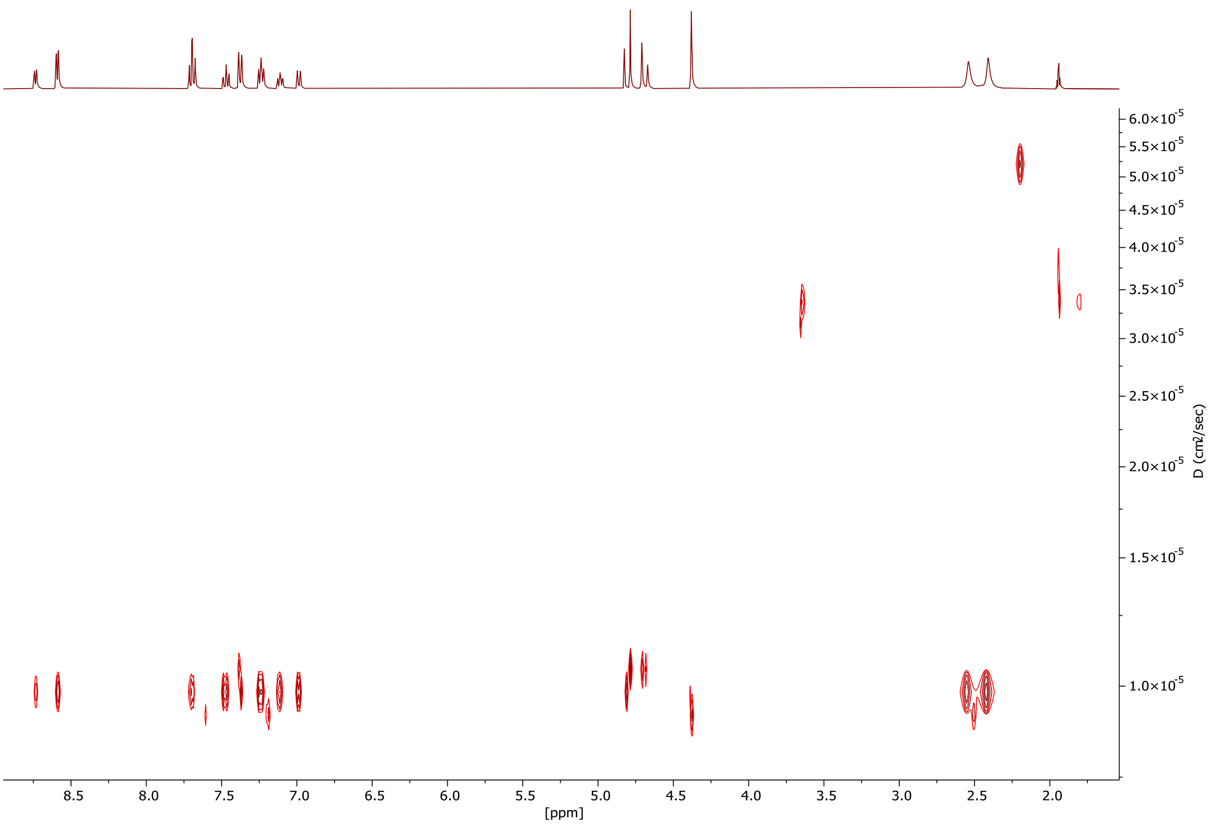

Figure S10. ^{1}H-DOSY NMR spectrum (600 MHz, MeCN-d$_3$) of complex **1(PF$_6$)$_2$**.

ESI-Mass spectrum.

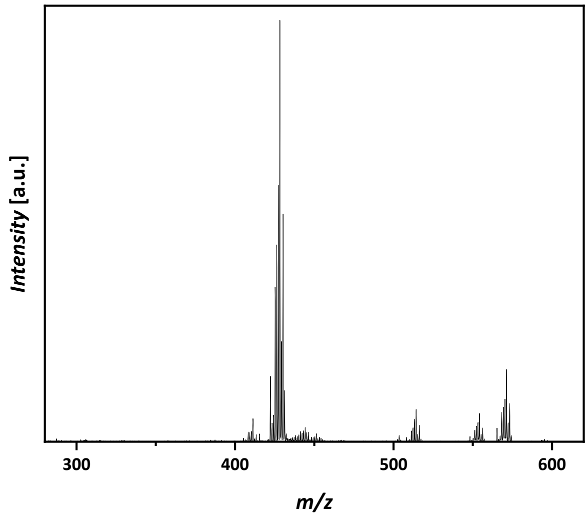

Figure S11. ESI(+)-MS spectrum of **1(PF$_6$)$_2$** in MeOH from *m/z* = 280 to 620.

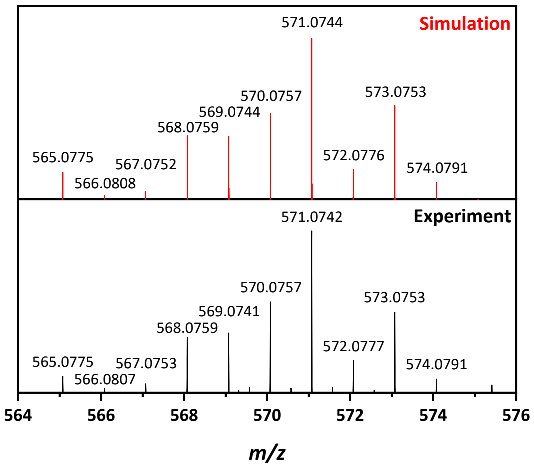

Figure S12. HR-ESI(+)-MS spectrum of **1(PF$_6$)$_2$** in MeOH from *m/z* = 564 to 576 (black) and simulation for [M – PF$_6$]$^+$ (red).

UV-Vis spectrum.

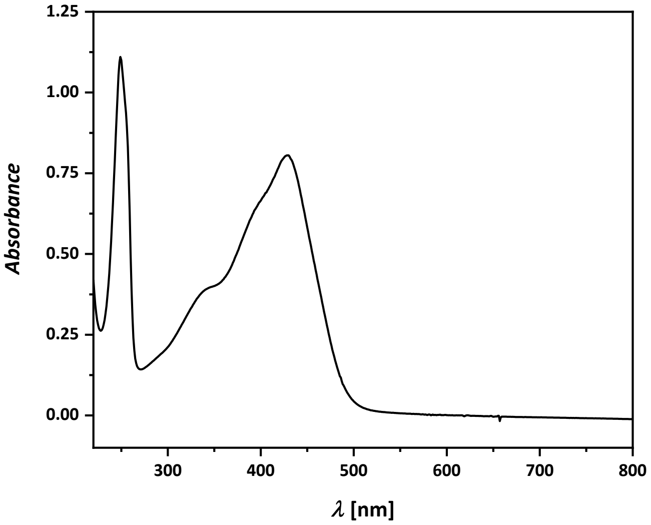

Figure S13. UV-Vis spectrum of complex **1(PF$_6$)$_2$** in MeCN at room temperature.

2.2 Complex [Ru(TPA)($^{15}NH_3$)$_2$](PF_6)$_2$ (^{15N}1(PF_6)$_2$)

Synthesis.

The synthetic procedure for the preparation of complex **^{15N}1(PF_6)$_2$** is identical to that of complex **1(PF_6)$_2$** (chapter 2.1), but using an aqueous solution of $^{15}NH_3$ (ca. 6 M).

^{1}H NMR (400 MHz, MeCN-d$_3$): δ = 8.74 (d, $^3J_{HH}$ = 5.3 Hz, 1 H, 12-H), 8.59 (d, $^3J_{HH}$ = 5.6 Hz, 2 H, 7-H), 7.70 (td, $^3J_{HH}$ = 7.8 Hz, $^4J_{HH}$ = 1.5 Hz, 2 H, 5-H), 7.47 (td, $^3J_{HH}$ = 7.8 Hz, $^4J_{HH}$ = 1.5 Hz, 1 H, 10-H), 7.38 (d, $^3J_{HH}$ = 6.5 Hz, 2 H, 4-H), 7.24 (t, $^3J_{HH}$ = 6.4 Hz, 2 H, 6-H), 7.11 (t, $^3J_{HH}$ = 5.9 Hz, 1 H, 11-H), 6.99 (d, $^3J_{HH}$ = 7.2 Hz, 1 H, 9-H), 4.81 (d, $^2J_{HH}$ = 16 Hz, 2 H, 1-H), 4.69 (d, $^2J_{HH}$ = 16 Hz, 2 H, 1'-H), 4.38 (s, 2 H, 2-H), 2.59 (d, $^1J_{NH}$ = 50.3 Hz, 3 H, NH_3), 2.42 (d, $^1J_{NH}$ = 51.5 Hz, 3H, NH_3) ppm.

ESI-MS (ESI(+), MeOH): *m/z* = 573.1 [M – PF_6]$^+$, 555.1 [M – $^{15}NH_3$ – PF_6]$^+$, 514.1 [*not identified*], 429.1 [M – $^{15}NH_3$ – 2 PF_6 + F*]$^+$. (** Fluoride likely originates from partial PF_6 hydrolysis in MeOH*)

NMR spectrum.

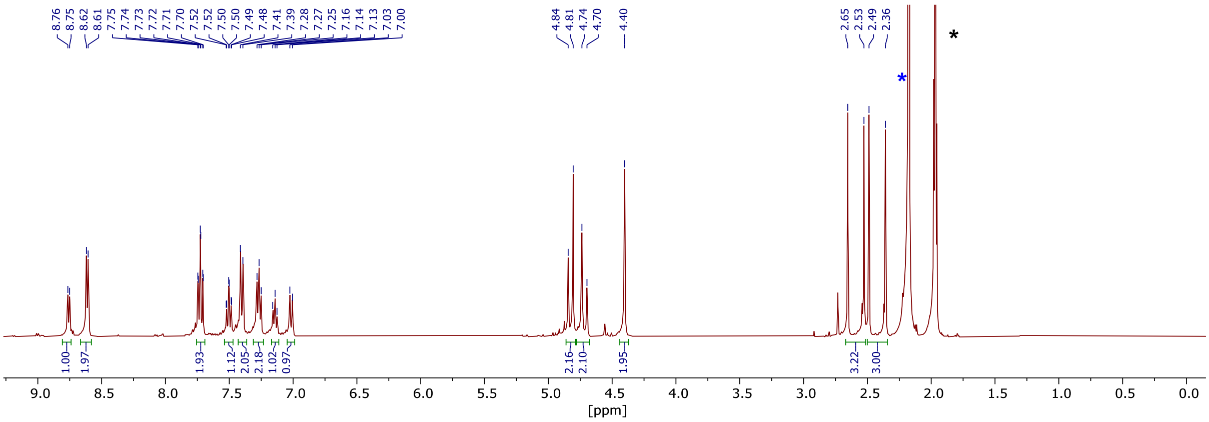

Figure S14. ^{1}H NMR spectrum (400 MHz, MeCN-d$_3$) of complex **^{15N}1(PF_6)$_2$**. Solvent signals are marked with an asterisk (black for MeCN, blue for water).

ESI-Mass spectrum.

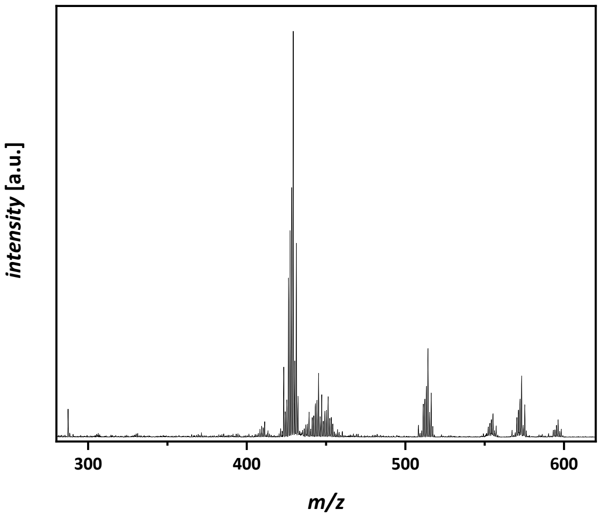

Figure S15. ESI(+)-MS spectrum of **1(PF_6)$_2$** in MeOH from *m/z* = 280 to 620.

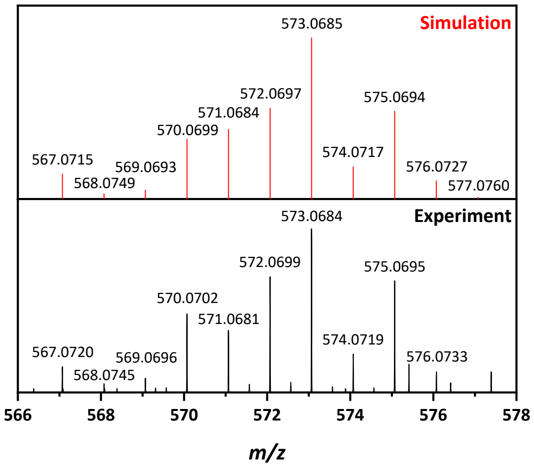

Figure S16. HR-ESI(+)-MS spectrum of ^{15N}**1(PF$_6$)$_2$** in MeOH from *m/z* = 566 to 578 (black) and simulation for $[M - PF_6]^+$ (red).

2.3 Complex [Ru(TPA)(MeCN)(NH$_3$)](PF$_6$)$_2$ (2(PF$_6$)$_2$)

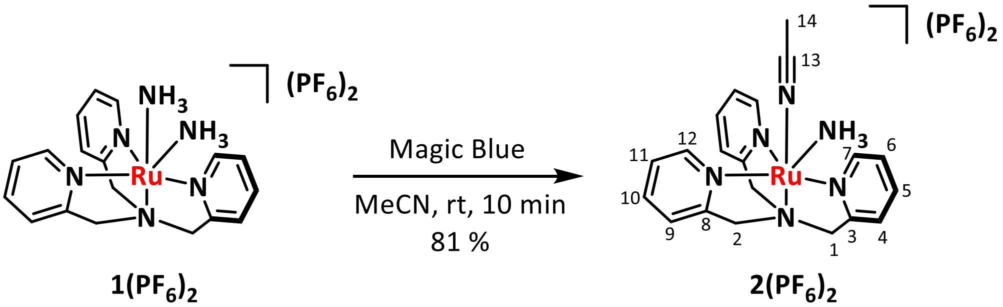

Synthesis.

Complex **1(PF$_6$)$_2$** (14.4 mg, 20.1 µmol, 1.00 equiv.) was dissolved in MeCN (1.5 mL) under N_2 atmosphere and mixed with a solution of Magic Blue (tris(4-bromophenyl)ammoniumyl hexachloroantimonate; 16.1 mg, 19.7 µmol, 0.98 equiv.) in MeCN (2.5 mL). The initially blue mixture turned greenish-yellow and was stirred for 10 min. After filtration, Et_2O (30 mL) was added to the filtrate to obtain a precipitate which was filtered off, washed with Et_2O and dried under reduced pressure to obtain **2(PF$_6$)$_2$** as a yellow solid (12.1 mg, 16.3 µmol, 81 %). Yellow block-shaped crystals suitable for X-ray analysis were grown by slow diffusion of Et_2O into a solution of **2(PF$_6$)$_2$** in MeCN at 5 °C.

^{1}H NMR (400 MHz, MeCN-d$_3$): δ = 8.98 (d, $^3J_{HH}$ = 5.1 Hz, 1 H, 12-H), 8.70 (d, $^3J_{HH}$ = 5.6 Hz, 2 H, 7-H), 7.74 (td, $^3J_{HH}$ = 7.8 Hz, $^4J_{HH}$ = 1.5 Hz, 2 H, 5-H), 7.52 (td, $^3J_{HH}$ = 7.7 Hz, $^4J_{HH}$ = 1.5 Hz, 1 H, 10-H), 7.41 (d, $^3J_{HH}$ = 6.5 Hz, 2 H, 4-H), 7.27 (t, $^3J_{HH}$ = 6.5 Hz, 2 H, 6-H), 7.16 (t, $^3J_{HH}$ = 6.7 Hz, 1 H, 11-H), 7.02 (d, $^3J_{HH}$ = 9.4 Hz, 1 H, 9-H), 4.87 (d, $^2J_{HH}$ = 16 Hz, 2 H, 1-H), 4.80 (d, $^2J_{HH}$ = 16 Hz, 2 H, 1'-H), 4.53 (s, 2 H, 2-H), 2.71 (s, 3 H, 14-H), 2.46 (s, 3H, NH$_3$) ppm.

^{13}C NMR (101 MHz, MeCN-d$_3$): δ = 163.9 (s, 3-C), 162.1 (s, 8-C), 154.5 (s, 7-C), 153.4 (s, 12-C), 138.1 (s, 5-C), 137.0 (s, 10-C), 125.5 (s, 6-C), 124.6 (s, 11-C), 123.6 (s, 4-C), 120.7 (s, 9-C), 70.2 (s, 1-C), 70.1 (s, 2-C), 5.23 (s, 14-C).

^{31}P NMR (203 MHz, MeCN-d$_3$): δ = −144.6 (sept, $^1J_{PF}$ = 707 Hz).

^{19}**F NMR** (471 MHz, MeCN-d$_3$): δ = −72.9 (d, $^{1}J_{FP}$ = 706 Hz).
ESI-MS (ESI(+), MeOH): m/z = 595.1 [M – PF$_6$]$^+$, 444.1 [M – MeCN – 2 PF$_6$ + Cl]$^+$, 427.1 [M – NH$_3$ – MeCN – 2 PF$_6$ + Cl]$^+$.
UV-Vis (MeCN): λ_{max} = 248, 400 nm.

NMR spectra.

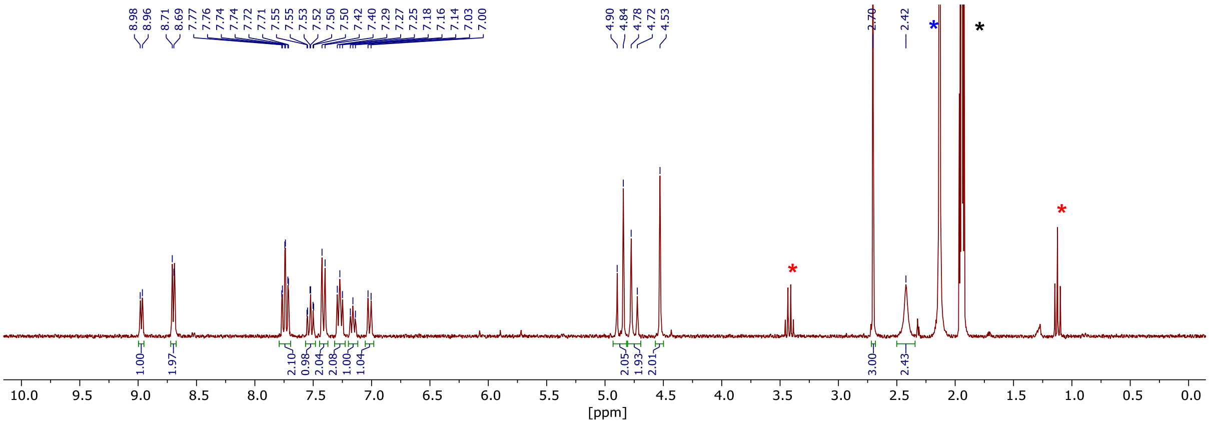

Figure S17. ^{1}H NMR spectrum (300 MHz, MeCN-d$_3$) of complex **2(PF$_6$)$_2$**. Solvent signals are marked with an asterisk (black for MeCN, red for diethylether, blue for water).

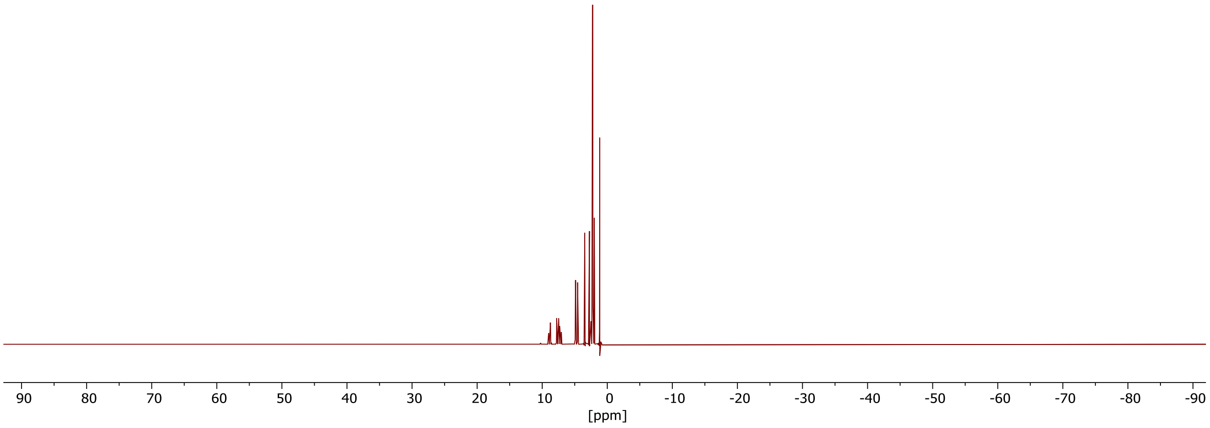

Figure S18. ^{1}H-PARA NMR spectrum (400 MHz, MeCN-d$_3$) of complex **2(PF$_6$)$_2$**.

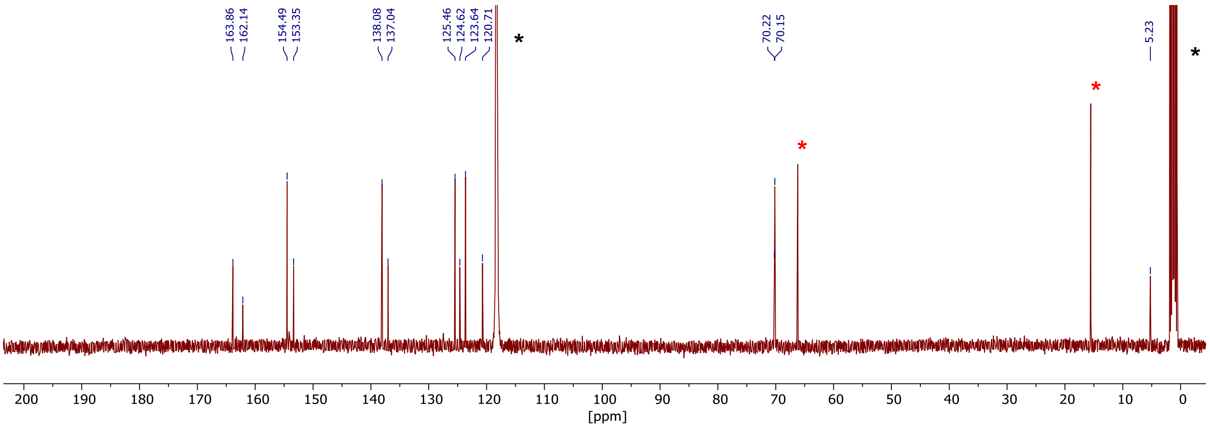

Figure S19. ^{13}C NMR spectrum (101 MHz, MeCN-d$_3$) of complex **2(PF$_6$)$_2$**. Solvent signals are marked with an asterisk (black for MeCN, red for diethylether).

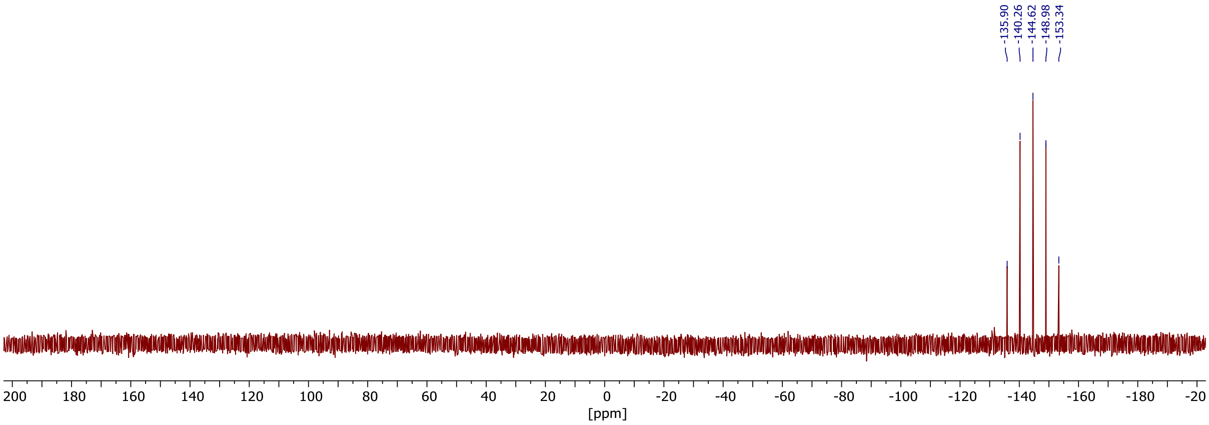

Figure S20. ^{31}P NMR spectrum (162 MHz, MeCN-d_3) of complex **2(PF$_6$)$_2$**.

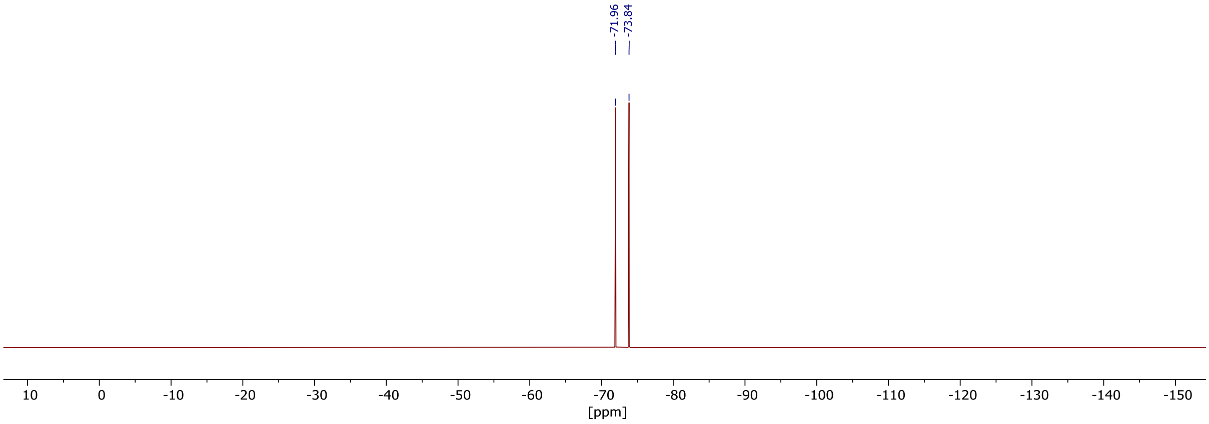

Figure S21. ^{19}F NMR spectrum (376 MHz, MeCN-d_3) of complex **2(PF$_6$)$_2$**.

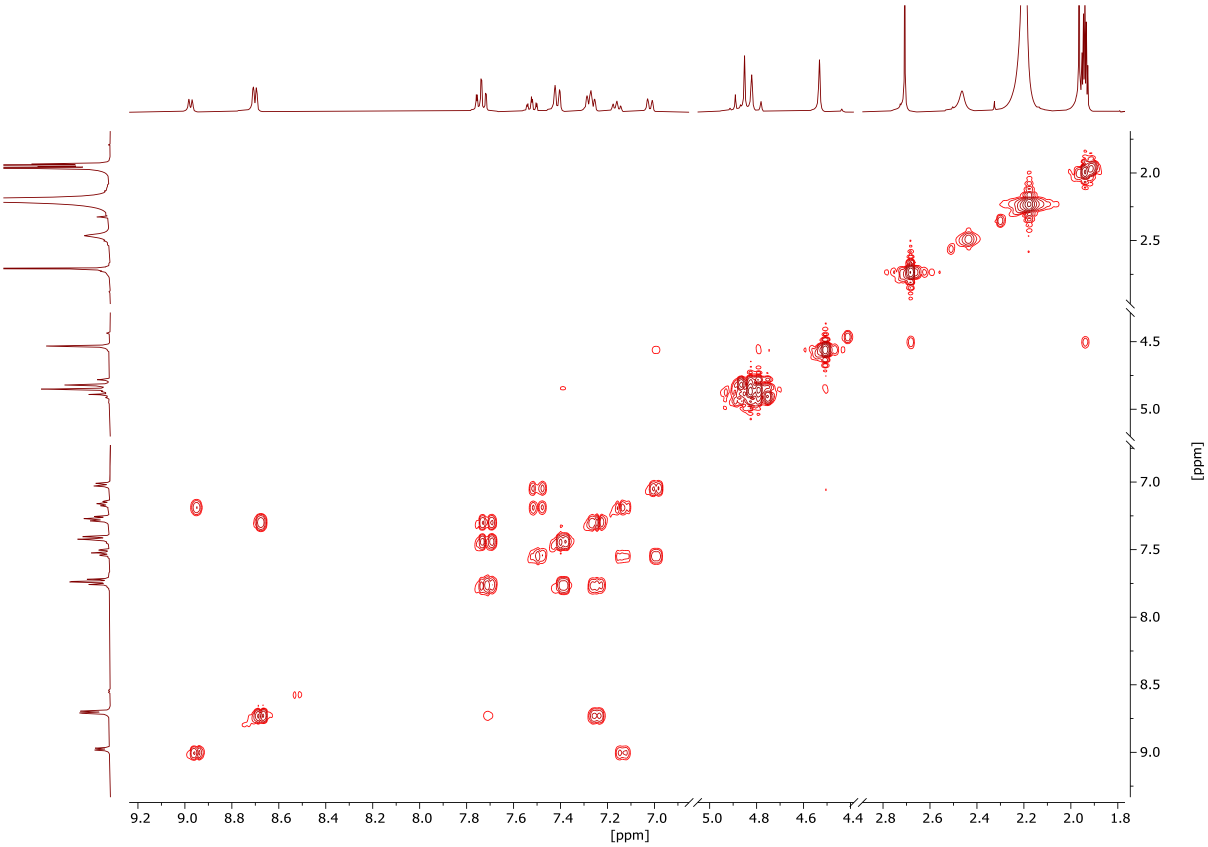

Figure S22. ^{1}H-^{1}H COSY NMR spectrum (MeCN-d_3) of complex **2(PF$_6$)$_2$**.

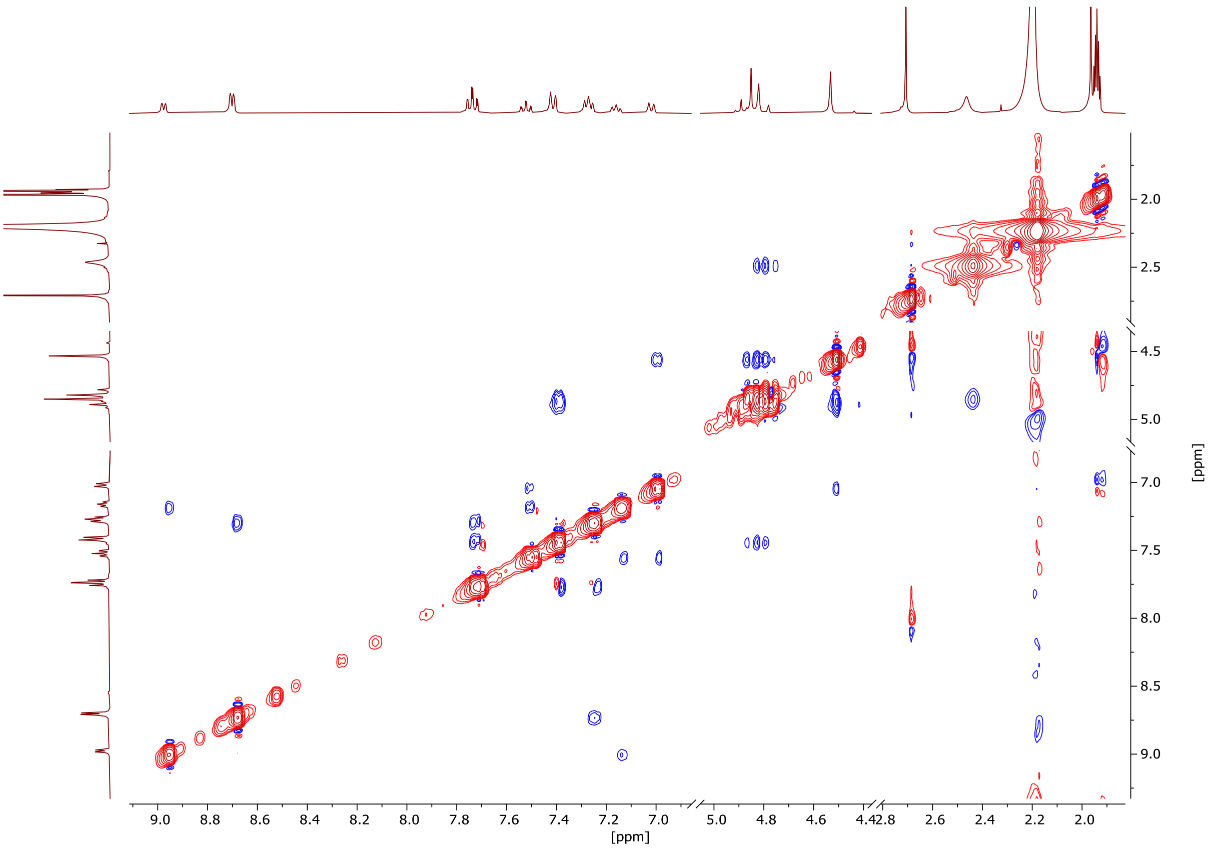

Figure S23. ^{1}H-^{1}H NOESY NMR spectrum (MeCN-d$_3$) of complex **2(PF$_6$)$_2$**.

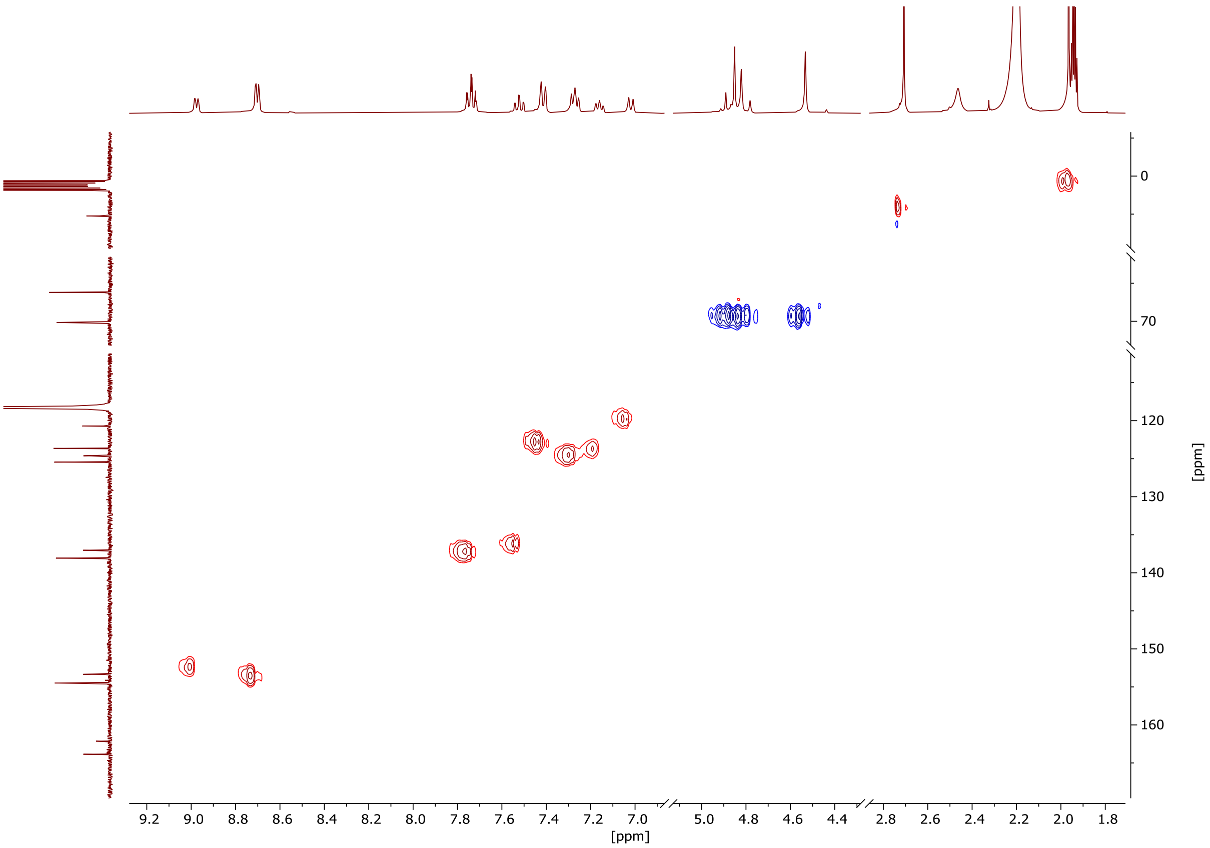

Figure S24. ^{1}H-^{13}C HSQC NMR spectrum (MeCN-d$_3$) of complex **2(PF$_6$)$_2$**.

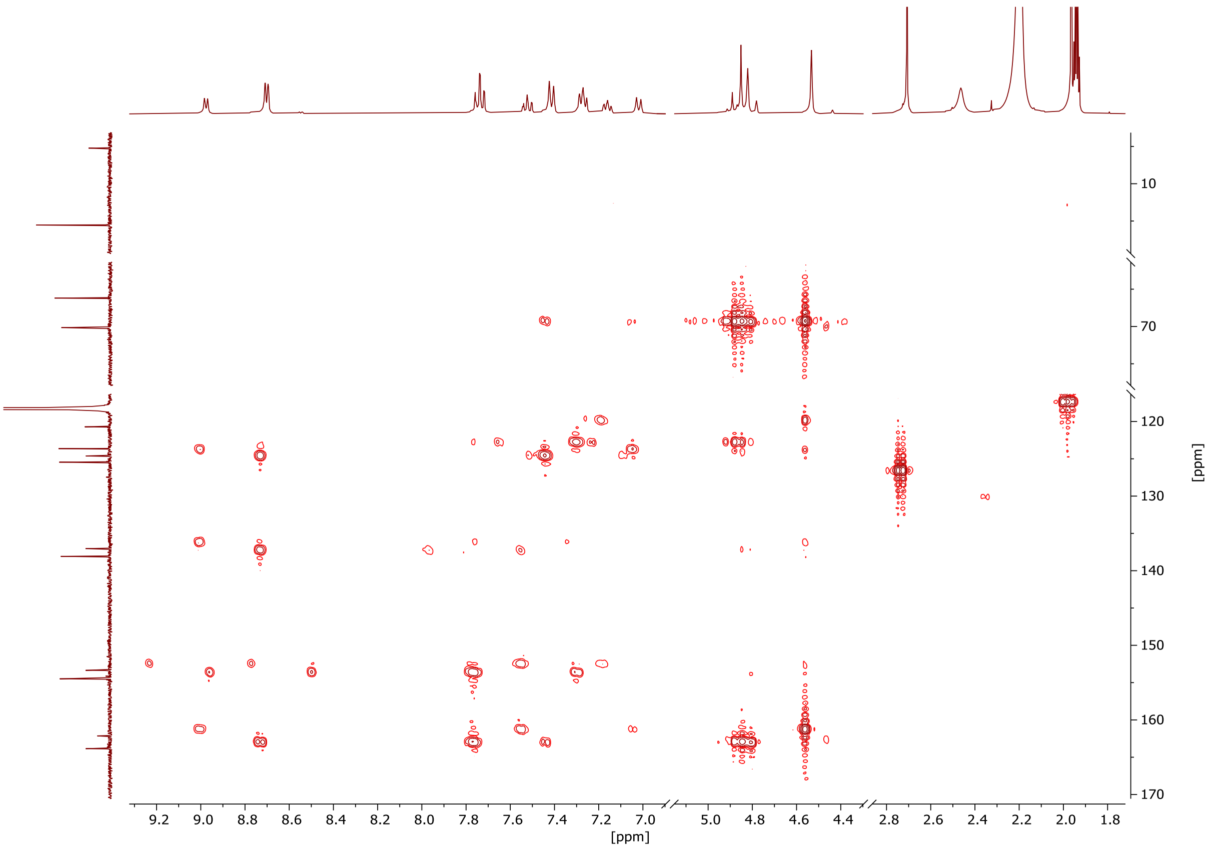

Figure S25. ^{1}H-^{13}C HMBC NMR spectrum (MeCN-d_3) of complex **2(PF$_6$)$_2$.**

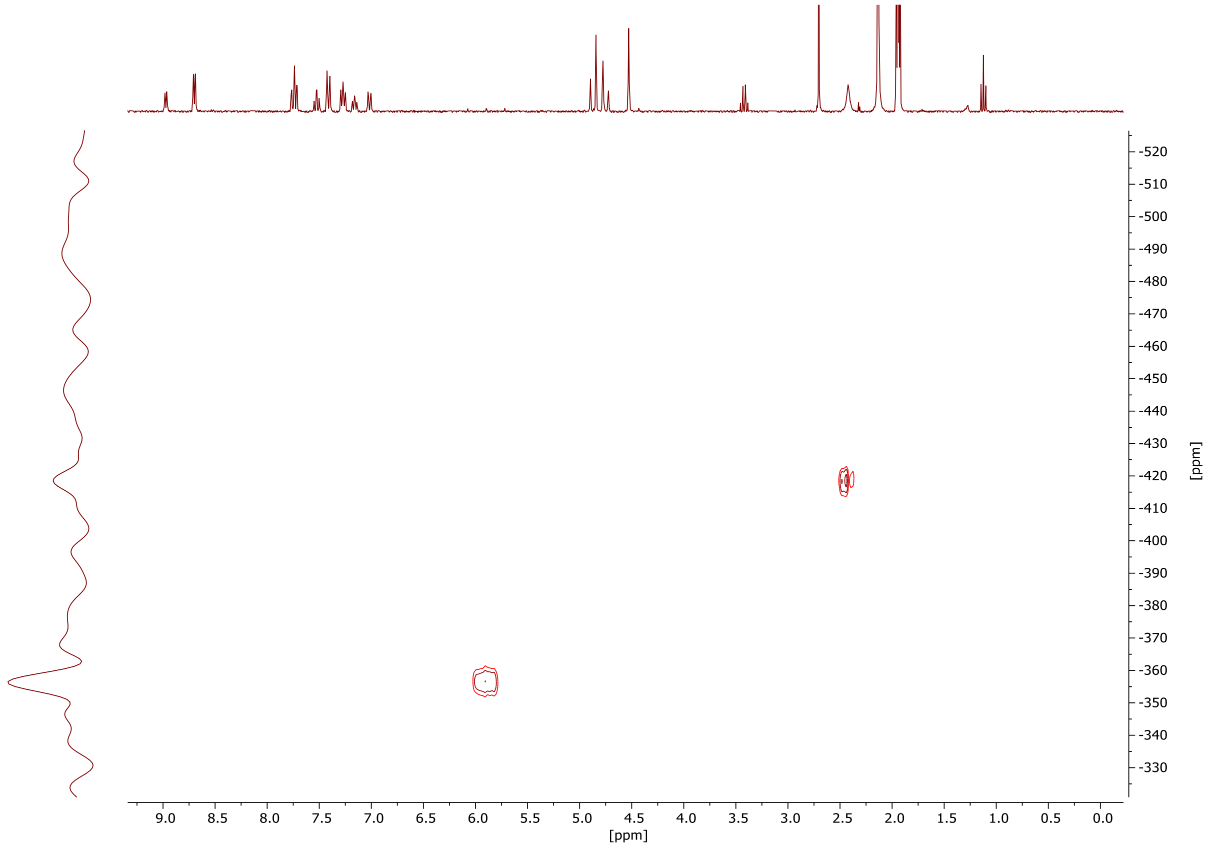

Figure S26. ^{1}H-^{15}N HSQC NMR spectrum (MeCN-d_3) of complex **2(PF$_6$)$_2$**.

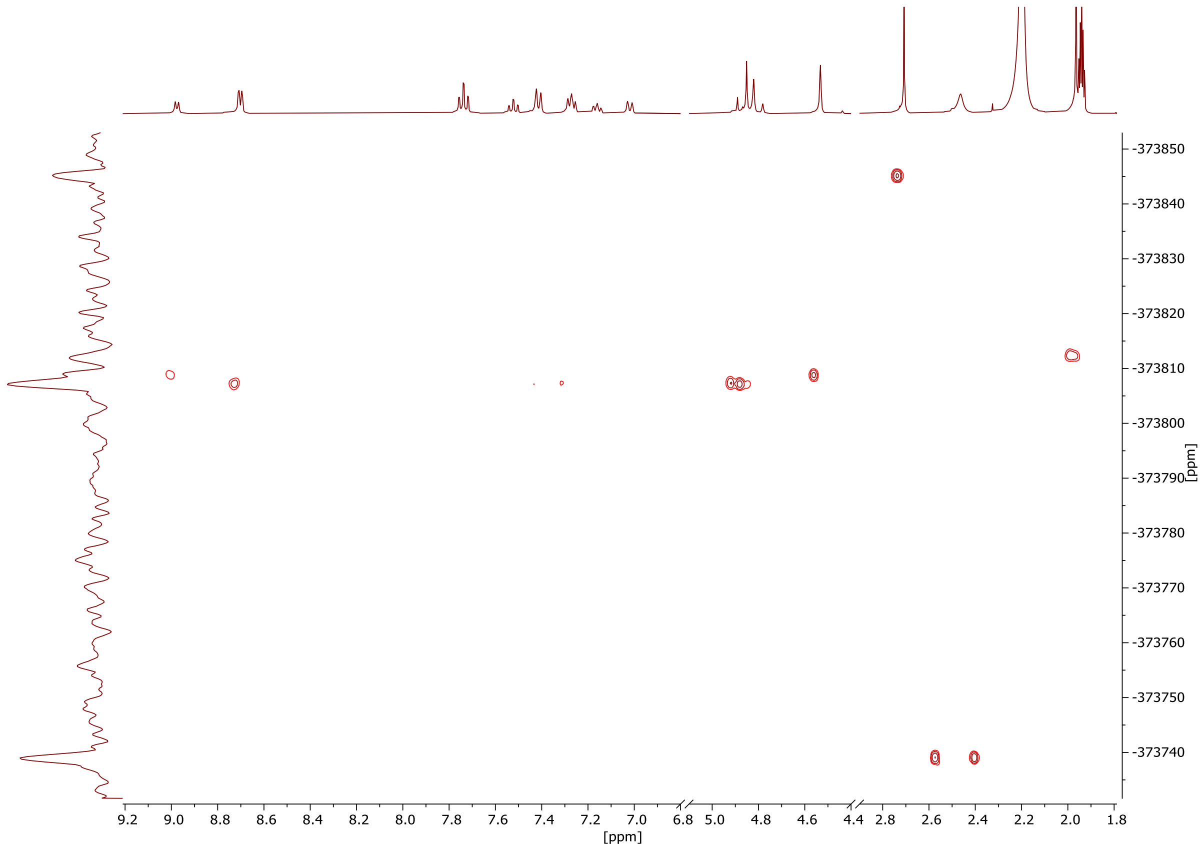

Figure S27. ^{1}H-^{15}N HMBC NMR spectrum (MeCN-d_3) of complex **2(PF$_6$)$_2$**.

ESI-Mass spectrum.

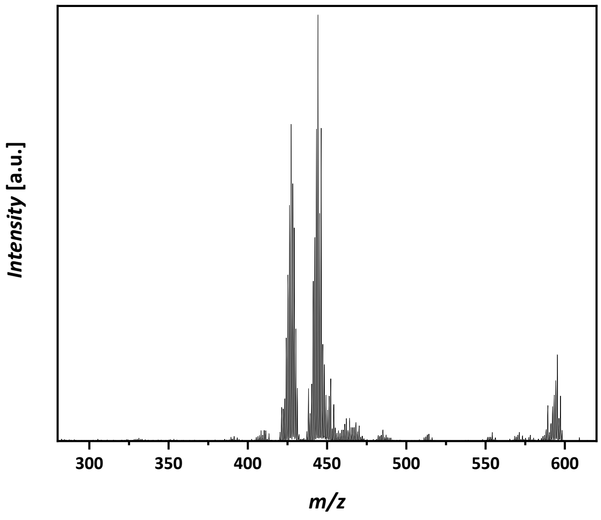

Figure S28. ESI(+)-MS spectrum of **2(PF$_6$)$_2$** in MeOH from *m/z* = 280 to 620.

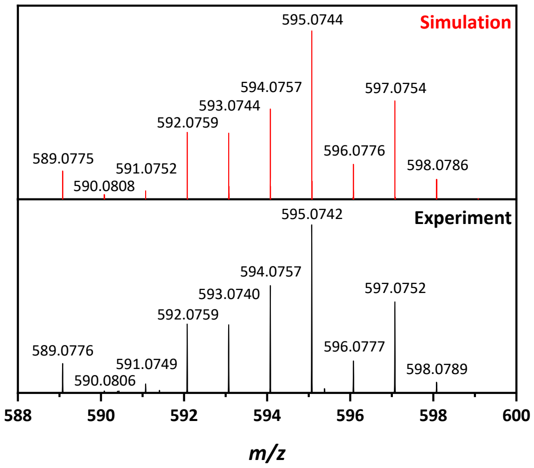

Figure S29. HR-ESI(+)-MS spectrum of **2(PF$_6$)$_2$** in MeOH from *m/z* = 588 to 600 (black) and simulation for [M – PF$_6$]$^+$ (red).

UV-Vis spectrum.

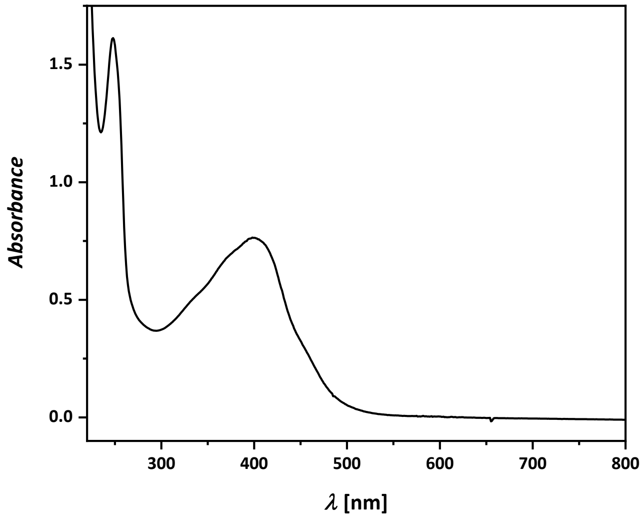

Figure S30. UV-Vis spectrum of complex **2(PF$_6$)$_2$** in MeCN at room temperature.

2.4 Complex $[Ru(TPA)(N_2H_4)_2](BPh_4)_2$ ($3(BPh_4)_2$)

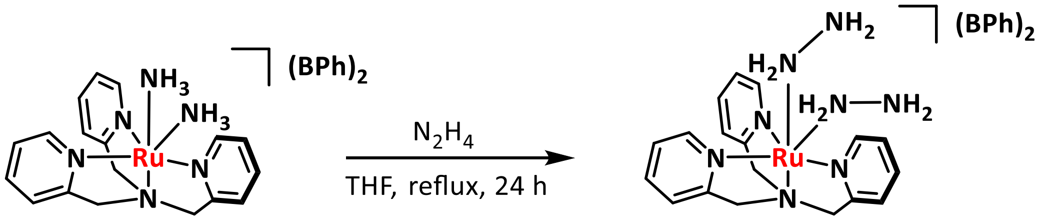

1(BPh$_4$)$_2$ **3(BPh)$_2$**

Synthesis.

Complex **1(PF$_6$)$_2$** (150 mg, 1.21 mmol, 1.00 equiv.) was suspended in THF (25 mL). Upon addition of $NaBPh_4$ (171 mg, 0.50 mmol, 2.4 equiv.), the solid dissolved forming a yellow solution, thus indicating the solubilization of **1**$^{2+}$ upon anion exchange. The solution was filtered and the solvent was removed under reduced pressure. The obtained yellow solid was used without further purification and dissolved in a solution of N_2H_4 in THF (1 M, 10 mL) under N_2 atmosphere. The mixture was heated to reflux for 24 h. After cooling to room temperature, pentane (20 mL) was added. The precipitate was filtered off, washed with Et_2O and pentane and dried under reduced pressure to obtain **3(BPh$_4$)$_2$** as a yellow solid (yield not determined, product still contains impurities of $NaBPh_4$). Yellow block-shaped crystals suitable for X-ray analysis were grown by layering a solution of **3(BPh$_4$)$_2$** in MeCN with Et_2O at room temperature under N_2 atmosphere.

^{1}H NMR (400 MHz, MeCN-d$_3$): δ = 8.81-8.69 (m, 3 H), 7.67 (td, $^3J_{HH}$ = 7.8 Hz, $^4J_{HH}$ = 1.5 Hz, 2 H), 7.45 (td, $^3J_{HH}$ = 7.6 Hz, $^4J_{HH}$ = 1.5 Hz, 1 H), 7.32 (d, $^3J_{HH}$ = 8.3 Hz,*), 7.30-7.25 (m, BPh_4), 7.21 (t, $^3J_{HH}$ = 6.7 Hz, 2 H), 7.12 (t, $^3J_{HH}$ = 5.9 Hz, 1 H), 7.04-6.96 (t, BPh_4), 6.92 (d, $^3J_{HH}$ = 7.9 Hz, 1 H), 6.88-6.80 (t, BPh_4), 5.64 (s, 2 H, NH_2), 5.41 (s, 2 H, NH_2), 4.84 (d, $^2J_{HH}$ = 16 Hz, 2 H), 4.65 (d, $^2J_{HH}$ = 16 Hz, 2 H), 4.24 (s, 2 H), 3.76 (s, 2 H, NH_2), 3.13 (s, 2 H, NH_2) ppm.

*integration of signal not possible due to overlap with BPh_4^{2-} signal

NMR spectrum.

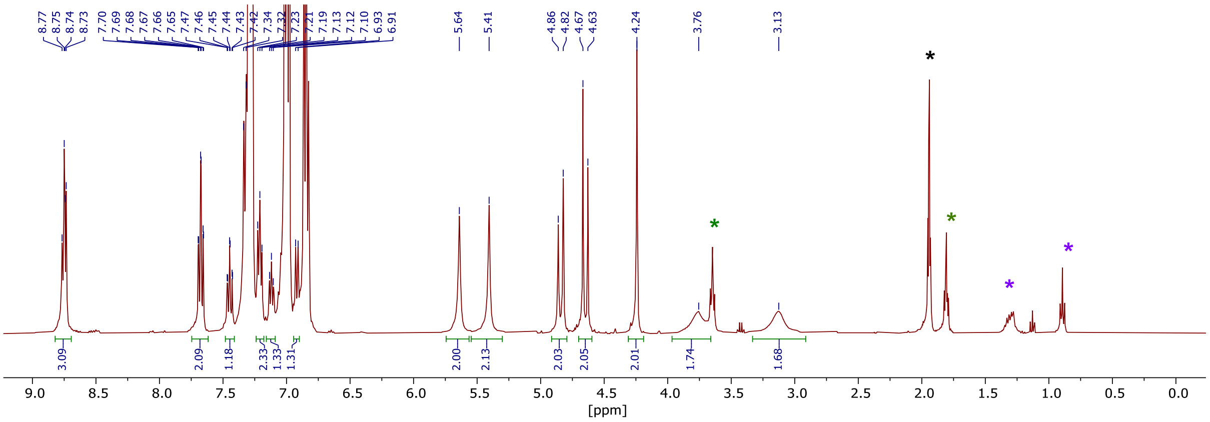

Figure S31. ^{1}H NMR spectrum (400 MHz, MeCN-d$_3$) of complex **3(BPh$_4$)$_2$**. Solvent signals are marked with an asterisk (black for MeCN, green for THF, purple for pentane).

2.5 Complex $[Ru(TPA)(MeCN)_2](PF_6)_2$ ($XI(PF_6)_2$)

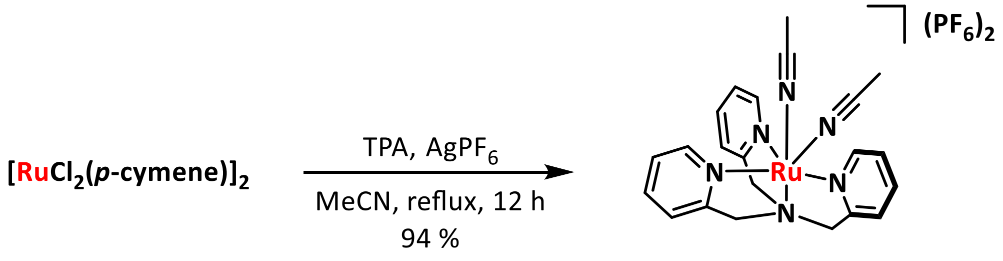

XI(PF$_6$)$_2$

Synthesis.

Complex **XI(PF$_6$)$_2$** was synthesized according to a modified literature procedure by Britovsek and co-workers.[3]

$[RuCl_2(p\text{-cymene})]_2$ (320 mg, 0.523 mmol, 0.50 equiv.),[2] TPA (300 mg, 1.04 mmol, 1.00 equiv.) and $AgPF_6$ (528 mg, 2.09 mmol, 2.00 equiv.) were dissolved in MeCN (25 mL) und N_2 atmosphere. The mixture was heated to reflux for 12 h under the exclusion of light. After cooling to room temperature, the reaction mixture was filtered and the solvent of the filtrate was removed under reduced pressure. The solid residue was redissolved in DCM (10 mL) and precipitated by addition of pentane (25 mL). This step was repeated two times and the resulting solid was dried under reduced pressure to yield **XI(PF$_6$)$_2$** as a yellow powder (720 mg, 0.943 mmol, 94 %).

^{1}H NMR (400 MHz, MeCN-d$_3$): δ = 8.96 (d, $^3J_{HH}$ = 6.5 Hz, 1 H), 8.67 (d, $^3J_{HH}$ = 5.6 Hz, 2 H), 7.80 (td, $^3J_{HH}$ = 7.8 Hz, $^4J_{HH}$ = 1.6 Hz, 2 H), 7.60 (td, $^3J_{HH}$ = 7.8 Hz, $^4J_{HH}$ = 1.5 Hz, 1 H), 7.47 (d, $^3J_{HH}$ = 7.3 Hz, 2 H), 7.32 (t, $^3J_{HH}$ = 6.4 Hz, 2 H), 7.23 (t, $^3J_{HH}$ = 6.7 Hz, 1 H), 7.04 (d, $^3J_{HH}$ = 7.2 Hz, 1 H), 4.97 (d, $^2J_{HH}$ = 15 Hz, 2 H), 4.88 (d, $^2J_{HH}$ = 15 Hz, 2 H), 4.56 (s, 3 H), 2.72 (s, 3 H, CH_3CN), 2.31 (s, 3H, CH_3CN) ppm.

UV-Vis (MeCN): λ_{max} = 247, 318, 372 nm.

NMR spectrum.

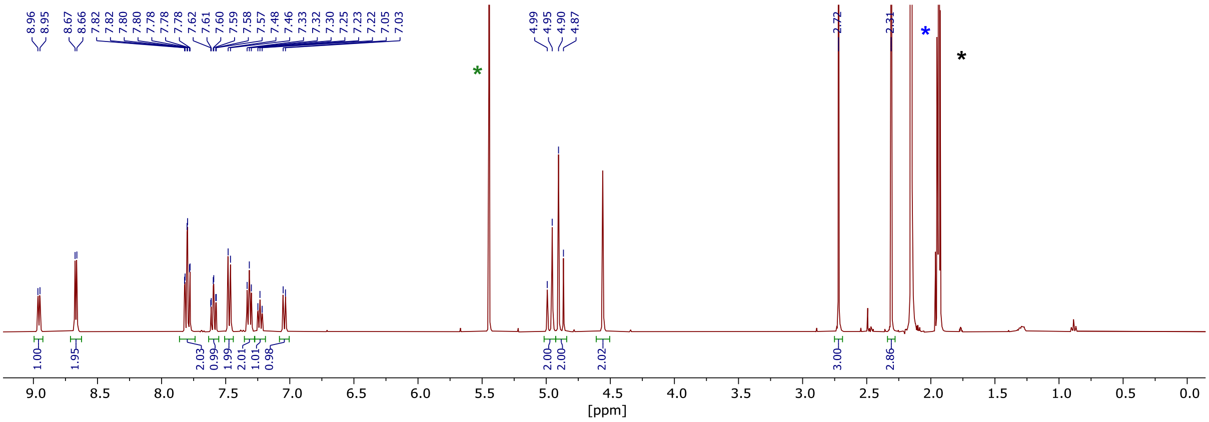

Figure S32. ^{1}H NMR spectrum (400 MHz, MeCN-d$_3$) of complex **XI(PF$_6$)$_2$**. Solvent signals are marked with an asterisk (black for MeCN, green for DCM, blue for water).

UV-Vis spectrum.

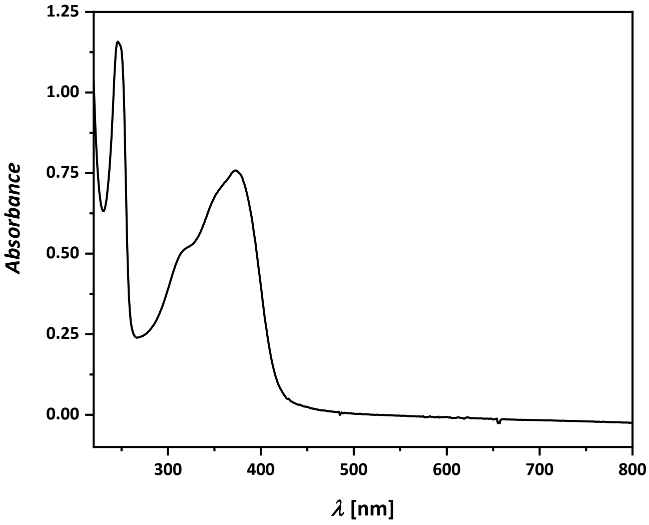

Figure S33. UV-Vis spectrum of complex **XI(PF_6)$_2$** in MeCN at room temperature.

3. Crystallographic Data

Crystal data and details of the data collections for complexes **1(PF_6)$_2$**, **2(PF_6)$_2$**, and **3(BPh_4)$_2$** are given in Table S1, selected bond lengths in Table S2. The molecular structures are shown in Figure S34, Figure S35 and Figure S36. Non–hydrogen atoms were refined anisotropically. Hydrogen atoms were placed in calculated positions and assigned to an isotropic displacement parameter of 1.5/1.2 U_{eq}(C) and 1.5 U_{eq}(N) in case of **1(PF_6)$_2$**. In case of **2(PF_6)$_2$**, the nitrogen bound hydrogen atoms were refined freely. In the case of **3(BPh_4)$_2$**, DFIX restraints (d(N–H) = 0.95 Å) were applied to the N–H bond lengths of the nitrogen-bound hydrogen atoms, which were otherwise refined freely. A PF_6^- counter anion was found to be disordered in **2(PF_6)$_2$** (occupancy factors: 0.618(10)/ 0.382(10)) and was refined using SAME restraints and EADP constraints.

CCDC 2545782-2545784 contain the supplementary crystallographic data for this paper. These data can be obtained free of charge via www.ccdc.cam.ac.uk/data_request/cif, or by emailing data_request@ccdc.cam.ac.uk, or by contacting The Cambridge Crystallographic Data Centre, 12 Union Road, Cambridge CB21EZ, UK; fax: +44 1223 336033.

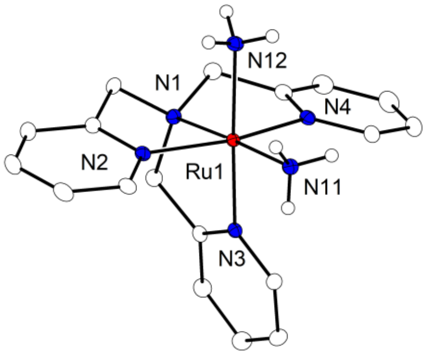

Figure S34. Plot (30% probability thermal ellipsoids) of the molecular structure of cationic part of **1**$(PF_6)_2$ (most hydrogen atoms omitted for clarity). Selected bond lengths [Å] and angles [°]: Ru1–N1 2.0739(15), Ru1–N2 2.0663(16), Ru1–N3 2.0565(15), Ru1–N4 2.0689(16), Ru1–N11 2.1599(16), Ru1–N12 2.1547(16); N3–Ru1–N2 95.30(6), N3–Ru1–N4 83.92(6), N2–Ru1–N4 164.08(6), N3–Ru1–N1 82.26(6), N2–Ru1–N1 81.23(6), N4–Ru1–N1 82.91(6), N3–Ru1–N12 176.94(6), N2–Ru1–N12 87.58(6), N4–Ru1–N12 93.03(6), N1–Ru1–N12 97.17(6), N3–Ru1–N11 97.25(6), N2–Ru1–N11 95.54(6), N4–Ru1–N11 100.34(6), N1–Ru1–N11 176.65(6), N12–Ru1–N11 83.49(7).

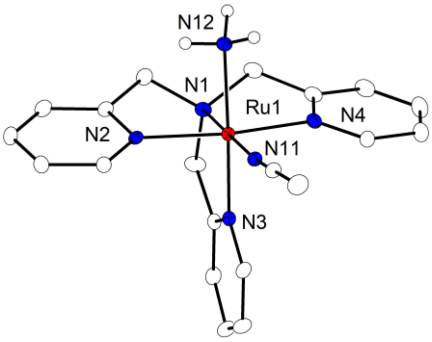

Figure S35. Plot (30% probability thermal ellipsoids) of the molecular structure of cationic part of **2**$(PF_6)_2$ (most hydrogen atoms omitted for clarity). Only one of the two crystallographically independent molecule is shown. Selected bond lengths [Å] and angles [°]: Ru1–N1 2.068(3), Ru1–N2 2.060(3), Ru1–N3 2.046(3), Ru1–N4 2.060(3), Ru1–N11 2.039(3), Ru1–N12 2.142(3); N11–Ru1–N3 96.23(12), N11–Ru1–N4 95.43(12), N3–Ru1–N4 88.73(12), N11–Ru1–N2 99.80(12), N3–Ru1–N2 91.03(12), N4–Ru1–N2 164.70(12), N11–Ru1–N1 178.35(13), N3–Ru1–N1 82.77(12), N4–Ru1–N1 83.25(12), N2–Ru1–N1 81.55(12), N11–Ru1–N12 85.93(13), N3–Ru1–N12 177.58(13), N4–Ru1–N12 92.16(13), N2–Ru1–N12 87.52(13), N1–Ru1–N12 95.10(13).

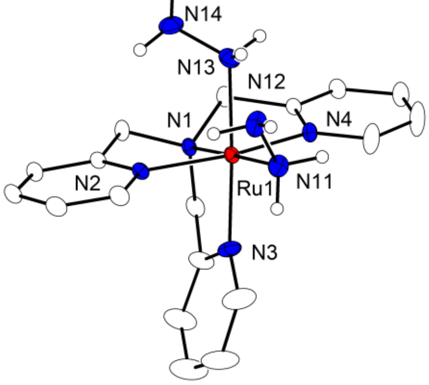

Figure S36. Plot (30% probability thermal ellipsoids) of the molecular structure of cationic part of **3**$(BPh_4)_2$ (most hydrogen atoms omitted for clarity). Selected bond lengths [Å] and angles [°]: Ru1–N1 2.0703(18), Ru1–N2 2.0760(19), Ru1–N3 2.077(2), Ru1–N4 2.0747(19), Ru1–N11 2.157(2), Ru1–N13 2.129(2); N1–Ru1–N4 81.04(7), N1–Ru1–N2 82.59(7), N4–Ru1–N2 163.43(7), N1–Ru1–N3 81.19(8), N4–Ru1–N3 96.21(8), N2–Ru1–N3 83.79(8), N1–Ru1–N13 96.86(8), N4–Ru1–N13 85.40(8), N2–Ru1–N13 94.02(8), N3–Ru1–N13 177.23(9), N1–Ru1–N11 177.87(8), N4–Ru1–N11 97.88(8), N2–Ru1–N11 98.57(8), N3–Ru1–N11 97.13(9), N13–Ru1–N11 84.87(9).

Table S1. Crystal data and refinement details for complexes **1(PF$_6$)$_2$**, **2(PF$_6$)$_2$**, and **3(BPh$_4$)$_2$**.

**compound**	**1(PF$_6$)$_2$**	**2(PF$_6$)$_2$**	**3(BPh$_4$)$_2$**
empirical formula	$C_{20}H_{27}F_{12}N_7P_2Ru$	$C_{46}H_{57}ClF_{18}N_{15}P_3Ru_2$	$C_{68}H_{69}B_2N_9Ru$
moiety formula	$C_{18}H_{24}N_6Ru^{2+}$, 2(F_6P^-), C_2H_3N	2($C_{20}H_{24}N_6Ru^{2+}$), 3(F_6P^-), 3(C_2H_3N), Cl^-	$C_{18}H_{26}N_8Ru^{2+}$, 2($C_{24}H_{20}B^-$), C_2H_3N
formula weight	756.49	1492.56	1135.01
T [K]	100(2)	100(2)	100(2)
crystal size [mm^3]	0.440 x 0.310 x 0.183	0.364 x 0.086 x 0.085	0.471 x 0.355 x 0.224
crystal system	monoclinic	monoclinic	triclinic
space group	$P2_1/c$ (No. 14)	$P2_1$ (No. 4)	*P*–1 (No. 2)
a [Å]	9.8498(5)	8.8490(3)	11.4108(7)
b [Å]	18.3734(11)	30.6413(11)	14.0587(9)
c [Å]	16.2054(10)	10.9812(4)	18.9439(13)
α [°]	90	90	83.178(2)
β [°]	104.504(2)	92.8050(10)	76.210(2)
γ [°]	90	90	88.524(2)
V [Å^3]	2839.3(3)	2973.93(18)	2930.5(3)
Z	4	2	2
ρ [g·cm^{-3}]	1.770	1.667	1.286
F(000)	1512	1500	1188
μ [mm^{-1}]	0.769	0.738	0.318
T_{min} / T_{max}	0.80 / 0.87	0.85 / 0.94	0.87 / 0.93
θ–range [°]	2.136 – 27.978	2.284 – 27.958	2.229 - 28.026
hkl–range	±12, ±24, ±21	±11, ±40, ±14	±15, ±18, ±24
measured refl.	110165	96498	75638
unique refl. [R_{int}]	6806 [0.0454]	14266 [0.0586]	14088 [0.0463]
observed refl. ($I > 2\sigma(I)$)	6153	13230	12491
data / restr. / param.	6806 / 0 / 398	14266 / 64 / 853	14088 / 8 / 754
goodness–of–fit (F^2)	1.076	1.067	1.060
*R*1, *wR*2 ($I > 2\sigma(I)$)	0.0265 / 0.0620	0.0270 / 0.0501	0.0452 / 0.1033
*R*1, *wR*2 (all data)	0.0311 / 0.0641	0.0336 / 0.0532	0.0534 / 0.1087
res. el. dens. [e·Å^{-3}]	–0.555 / 0.848	–0.516 / 0.268	-1.343 / 1.122

Table S2. Selected bond lengths for complexes **1(PF$_6$)$_2$**, **2(PF$_6$)$_2$**, and **3(BPh$_4$)$_2$** in Å.

	**1(PF$_6$)$_2$**	**2(PF$_6$)$_2$**	**2(PF$_6$)$_2$***	**3(BPh$_4$)$_2$**
Ru–N^{NH2-R}	2.155(2) / 2.160(2)	2.142(3)	2.151(3)	2.129(2) / 2.157(2)
Ru–N^{MeCN}	-	2.039(3)	2.041(3)	-
Ru–N^{py}	2.057(2) - 2.069(2)	2.046(3) - 2.060(3)	2.052(3) - 2.063(3)	2.0747(19) - 2.077(2)
Ru–N(–CH_2–)$_3$	2.074(2)	2.068(3)	2.077(3)	2.0703(18)

*) second crystallographically independent molecule

4. Stability of $3(BPh_4)_2$

Complex **$3(BPh_4)_2$** is stable in acetonitrile under inert conditions but gradually decomposes over several hours when its acetonitrile solution is exposed to air. Figure S37 shows the ^{1}H NMR spectrum of **$3(BPh_4)_2$** in dry, degassed MeCN-d_3 (top) and that of the same sample after exposure to air (bottom). Decomposition products were not identified.

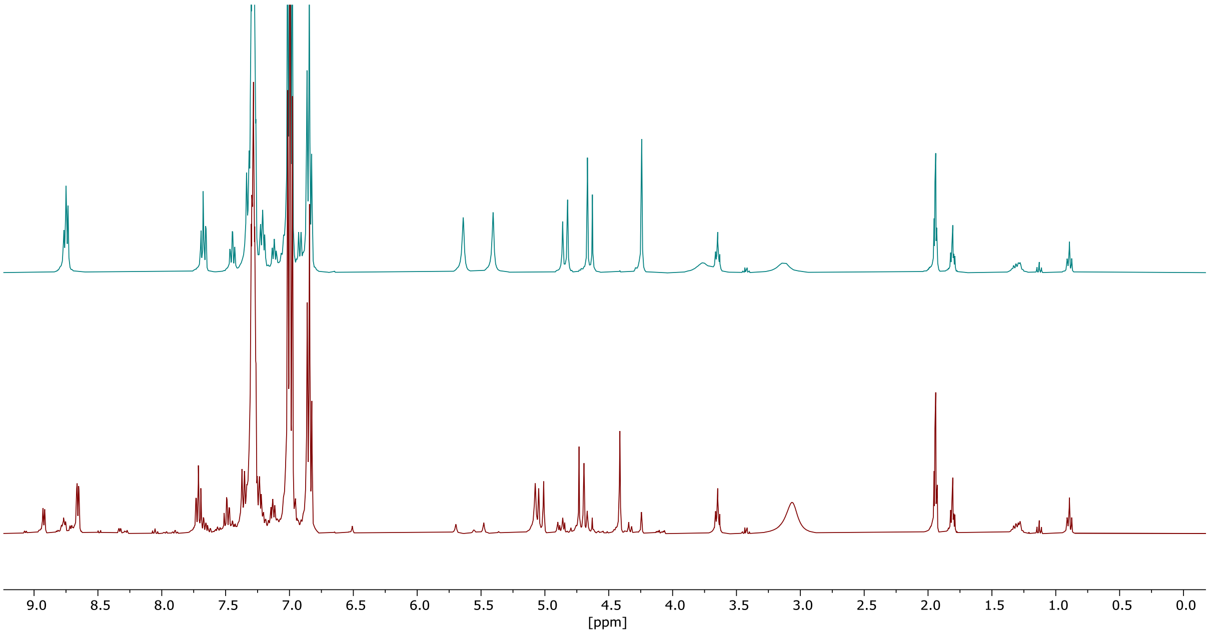

Figure S37. ^{1}H NMR spectra (400 MHz, MeCN-d_3) of complex **$3(BPh_4)_2$** in dry, degassed MeCN-d_3 (top) and after exposure to air (bottom).

5. EPR Spectroscopy

Preliminary EPR experiments were conducted to detect intermediate radical species formed upon oxidation of **1(PF$_6$)$_2$** with *Magic Blue*. A solution of **1(PF$_6$)$_2$** (10 mM) in MeCN was placed in an EPR tube under N_2 atmosphere. Subsequently, 0.5 equivalents of *Magic Blue* in MeCN were added. After being briefly mixed, the sample was immediately (within a few seconds) frozen by immersing the tube into liquid N_2. The grey spectrum shown in Figure S38 was recorded from the resulting frozen solution at 120 K. When repeating the experiment, but keeping the sample at room temperature after addition of *Magic Blue* for 10 min before freezing, no EPR signal was observed (Figure S38, red spectrum). For comparison, also the EPR spectrum of *Magic Blue* in MeCN was recorded under the same conditions (Figure S38, blue spectrum).

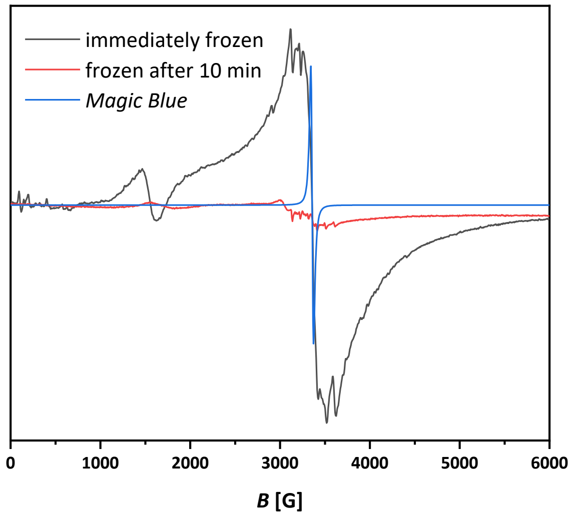

Figure S38. Continuous-wave X-band EPR spectra (ν = 9.43 GHz) of **1(PF$_6$)$_2$** + *Magic Blue* (immediately frozen; grey), **1(PF$_6$)$_2$** + *Magic Blue* (frozen after 10 min; red) and *Magic Blue* (blue) at 120 K from frozen MeCN solutions in the magnetic field range of 0–6000 G.

6. Cyclic Voltammetry

6.1 Scan Rate Dependent CVs of Complexes 2(PF_6)$_2$ and XI(PF_6)$_2$

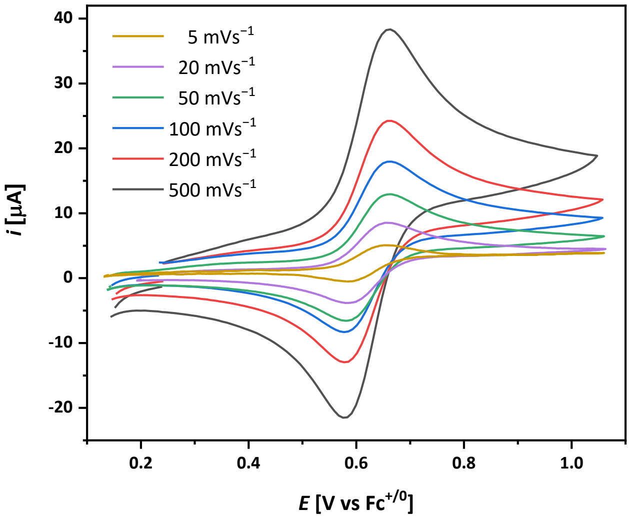

Figure S39. CV of complex **2**(PF_6)$_2$ (1.0 mM) in MeCN with 0.1 M TBAPF$_6$ as supporting electrolyte at various scan rates.

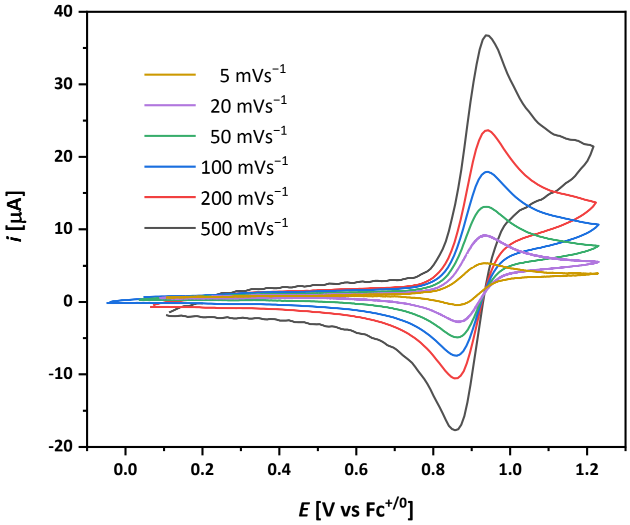

Figure S40. CV of complex **XI**(PF_6)$_2$ (1.0 mM) in MeCN with 0.1 M TBAPF$_6$ as supporting electrolyte at various scan rates.

6.2 CV of Complex $1(PF_6)_2$ in the Presence of Various Bases

Table S3. List of applied bases and their pK_a values in MeCN.

Base	pK_a in MeCN[9]
Benzotriazol	6.9
Pyrazin	7.7
Pyrazol	9.1
Pyridazin	10.1
Chinolin	12.0
Pyridin	12.5
Acridin	12.7
Phenantrolin	13.7

Base	pK_a in MeCN[9]
Imidazol	15.1
DABCO	18.3
Triethylamin	18.8
TMG	23.3
DBN	23.9
DBU	24.3
MTBD	25.5
TBD	26.0

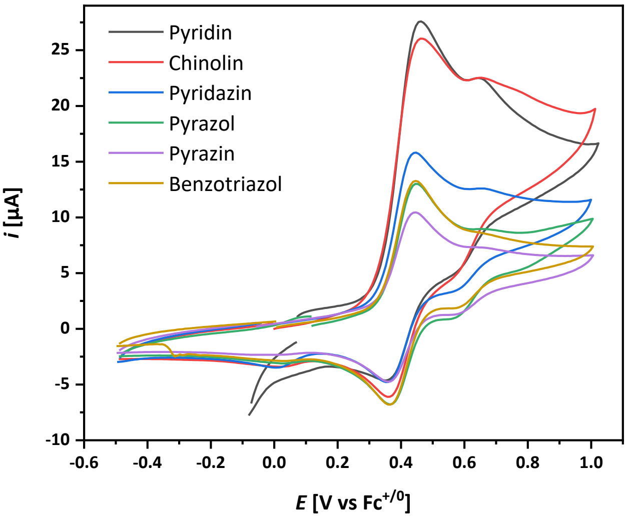

Figure S41. CVs of complex **$1(PF_6)_2$** (1 mM) in the presence of various weak bases (1.05 equiv.) in MeCN with 0.1 M $TBAPF_6$ as supporting electrolyte at a scan rate of 100 mVs^{-1}.

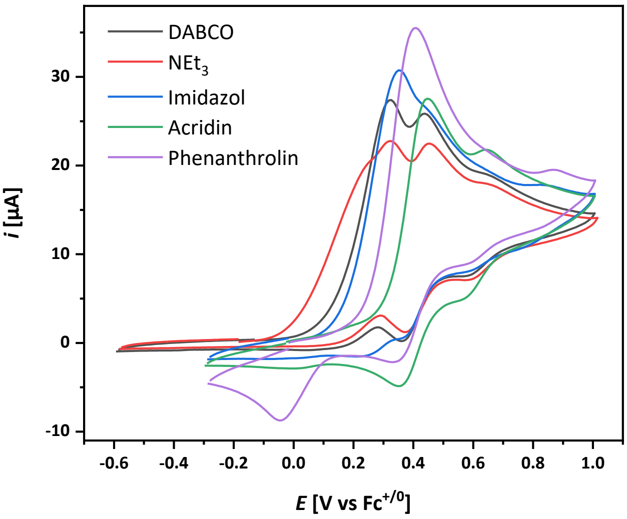

Figure S42. CVs of complex **$1(PF_6)_2$** (1 mM) in the presence of various medium strong bases (1.05 equiv.) in MeCN with 0.1 M $TBAPF_6$ as supporting electrolyte at a scan rate of 100 mVs^{-1}.

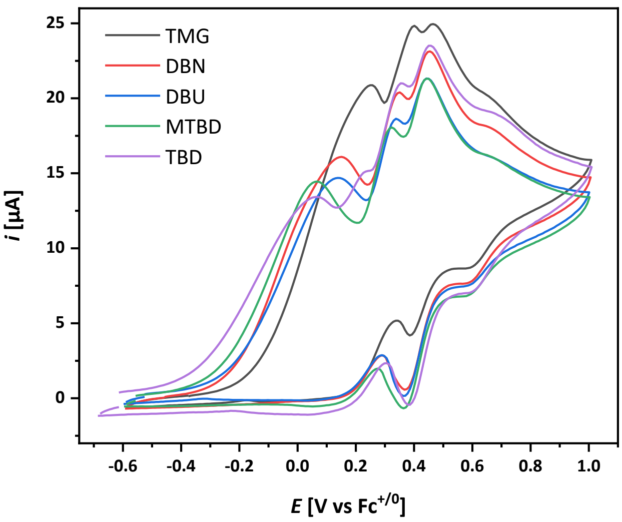

Figure S43. CVs of complex **1(PF$_6$)$_2$** (1 mM) in the presence of various strong bases (1.05 equiv.) in MeCN with 0.1 M TBAPF$_6$ as supporting electrolyte at a scan rate of 100 mVs^{-1}.

6.3 CV of Complexes 1(PF$_6$)$_2$ and 2(PF$_6$)$_2$ in the Presence of Ammonia

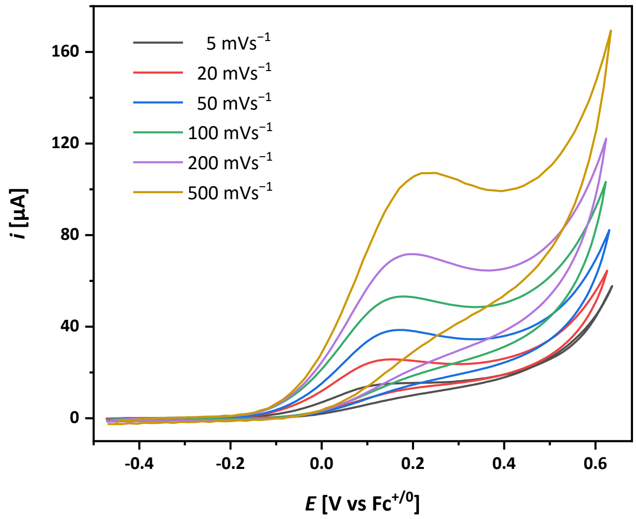

Figure S44. CV of complex **1**(PF$_6$)$_2$ (1.0 mM) in 1.3 M NH_3/MeCN with 0.1 M TBAPF$_6$ as supporting electrolyte at various scan rates.

Note:

As discussed by Costentin and Savéant[10] and by Dempsey and co-workers,[11] a straightforward extraction of an apparent rate constant from the catalytic current is only justified when the cyclic voltammetric response is under purely kinetic control, i.e., when an S-shaped catalytic wave is observed without significant substrate depletion. Outside this regime, the relationship between catalytic current and apparent rate constant is no longer direct. Since catalyst **1^{2+}** does not exhibit a fully developed S-shaped response, even at higher scan rates, we believe that calculating an apparent rate constant would lead to an overinterpretation of the data. Therefore, we have deliberately refrained from reporting such values.

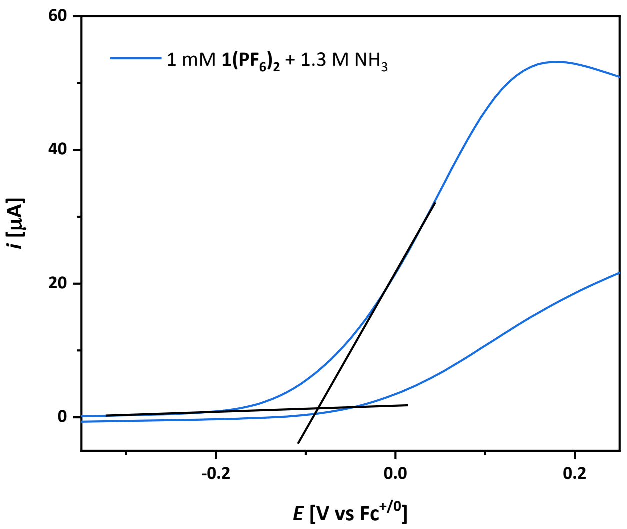

Figure S45. CV of complex **1(PF$_6$)$_2$** (1.0 mM) in 1.3 M NH_3/MeCN with 0.1 M TBAPF$_6$ as supporting electrolyte at scan rate 100 mVs^{-1}. The plot shows the catalytic wave (blue) and the tangents of the base line current and the catalytic wave (black) whose crossing point determine the onset potential.

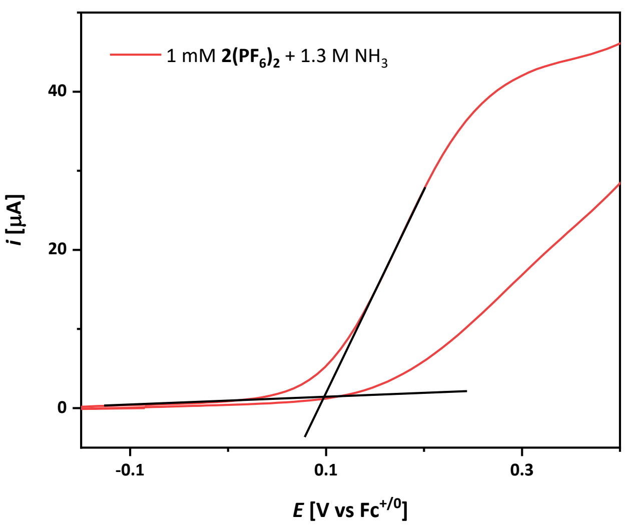

Figure S46. CV of complex **2(PF$_6$)$_2$** (1.0 mM) in 1.3 M NH_3/MeCN with 0.1 M TBAPF$_6$ as supporting electrolyte at scan rate 100 mVs^{-1}. The plot shows the catalytic wave (blue) and the tangents of the base line current and the catalytic wave (black) whose crossing point determine the onset potential.

6.4 CV Simulation

For numerical simulations of the CV data of complex **1(PF$_6$)$_2$**, the following simplified mechanism was used which represents the proposed bimolecular coupling and the following disproportionation in a single chemical step:

E$_1$ $[Ru(TPA)(NH_3)_2]^{2+} \rightarrow [Ru(TPA)(NH_3)_2]^{3+}$

C$_1$ $[Ru(TPA)(NH_3)_2]^{3+} \rightarrow [Ru(TPA)(NH_2)(NH_3)]^{2+} + H^+$

C$_2$ $2\ [Ru(TPA)(NH_2)(NH_3)]^{2+} \rightarrow\rightarrow\rightarrow 2\ [Ru(TPA)(MeCN)_2]^{2+}$

E$_2$ $[Ru(TPA)(MeCN)_2]^{2+} \rightarrow [Ru(TPA)(MeCN)_2]^{3+}$

The starting concentration of $[Ru(TPA)(NH_3)_2]^{2+}$ (1.0 mM) and the electrode surface area (0.07 cm^2) were treated as fixed values. Table S4 summarizes the parameters obtained from the CV simulation. The equilibrium constants *K* were found to have no significant influence on the quality of the fit as long as they are ≫ 1. The reported values should therefore be regarded as representative.

Table S4. Simulation parameters for the CV data of **1(PF$_6$)$_2$**.

	**E$_1$**	**E$_2$**
E^0 [V]	0.40	0.61
α [eV]	0.425	0.575
k [cm/s]	0.018	0.010
D^{red} [cm^2/s]	$8.30 \cdot 10^{-6}$	$1.11 \cdot 10^{-5}$
D^{ox} [cm^2/s]	$1.20 \cdot 10^{-5}$	$9.66 \cdot 10^{-5}$
	**C$_1$**	**C$_2$**
$k_{forward}$	0.055 s^{-1}	$15 \cdot 10^3$ M$^{-1}\cdot$s^{-1}
K	10^5	10^5

Figure S47 to Figure S51 show the obtained simulated CVs in comparison with experimental data at scan rates 500 mVs^{-1}, 200 mVs^{-1}, 100 mVs^{-1}, 50 mVs^{-1} and 20 mVs^{-1}, respectively.

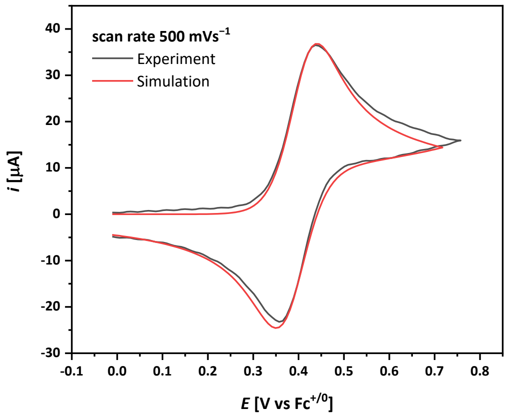

Figure S47. Experimental (grey) and simulated (red) CV of complex **1(PF$_6$)$_2$** (1.0 mM) in MeCN with 0.1 M TBAPF$_6$ as supporting electrolyte at scan rate 500 mVs^{-1}.

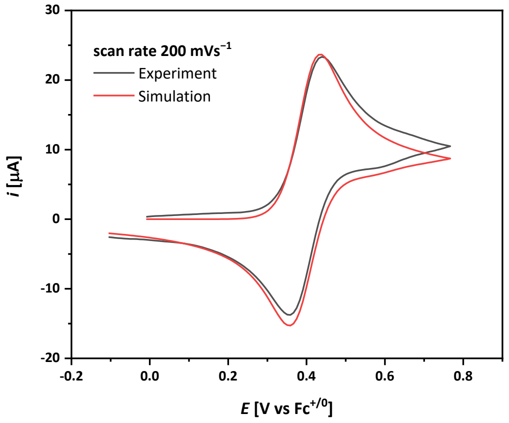

Figure S48. Experimental (grey) and simulated (red) CV of complex **1(PF$_6$)$_2$** (1.0 mM) in MeCN with 0.1 M TBAPF$_6$ as supporting electrolyte at scan rate 200 mVs^{-1}.

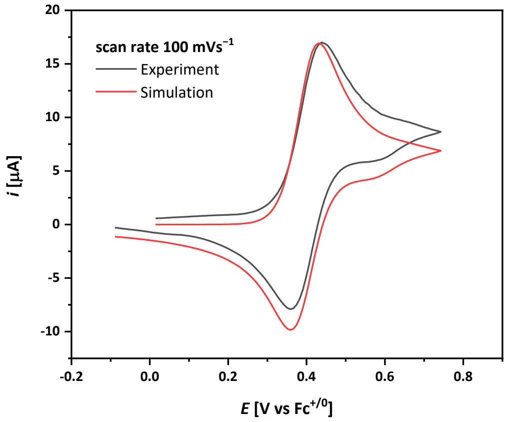

Figure S49. Experimental (grey) and simulated (red) CV of complex **1(PF$_6$)$_2$** (1.0 mM) in MeCN with 0.1 M TBAPF$_6$ as supporting electrolyte at scan rate 100 mVs^{-1}.

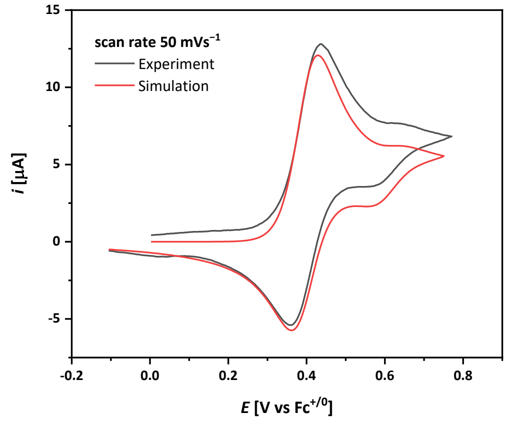

Figure S50. Experimental (grey) and simulated (red) CV of complex **1(PF$_6$)$_2$** (1.0 mM) in MeCN with 0.1 M TBAPF$_6$ as supporting electrolyte at scan rate 50 mVs^{-1}.

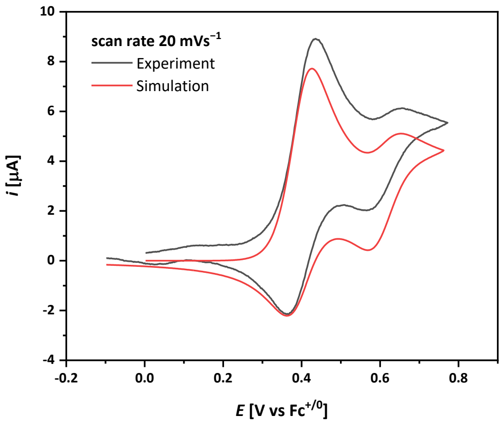

Figure S51. Experimental (grey) and simulated (red) CV of complex **1(PF$_6$)$_2$** (1.0 mM) in MeCN with 0.1 M TBAPF$_6$ as supporting electrolyte at scan rate 20 mVs^{-1}.

7. UV-Vis Kinetic Analysis of the Reaction of 1(PF$_6$)$_2$ with Magic Blue

To gain insight into the rate-determining step during the chemical oxidation of **1(PF$_6$)$_2$** with *Magic Blue*, we performed the reaction several times with a constant concentration of **1(PF$_6$)$_2$** (100 µM) and varying concentrations of *Magic Blue* (20–100 µM) in MeCN while monitoring the reaction progress using UV-Vis spectroscopy, acquiring 1 spectrum per second. Figure S52 shows the spectral changes after addition of 0.6 equivalents *Magic Blue* as a representative example. The spectrum depicted in red corresponds to the starting complex **1(PF$_6$)$_2$** with an absorption maximum at 427 nm. After addition of *Magic Blue*, the band at 427 nm decreases rapidly together with the characteristic *Magic Blue* band at 700 nm, which has completely vanished after ca. 1 s. This spectral change corresponds to the very fast oxidation of **1**$^{2+}$ to **1**$^{3+}$. The resulting green spectrum thus corresponds to a mixture of **1**$^{3+}$ and unreacted **1**$^{2+}$. Over the course of ca. 1 min, a band around 400 nm builds up gradually. We propose that this spectral process reflects the deprotonation of **1**$^{3+}$ to give **4**$^{2+}$ followed by the bimolecular coupling of two equivalents of **4**$^{2+}$ giving **5**$^{4+}$ and its decomposition resulting in the formation of product **2**$^{2+}$ (see mechanism in Scheme 4). The final purple spectrum thus corresponds to a mixture of unreacted **1**$^{2+}$ and product **2**$^{2+}$ with an absorption maximum at 400 nm.

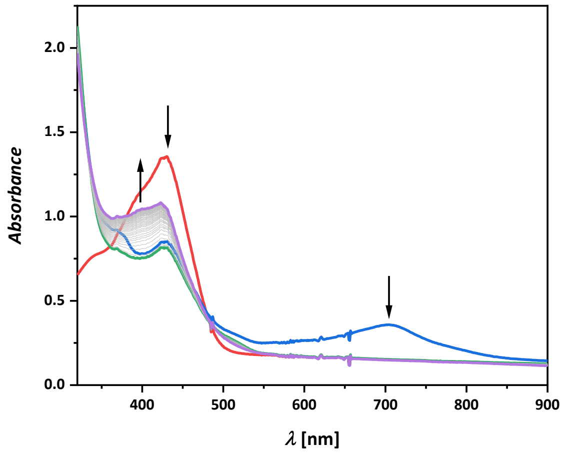

Figure S52. UV-Vis spectra of **1(PF$_6$)$_2$** upon addition of 0.6 equivalents *Magic Blue* in MeCN (1 spectrum per second acquired).

Since the first *Magic Blue*–consuming step is very fast, the applied concentration of *Magic Blue* directly controls the concentration of the formed **1**$^{3+}$. To determine the reaction order of the **2**$^{2+}$ formation with respect to the concentration of **1**$^{3+}$, we determined the initial rate of the formation of **2**$^{2+}$ by evaluating the spectral changes at 400 nm using varying amounts of *Magic Blue*. Figure S53 shows the absorbance at 400 nm as a function of the reaction time for 0.6 equivalents *Magic Blue* as a representative example. The initial absorbance of ca. 1.16 drops rapidly to 0.75 upon addition of *Magic Blue* before the new band slowly builds up to a final absorbance of ca. 1.05. The initial rate was determined from the slope of the increasing absorbance after addition of *Magic Blue*, the fitting curve is depicted in red.

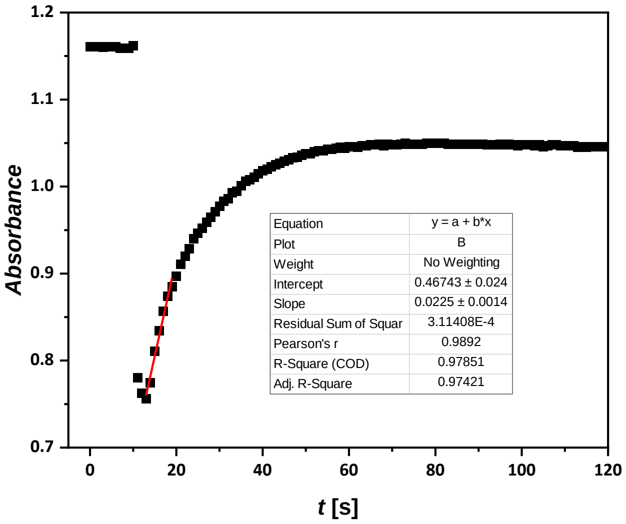

Figure S53. Absorbance at 400 nm as a function of time upon addition of 0.6 equivalents *Magic Blue* to a solution of **1(PF$_6$)$_2$** in MeCN.

Figure S54 shows all obtained initial rates as a function of the applied concentration of *Magic Blue*. As they scale linearly, we conclude that the formation of **2**$^{2+}$ is first order with respect to **1**$^{3+}$. This observation indicates that the deprotonation of **1**$^{3+}$ is the rate-determining step, while the subsequent bimolecular coupling occurs rapidly, in agreement with other experimental and computational findings.

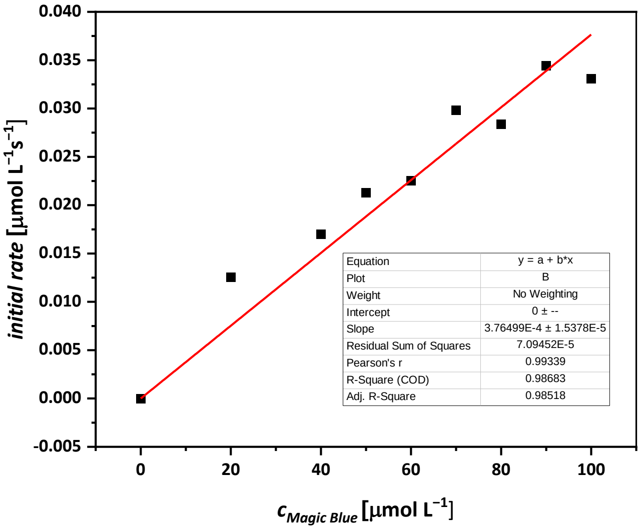

Figure S54. Initial rates as a function of the concentration of *Magic Blue*.

8. Reactivity of XI(PF_6)$_2$ towards Ammonia

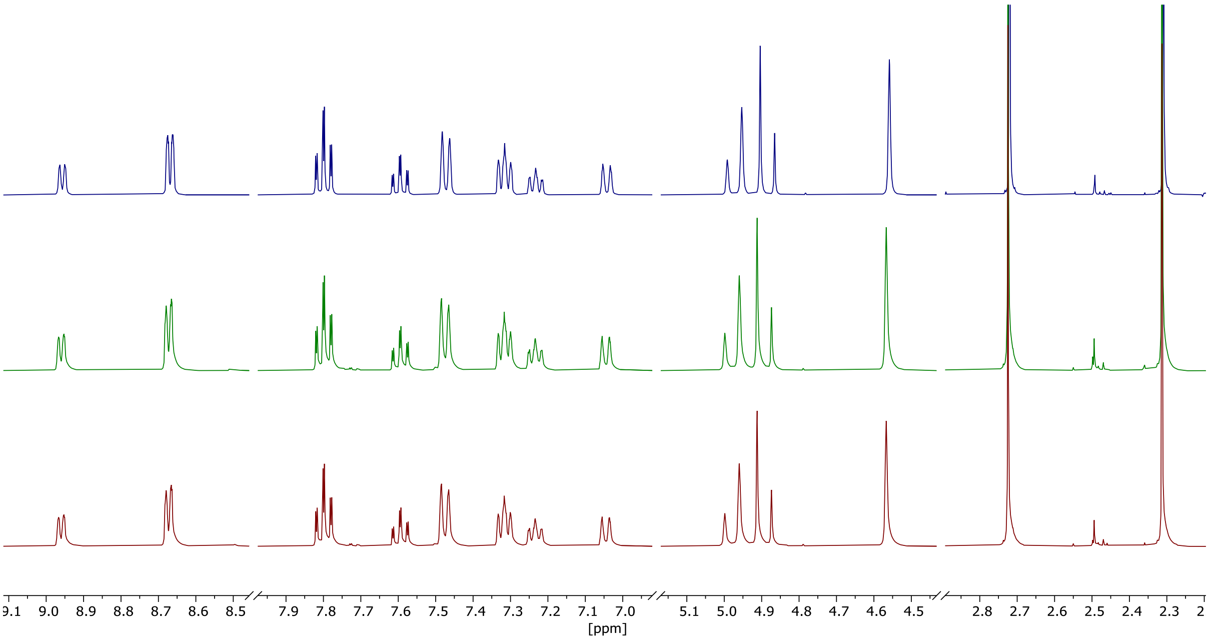

Figure S55. ^{1}H NMR spectra (400 MHz, MeCN-d$_3$) of complex **XI(PF_6)$_2$** in absence of ammonia (top), directly after addition of a large excess of ammonia (middle) and 24 h after addition of ammonia.

9. Controlled Potential Electrolysis

9.1 Electrolysis Experiments with Complex 1(PF_6)$_2$ in the Absence of Ammonia

Bulk electrolysis experiments of a 1 mM solution of complex **1(PF_6)$_2$** in MeCN-d$_3$ with 0.1 M KPF_6 as supporting electrolyte have been performed using a two-compartment cell with a glass frit separator between the cells (compare Figure S58), a glassy carbon rod working electrode, a Ag / 0.01 M $AgNO_3$ reference electrode and a Pt coil counter electrode. CPE were performed at 0.51 V and 0.65 V vs $Fc^{+/0}$, respectively. After completion of the electrolysis, solution from the working electrode compartment was directly used to measure a ^{1}H NMR spectrum in order to identify electrolysis products. Figure S56 shows the spectrum after CPE at 0.51 V vs $Fc^{+/0}$ and, in comparison, the ^{1}H NMR spectrum of complex **2(PF_6)$_2$**, indicating the selective formation of **2**$^{2+}$ from **1**$^{2+}$ upon electrolysis. Figure S57 shows the spectrum after CPE at 0.65 V vs $Fc^{+/0}$ and, in comparison, the ^{1}H NMR spectrum of complex **XI(PF_6)$_2$**. While being a less clean transformation, the spectrum reveals **XI**$^{2+}$ as the major product of electrolysis of **1**$^{2+}$ at higher potential.

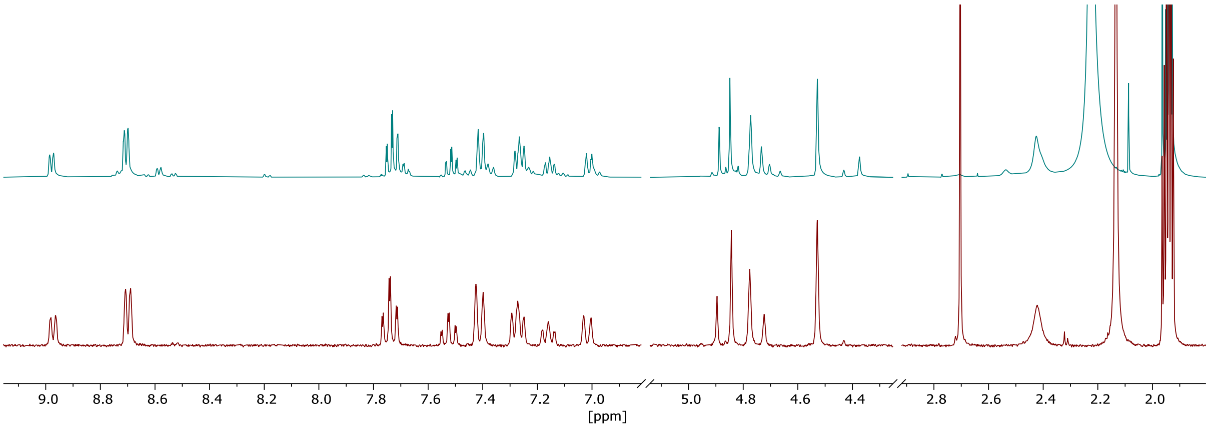

Figure S56. ^{1}H NMR spectrum (400 MHz, MeCN-d$_3$) after CPE of **1(PF_6)$_2$** at 0.51 V vs $Fc^{+/0}$ (top) and, in comparison, the ^{1}H NMR spectrum (400 MHz, MeCN-d$_3$) of complex **2(PF_6)$_2$**.

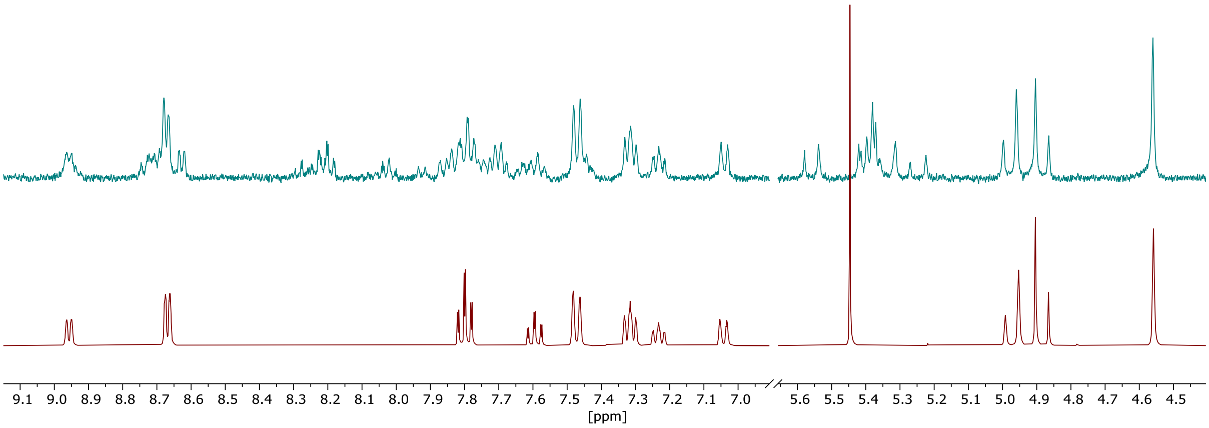

Figure S57. ^{1}H NMR spectrum (400 MHz, MeCN-d$_3$) after CPE of **1(PF_6)$_2$** at 0.65 V vs $Fc^{+/0}$ (top) and, in comparison, the ^{1}H NMR spectrum (400 MHz, MeCN-d$_3$) of complex **XI(PF_6)$_2$**.

9.2 Electrolysis Experiments for Electrocatalytic Ammonia Oxidation

Figure S58 shows a schematic drawing of the experimental setup used for controlled potential electrolysis (CPE) experiments. A two-compartment cell with a connected headspace and a glass frit separator between the cells was applied. The crucial parts numbered in the drawing are: **1** – Pt coil counter electrode; **2** – Ag / 0.01 M $AgNO_3$ reference electrode; **3** – glassy carbon foam working electrode; **4** – 1 mM complex **1(PF$_6$)$_2$**, 1.3 M NH_3, 0.1 M TBAPF$_6$ in 2 mL MeCN; **5** - 1.3 M NH_3, 0.1 M TBAPF$_6$ in 2 mL MeCN.

Note: We chose to perform the CPE experiments in a two-compartment electrochemical cell to avoid possible interference between the anodic ammonia oxidation process and the cathodic counter-electrode reactions. The physical separation of the working and counter electrodes minimizes the crossover of reactive species and helps prevent the reduction or decomposition of oxidation products at the counter electrode. The use of a two-compartment cell is well precedented in the ammonia oxidation literature, e.g., in reports by Warren *et al.*[1] or Bullock *et al.*[12]

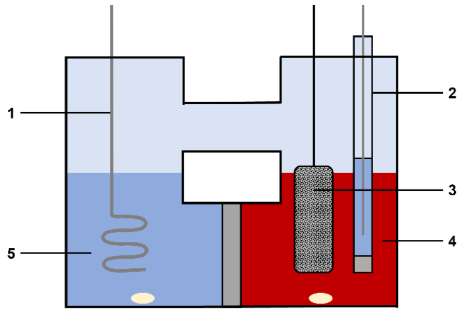

Figure S58: Schematic drawing of the experimental setup used for controlled potential electrolysis (CPE) experiments.

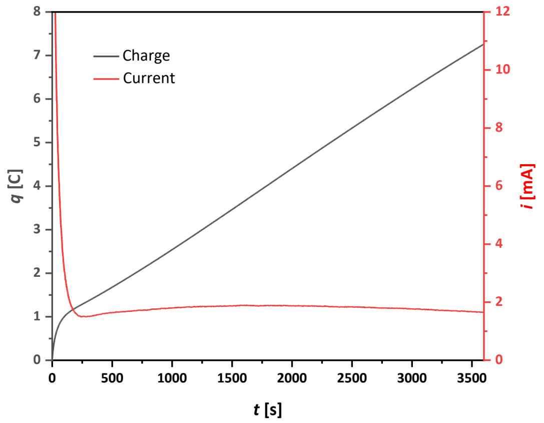

Figure S59. CPE of **1(PF$_6$)$_2$** (1.0 mM) in 1.3 M NH_3/MeCN with 0.1 M TBAPF$_6$ as supporting electrolyte at 0.4 V vs $Fc^{+/0}$ over 1 h – Entry 1. The curves show the current flow (red) and the transferred charge (grey).

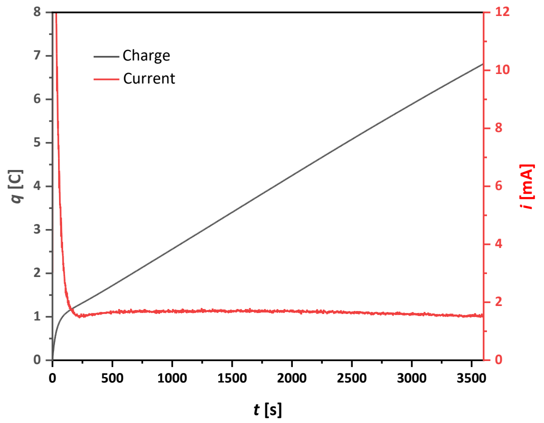

Figure S60. CPE of **1(PF$_6$)$_2$** (1.0 mM) in 1.3 M NH_3/MeCN with 0.1 M TBAPF$_6$ as supporting electrolyte at 0.4 V vs Fc$^{+/0}$ over 1 h – Entry 2. The curves show the current flow (red) and the transferred charge (grey).

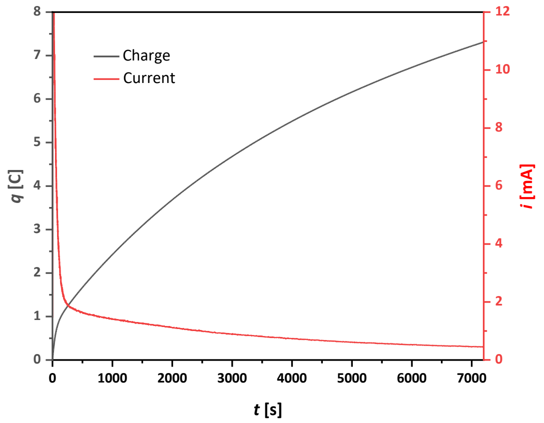

Figure S61. CPE of **1(PF$_6$)$_2$** (1.0 mM) in 1.3 M NH_3/MeCN with 0.1 M TBAPF$_6$ as supporting electrolyte at 0.4 V vs Fc$^{+/0}$ over 2 h – Entry 3. The curves show the current flow (red) and the transferred charge (grey).

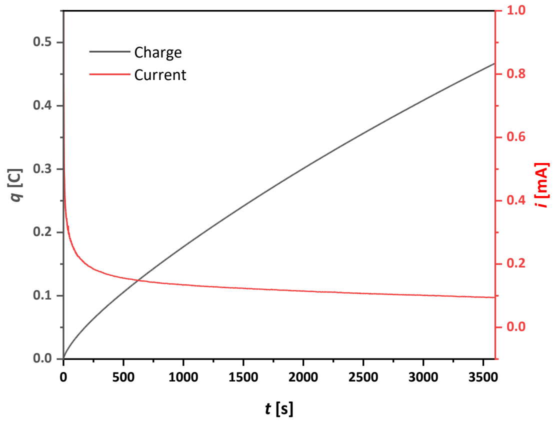

Figure S62. Blank CPE of 1.3 M NH_3/MeCN with 0.1 M TBAPF$_6$ as supporting electrolyte at 0.4 V vs Fc$^{+/0}$ over 1 h. The curves show the current flow (red) and the transferred charge (grey).

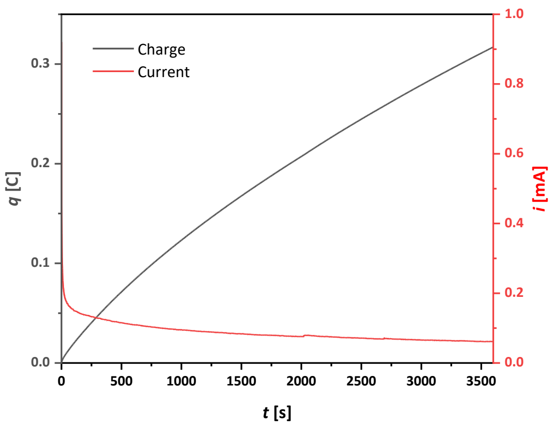

Figure S63. Rinse test after CPE at 0.4 V vs $Fc^{+/0}$ over 1 h. The working electrode was rinsed thoroughly with MeCN and the CPE was repeated in fresh solution in the absence of complex **1(PF$_6$)$_2$** under otherwise same conditions The curves show the current flow (red) and the transferred charge (grey).

In order to analyze the complex species present after CPE of **1(PF$_6$)$_2$** in 1.3 M NH_3/MeCN, we performed an electrolysis experiment using 0.1 M KPF_6 as the supporting electrolyte. After completion of the electrolysis (1 h at 0.4 V vs $Fc^{+/0}$), the solvent was removed under reduced pressure and a ^{1}H NMR spectrum of the solid residue was recorded. Figure S64 shows this spectrum (top) in comparison with the ^{1}H NMR spectra of **2(PF$_6$)$_2$** and **XI(PF$_6$)$_2$**, revealing them as the major products after CPE.

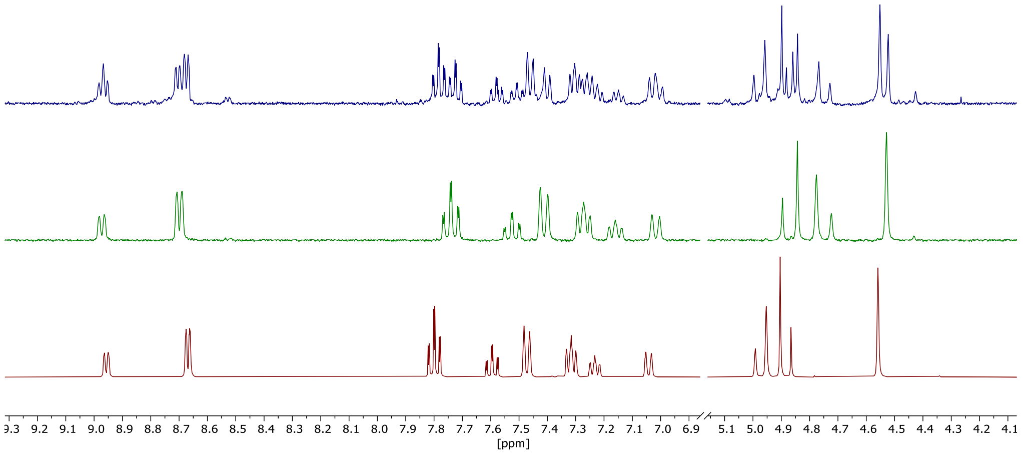

Figure S64. ^{1}H NMR spectrum (400 MHz, MeCN-d$_3$) after CPE of **1(PF$_6$)$_2$** in 1.3 M NH_3/MeCN at 0.4 V vs $Fc^{+/0}$ (top) and, in comparison, the ^{1}H NMR spectra (400 MHz, MeCN-d$_3$) of complexes **1(PF$_6$)$_2$** (middle) and **XI(PF$_6$)$_2$** (bottom).

10. Gas Chromatography

GC analysis of the headspace of the electrolysis cell was carried out after CPE experiments to identify gaseous reaction products. For quantification, calibration curves for both gases of interest (H_2 and N_2) were recorded beforehand. For this purpose, the cell (Figure S58) was fully assembled under Ar atmosphere and filled with 2 mL MeCN per compartment. Then, defined amounts of the respective gas (H_2 or N_2), as well as 200 µL CH_4 as internal standard, were added using a gas-tight syringe. The cell was left for 5 min for equilibration. 1 mL of the headspace was taken using a gas-tight syringe and injected into the GC to record a chromatogram of the respective sample. A representative chromatogram obtained for a volume of 300 µL H_2 is shown in Figure S65.

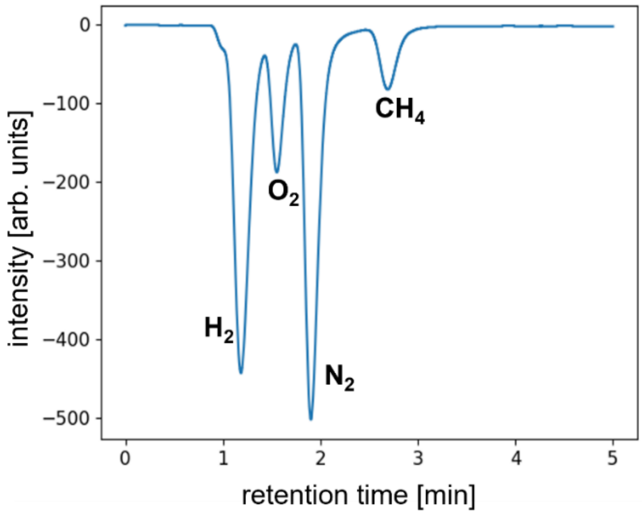

Figure S65: Representative example for a chromatogram obtained for recording a calibration curve for the quantification of H_2. 300 µL H_2 and 200 µL CH_4 (as internal standard) were added to the cell under Ar atmosphere, N_2 and O_2 originate from air contamination.

For obtaining a reliable integration of the partially overlapping peaks, each peak was integrated separately using Exponentially Modified Gaussian functions as proposed by Goodman et al.[13] **Figure** S66 (top) shows the measured curve (black) and the fit (blue), Figure S66 (bottom) shows the individual peak fittings.

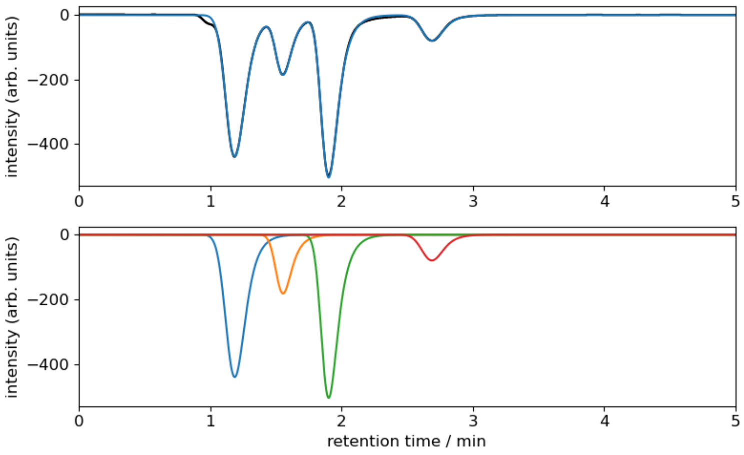

Figure S66: Top – measured chromatogram (black) and fit (blue); Bottom – individual peak fittings.

For obtaining the H_2 calibration curve (Figure S67), the ratio of the integrals of the H_2 peak and the CH_4 peak were plotted against the injected volume of H_2. The fit shows a good linear behavior of the data points.

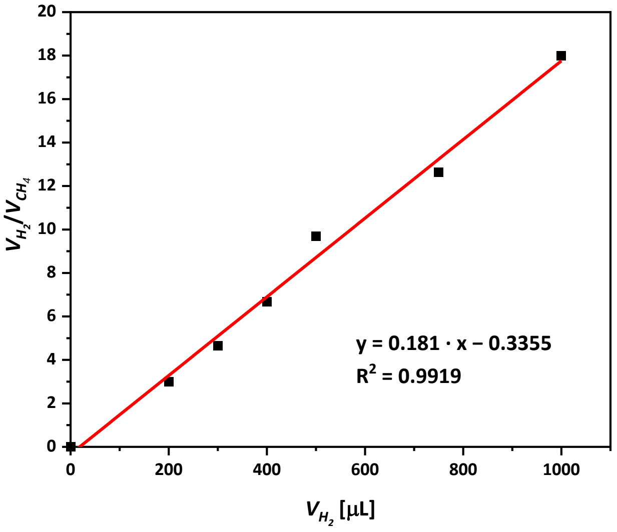

Figure S67: GC calibration curve obtained for H_2. The equation of the linear fit function and the R^2 value are given in the plot.

For obtaining the N_2 calibration curve (Figure S68), the problem of air contamination had to be addressed. For this purpose, the ratio of the integrals of N_2 and O_2 in GC measurements of air was determined as 3.159 (as the average of 3 independent measurements). With this value in hand, the determination of the O_2 integral allows to conclude the amount of N_2 contamination from air that can now be subtracted. The ratio of the air corrected integral of the N_2 peak and the integral of the CH_4 peak were plotted against the injected volume of N_2. The fit shows a good linear behavior of the data points.

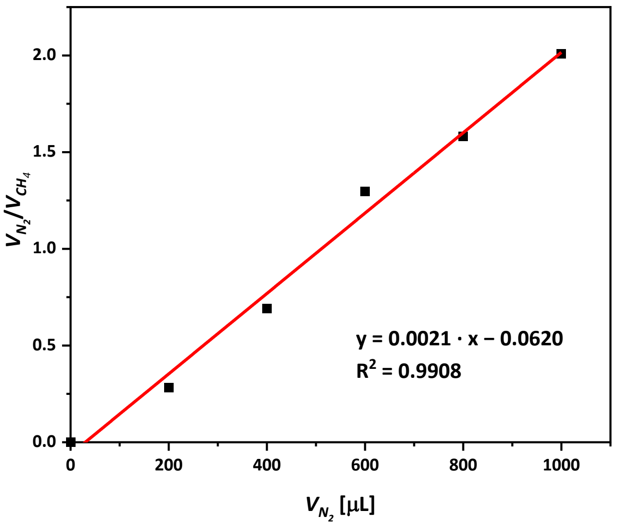

Figure S68: GC calibration curve obtained for N_2 after air correction. The equation of the linear fit function and the R^2 value are given in the plot.

The measured chromatogram and peak fitting for the sample after CPE with 1 mM **1(PF$_6$)$_2$** in 1.3 M NH_3/MeCN with 0.1 M TBAPF$_6$ as the supporting electrolyte at 0.4 V vs $Fc^{+/0}$ is shown in Figure S69.

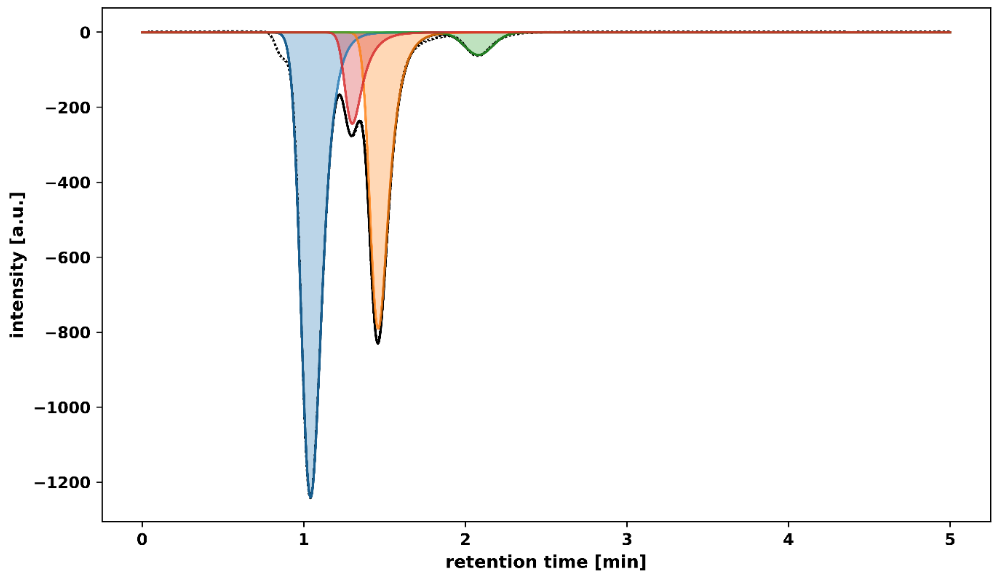

Figure S69. Experimental and fitted GC data obtained from the headspace after CPE with 1 mM **1(PF$_6$)$_2$** in 1.3 M NH_3/MeCN with 0.1 M TBAPF$_6$ as the supporting electrolyte at 0.4 V vs Fc$^{+/0}$. The experimental curve is displayed as a black dotted line, the fit as a black solid line. Individual peak fittings are shown in blue (H_2), red (O_2), orange (N_2) and green (CH_4), respectively.

11. Calculation of Faradaic Efficiencies and Turnover Numbers

Faradaic efficiencies (FE) were calculated as follows:

$$FE = \frac{n(N_2) \cdot 6 \cdot F}{q} \cdot 100 = \frac{n(H_2) \cdot 2 \cdot F}{q} \cdot 100$$

F is Faraday's constant, *q* is the total charge that was transferred over the course of the experiment and *n* are the amounts of N_2 or H_2 that were formed as reaction products as determined in GC experiments. The FE was determined from N_2 and H_2 as shown:

$$FE_{N_2} = \frac{11.4 \cdot 10^{-6} mol \cdot 6 \cdot 96485 \frac{C}{mol}}{7.4\ C} \cdot 100 = 90\ \%$$

$$FE_{H_2} = \frac{30.1 \cdot 10^{-6} mol \cdot 2 \cdot 96485 \frac{C}{mol}}{7.4\ C} \cdot 100 = 79\ \%$$

The turnover number (TON) were calculated as follows, with *n*(N_2) being the amount of N_2 that was formed as reaction product as determined in GC experiments, and *n*(cat) being the amount of catalyst (complex **1(PF$_6$)$_3$**) used in the respective experiment:

$$TON = \frac{n(N_2)}{n(cat)} = \frac{11.4 \cdot 10^{-6} mol}{2.00 \cdot 10^{-6} mol} = 6$$

12. Labeling Experiment

For proving the formation of N_2 from NH_3, the CPE experiment described in section 9.2 using the setup depicted in Figure S58 was repeated at a working potential of 0.4 V vs $Fc^{+/0}$, but using a ^{15}N-labeled solution of NH_3 in acetonitrile (ca. 1 M). The headspace of the electrolysis cell was analyzed using a mass spectrometric gas analyzer during the electrolysis that was conducted for 45 min. The evolution of a signal at m/z = 30 upon electrolysis (Figure S70) indicated the formation of $^{30}N_2$.

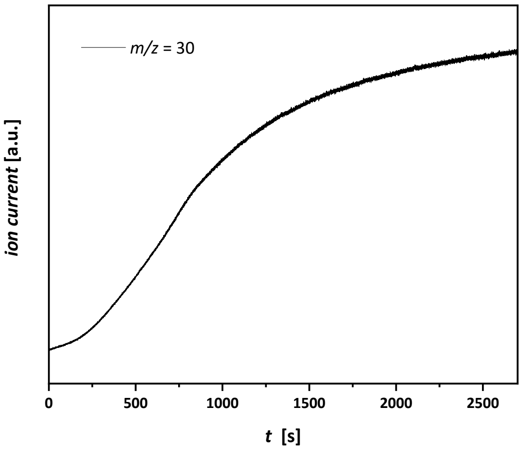

Figure S70. Mass spectrometric monitoring of the headspace of the electrolysis cell at m/z = 30 during CPE with 1 mM **1(PF₆)₂** in 1.3 M NH_3/MeCN with 0.1 M $TBAPF_6$ as the supporting electrolyte at 0.4 V vs $Fc^{+/0}$.

13. Electrode Surface Analysis (XPS)

After CPE with **1(PF₆)₂** in 1.3 M NH_3/MeCN and 0.1 M $TBAPF_6$ as supporting electrolyte at 0.4 V vs $Fc^{+/0}$, the glassy carbon foam working electrode was analyzed by X-ray Photoelectron Spectroscopy to assess possible ruthenium deposition at the electrode surface. Spectra were acquired at multiple locations across the electrode, all showing consistent features. Figure S71 displays one representative spectrum. The red lines indicate the binding energies of Ru core-level electrons in the relevant energy range. However, no corresponding signals can be observed in the spectrum (the large peak at 284.9 eV is assigned to the C 1s level). These results indicate that no detectable ruthenium deposition occurred on the electrode surface during CPE within the detection limits of XPS.

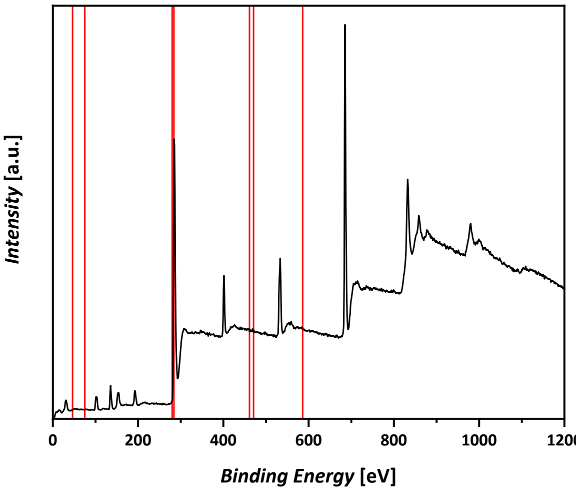

Figure S71. XPS spectrum acquired on the GC foam electrode surface after CPE at 0.4 V vs $Fc^{+/0}$. Red lines indicate the binding energies of Ru core-level electrons.

14. Computational Details

All calculations were performed by using the ORCA quantum chemical program package (version 5.0.4).[14] Geometry optimizations were performed with the B3LYP functional in conjunction with the triple-ζ def2-TZVP basis set (Ru) or the def2-SVP basis set (C, N, H), respectively.[15–17] Frequency calculations were carried out at the same level to ensure that all geometries were indeed local minima on the potential energy surface. Zero-point energy, thermo corrections, enthalpic and entropic contributions at 298.15 K also were obtained from frequency calculations. The CPCM (acetonitrile) solvation model has been applied to take into account solvent effects.[18] RIJCOSX approximations were used to accelerate the calculations in combination with the auxiliary basis set def2/J.[19,20] The noncovalent interactions were considered via atom-pairwise dispersion corrections with Becke-Johnson (D3BJ) damping.[21] For those cases having difficulties in SCF convergence, damping parameters were altered using the SlowConv function.[22] Solvation energies were obtained from single point energies in solution and gas phase.

Calculation of pK_a values

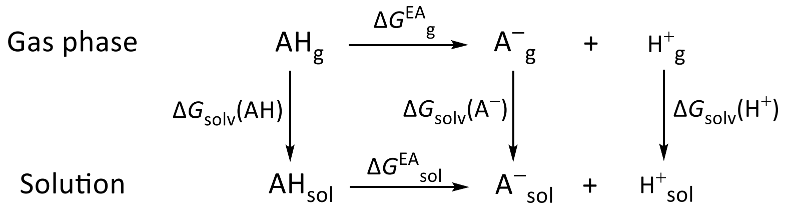

Scheme S1. Thermodynamic cycle used for the calculations of pK_a values.

The pK_a value for a deprotonation reaction was calculated by using the Gibbs free energy change $\Delta G_{\mathrm{sol}}^{\mathrm{PL}}$.

$$\mathrm{p}K_{\mathrm{a}} = \frac{\Delta G_{\mathrm{sol}}^{\mathrm{PL}}}{\ln(10) \cdot \mathrm{RT}}$$

F represents the Faraday constant, *R* the gas constant and *T* the temperature. $\Delta G_{\mathrm{sol}}^{\mathrm{PL}}$ was computed according the thermodynamic cycle depicted in Scheme S1:

$$\Delta G_{\mathrm{sol}}^{\mathrm{PL}} = \Delta G_{\mathrm{g}}^{\mathrm{PL}} + \Delta G_{\mathrm{sol}}(\mathrm{A}^-) + \Delta G_{\mathrm{sol}}(\mathrm{H}^+) - \Delta G_{\mathrm{sol}}(\mathrm{AH})$$

$$\Delta G_{\mathrm{g}}^{\mathrm{PL}} = G_{\mathrm{g}}(\mathrm{A}^-, 1\ \mathrm{atm}) - G_{\mathrm{g}}(\mathrm{AH}, 1\ \mathrm{atm}) + G_{\mathrm{g}}(\mathrm{H}^+, 1\ \mathrm{atm})$$

The gas phase Gibbs energy change was corrected to the standard state (1 M) using the following equation:

$$\Delta G_{\mathrm{gas}}^{\mathrm{PL}}(1\ \mathrm{M}) = \Delta G_{\mathrm{gas}}^{\mathrm{PL}}(1\ \mathrm{atm}) + 1.89$$

Values for $\Delta G_{\mathrm{sol}}(\mathrm{H}^+)$ and $G_{\mathrm{g}}(\mathrm{H}^+, 1\ \mathrm{atm})$ were adapted from works by Kelly, Cramer and Truhlar[23] and by Fifen, Dahaouadi and Nsangou,[24] respectively.

Calculation of redox potentials

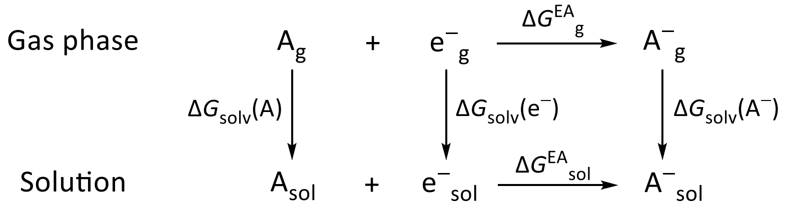

Scheme S2. Thermodynamic cycle used for the calculations of redox potentials.

The redox potential of an electron transfer reaction in solution was calculated by using the Gibbs free energy change ΔG^{EA}_{sol}.

$$E^0 = \frac{-\Delta G^{EA}_{sol}}{F}$$

F represents the Faraday constant and ΔG^{EA}_{sol} was computed according the thermodynamic cycle depicted in Scheme S2:

$$\Delta G^{EA}_{sol} = \Delta G^{EA}_{g} + \Delta G_{sol}(A^-) - \Delta G_{sol}(A) - \Delta G_{sol}(e^-)$$

$$\Delta G^{EA}_{g} = G_g(A^-) - G_g(e^-) - G_g(A)$$

The Gibbs free energy of an electron in the gas phase, $G_g(e^-)$, is considered as 0. Since the solvation energy of the electron is difficult to determine and constitutes a major source of error, all redox potentials were instead calculated relative, i.e., without accounting for the electron solvation. The chemically similar reference system $\mathbf{XI}^{2+}$ with a known experimental redox potential was also computed. The difference between the relative computed and experimental potential of $\mathbf{XI}^{2+}$ was then applied as a correction term to all other potentials.

Calculation of Bond Dissociation Free Energies (BDFEs)

All computed BDFEs discussed in this work were derived from computed pK_a values and redox potentials using the Bordwell equation, with the constant $C_{G,\ MeCN}$ = 52.6 kcal·mol^{-1} taken from literature.[25]

$$BDFE\ [\mathrm{kcal \cdot mol^{-1}}] = 1.37 \cdot \mathrm{p}K_a + 23.06 \cdot E^0[\mathrm{V}] + C_{G,MeCN}$$

Calculation of the reaction barrier for the N–N bond formation step

For locating the transition state of the bimolecular coupling reaction between two molecules $\mathbf{4}^{2+}$ to form $\mathbf{5}^{4+}$, we have performed a potential energy surface scan by varying the distance between the NH_2 units of two molecules $\mathbf{4}^{2+}$. In an initial scan, the $NH_2 \cdots NH_2$ distance was varied from 1.0 Å to 5.0 Å in 10 steps (Figure S72, grey). To further refine the transition state geometry, we performed a second potential energy surface scan around the identified local maximum on the reaction trajectory between

1.9 Å and 2.6 Å in 20 steps (Figure S72, red). The hereby obtained highest-energy geometry was then optimized as the transition state. A numerical frequency calculation was carried out to confirm the transition state nature of the obtained geometry. The calculation delivered two imaginary vibrational modes with a magnitude of 526 cm^{-1} and 14 cm^{-1}, respectively. While the high-frequency mode corresponds to a vibration along the projected reaction trajectory, the low-frequency mode is a simple rotational movement of one NH_3 ligand remote from the reaction center and is therefore attributed to a computational artefact.

The activation energy of the N−N bond formation step (6.2 kcal/mol) was then estimated as the difference between the single point energy of the transition state and the single point energy of two molecules **4**$^{2+}$.

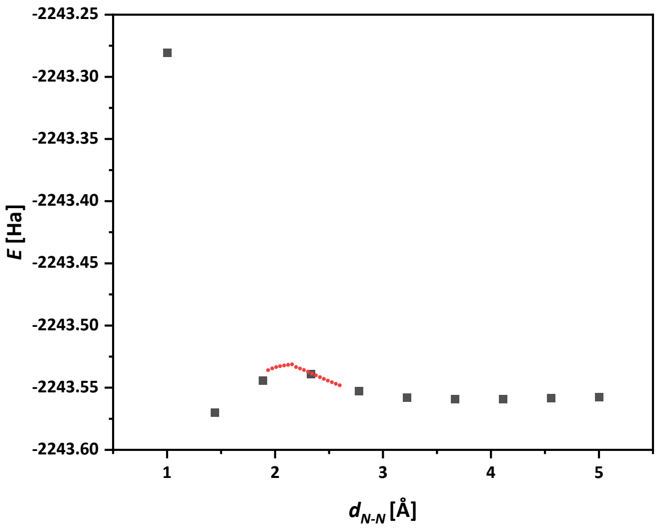

Figure S72. Potential energy surface scan for the bimolecular coupling step between two molecules **4**$^{2+}$ to form **5**$^{4+}$.

References

(1) Ahmed, M. E.; Raghibi Boroujeni, M.; Ghosh, P.; Greene, C.; Kundu, S.; Bertke, J. A.; Warren, T. H. Electrocatalytic Ammonia Oxidation by a Low-Coordinate Copper Complex. *J. Am. Chem. Soc.* **2022**, *144* (46), 21136–21145.

(2) Bennett, M. A.; Huang, T.-N.; Matheson, T. W.; Smith, A. K.; Ittel, S.; Nickerson, W. 16. (η 6 - Hexamethylbenzene)Ruthenium Complexes. In *Inorganic Syntheses*; Fackler, J. P., Ed.; Wiley, 1982; pp 74–78. DOI: 10.1002/9780470132524.ch16.

(3) Whiteoak, C. J.; Nobbs, J. D.; Kiryushchenkov, E.; Pagano, S.; White, A. J. P.; Britovsek, G. J. P. Tri(pyridylmethyl)phosphine: the elusive congener of TPA shows surprisingly different coordination behavior. *Inorg. Chem.* **2013**, *52* (12), 7000–7009.

(4) *DigiElch Professional, version 7*; ElchSoft, 2013.

(5) Sheldrick, G. M. SHELXT - integrated space-group and crystal-structure determination. *Acta Cryst. A* **2015**, *71* (Pt 1), 3–8.

(6) Sheldrick, G. M. Crystal structure refinement with SHELXL. *Acta Cryst. C,* **2015**, *71* (Pt 1), 3–8.

(7) *SADABS*; BRUKER AXS GmbH Karlsruhe, 2016.

(8) Stoll, S.; Schweiger, A. EasySpin, a comprehensive software package for spectral simulation and analysis in EPR. *J. Magn. Reson.* **2006**, *178* (1), 42–55.

(9) Tshepelevitsh, S.; Kütt, A.; Lõkov, M.; Kaljurand, I.; Saame, J.; Heering, A.; Plieger, P. G.; Vianello, R.; Leito, I. On the Basicity of Organic Bases in Different Media. *Eur. J. Org. Chem.* **2019**, *2019* (40), 6735–6748.

(10) Costentin, C.; Savéant, J.-M. Multielectron, Multistep Molecular Catalysis of Electrochemical Reactions: Benchmarking of Homogeneous Catalysts. *ChemElectroChem* **2014**, *1* (7), 1226–1236.

(11) Rountree, E. S.; McCarthy, B. D.; Eisenhart, T. T.; Dempsey, J. L. Evaluation of homogeneous electrocatalysts by cyclic voltammetry. *Inorg. Chem.* **2014**, *53* (19), 9983–10002.

(12) Liu, L.; Johnson, S. I.; Appel, A. M.; Bullock, R. M. Oxidation of Ammonia Catalyzed by a Molecular Iron Complex: Translating Chemical Catalysis to Mediated Electrocatalysis. *Angew. Chem.* **2024**, *136* (41).

(13) Goodman, K. J.; Brenna, J. T. Curve fitting for restoration of accuracy for overlapping peaks in gas chromatography/combustion isotope ratio mass spectrometry. *Anal. Chem.* **1994**, *66* (8), 1294–1301.

(14) Neese, F. Software update: The ORCA program system—Version 5.0. *WIREs Comput. Mol. Sci.* **2022**, *12* (5).

(15) Schäfer, A.; Horn, H.; Ahlrichs, R. Fully optimized contracted Gaussian basis sets for atoms Li to Kr. *J- Chem. Phys.* **1992**, *97* (4), 2571–2577.

(16) Weigend, F.; Ahlrichs, R. Balanced basis sets of split valence, triple zeta valence and quadruple zeta valence quality for H to Rn: Design and assessment of accuracy. *Phys. Chem. Chem. Phys.* **2005**, *7* (18), 3297–3305.

(17) Weigend, F. Hartree-Fock exchange fitting basis sets for H to Rn. *J. Comput. Chem.* **2008**, *29* (2), 167–175.

(18) Barone, V.; Cossi, M. Quantum Calculation of Molecular Energies and Energy Gradients in Solution by a Conductor Solvent Model. *J. Phys. Chem. A* **1998**, *102* (11), 1995–2001.

(19) Eichkorn, K.; Treutler, O.; Öhm, H.; Häser, M.; Ahlrichs, R. Auxiliary basis sets to approximate Coulomb potentials. *Chem. Phys. Lett.* **1995**, *240* (4), 283–290.

(20) Neese, F.; Wennmohs, F.; Hansen, A.; Becker, U. Efficient, approximate and parallel Hartree–Fock and hybrid DFT calculations. A 'chain-of-spheres' algorithm for the Hartree–Fock exchange. *Chem. Phys.* **2009**, *356* (1-3), 98–109.

(21) Grimme, S.; Ehrlich, S.; Goerigk, L. Effect of the damping function in dispersion corrected density functional theory. *J. Comput. Chem.* **2011**, *32* (7), 1456–1465.

(22) Grimme, S.; Antony, J.; Ehrlich, S.; Krieg, H. A consistent and accurate ab initio parametrization of density functional dispersion correction (DFT-D) for the 94 elements H-Pu. *J. Chem. Phys.* **2010**, *132* (15), 154104.

(23) Kelly, C. P.; Cramer, C. J.; Truhlar, D. G. Single-ion solvation free energies and the normal hydrogen electrode potential in methanol, acetonitrile, and dimethyl sulfoxide. *J. Phys. Chem. B* **2007**, *111* (2), 408–422.

(24) Fifen, J. J.; Dhaouadi, Z.; Nsangou, M. Revision of the thermodynamics of the proton in gas phase. *J. Phys. Chem. A* **2014**, *118* (46), 11090–11097.

(25) Agarwal, R. G.; Wise, C. F.; Warren, J. J.; Mayer, J. M. Correction to Thermochemistry of Proton-Coupled Electron Transfer Reagents and its Implications. *Chem. Rev.* **2022**, *122* (1), 1482.
