# Supplementary material for: Ammonia Oxidation with a Ruthenium Polypyridyl Complex: Mechanism-Guided Approach to Low Overpotential Electrocatalysis
Source: JACS Au. 2026 Jul 11;6(7):4253–68. doi: 10.1021/jacsau.6c00733 (PMC13417302; doi:10.1021/jacsau.6c00733)
Supplement: Supplementary file 2 [file au6c00733_si_002.pdf]

DFT-optimized Geometries

In the following, the DFT-optimized geometries of all calculated species are provided as atomic xyz coordinates. For details on the methodology, refer to the SI. The *cis*/*trans* designation refers to the relative position with respect to the sp^3-hybridized nitrogen atom of the TPA ligand.

$[Ru(TPA)(NH_3)_2]^{2+}$ ($\mathbf{1^{2+}}$), S = 0, gas phase

Ru	0.379300	11.907874	6.627973
N	-0.844907	13.296959	5.641243
N	0.030895	10.854383	4.852067
N	1.880323	13.089763	5.830280
N	0.331004	13.345561	8.144901
N	1.716356	10.441581	7.547350
N	-1.254498	10.787726	7.557050
C	-1.797229	12.474285	4.844393
C	-1.046399	11.341934	4.173074
C	-1.491584	14.109644	6.708253
C	-0.552221	14.345090	7.871275
C	-0.007346	14.128766	4.716446
C	0.735161	9.846831	4.304963
C	1.447532	14.140789	5.094006
C	3.192526	13.015773	6.131620
C	1.148550	13.480573	9.204727
C	-1.433174	10.817685	2.942593
C	-0.623799	15.492503	8.657990
C	0.398039	9.266981	3.083231
C	2.315615	15.135914	4.645534
C	4.119681	13.960387	5.705343
C	1.119993	14.591551	10.044672
C	-0.708777	9.756480	2.389235
C	0.218385	15.619517	9.766775
C	3.674860	15.046421	4.947866
H	-1.733194	10.143470	6.918895
H	-0.936457	10.203064	8.336331
H	-1.990434	11.369136	7.968977
H	2.566137	10.285485	6.997015
H	1.298487	9.509885	7.639670
H	2.048139	10.677892	8.487699
H	-2.341534	13.080883	4.101088
H	-2.553101	12.069657	5.534080
H	-2.365784	13.543905	7.067793
H	-1.877645	15.064005	6.315130
H	-2.295192	11.237220	2.419647
H	-1.000508	9.328395	1.427713
H	1.000972	8.448085	2.686909

H	1.602650	9.495975	4.867276
H	-0.085191	13.690666	3.709334
H	-0.410689	15.150594	4.645609
H	1.927013	15.971161	4.059130
H	4.372998	15.812072	4.602900
H	5.171479	13.846065	5.973035
H	3.510786	12.173015	6.745696
H	-1.336527	16.280641	8.406519
H	0.173317	16.509854	10.397710
H	1.801155	14.646664	10.895676
H	1.858086	12.670339	9.384118

$[Ru(TPA)(NH_3)_2]^{2+}$ (1^{2+}), *S* = 0, solution

Ru	0.379882	11.910095	6.608877
N	-0.846606	13.302429	5.625649
N	0.028731	10.855037	4.852663
N	1.877574	13.096363	5.821496
N	0.340738	13.333879	8.120897
N	1.680955	10.446118	7.521293
N	-1.224936	10.800949	7.537526
C	-1.797475	12.477235	4.834103
C	-1.048248	11.339808	4.173653
C	-1.486312	14.112142	6.695817
C	-0.540137	14.336449	7.853580
C	-0.009832	14.132524	4.704903
C	0.725152	9.831890	4.326028
C	1.439511	14.155206	5.098377
C	3.184997	13.029517	6.143677
C	1.168527	13.456970	9.174061
C	-1.440903	10.800251	2.951768
C	-0.596922	15.483860	8.642066
C	0.378739	9.237594	3.113811
C	2.300306	15.171807	4.685569
C	4.103368	13.998140	5.750955
C	1.152432	14.569116	10.013178
C	-0.724807	9.726701	2.413737
C	0.255567	15.603659	9.742905
C	3.655324	15.094048	5.009557
H	-1.648221	10.100130	6.921695
H	-0.893643	10.278033	8.353507
H	-2.002872	11.364989	7.890290
H	2.542369	10.274797	6.995248
H	1.235066	9.526193	7.592453
H	1.985572	10.667965	8.473149
H	-2.336287	13.078776	4.083799

H	-2.548039	12.066959	5.524815
H	-2.355151	13.546320	7.064931
H	-1.863981	15.071505	6.308685
H	-2.301418	11.223872	2.430284
H	-1.021372	9.287168	1.458762
H	0.973895	8.406435	2.731398
H	1.585717	9.482588	4.899283
H	-0.070179	13.686098	3.700444
H	-0.417135	15.151234	4.623100
H	1.902115	16.012941	4.114613
H	4.347431	15.878369	4.695266
H	5.151444	13.892678	6.036567
H	3.499958	12.177033	6.745581
H	-1.306228	16.274105	8.388703
H	0.223736	16.495450	10.372803
H	1.843486	14.618595	10.856586
H	1.871364	12.639080	9.342704

$[Ru(TPA)(NH_3)_2]^{3+}$, S = 1/2, gas phase

Ru	0.444719	11.963635	6.583017
N	-0.802529	13.340693	5.609739
N	0.010616	10.860287	4.865105
N	1.918369	13.148693	5.786520
N	0.301038	13.330591	8.149578
N	1.801192	10.503097	7.487072
N	-1.157733	10.853301	7.578251
C	-1.777865	12.523544	4.819579
C	-1.051955	11.369258	4.168394
C	-1.455323	14.179317	6.666392
C	-0.567593	14.348795	7.877311
C	0.025579	14.166378	4.658353
C	0.677131	9.793966	4.369664
C	1.479443	14.186263	5.024629
C	3.238269	13.059191	6.083882
C	1.044865	13.377689	9.277013
C	-1.454912	10.809941	2.960952
C	-0.687064	15.439349	8.733481
C	0.321465	9.188964	3.168730
C	2.355371	15.154907	4.543212
C	4.165498	13.982494	5.623424
C	0.962542	14.432220	10.178503
C	-0.764553	9.701439	2.452576
C	0.084208	15.484811	9.901214
C	3.718269	15.053337	4.839592
H	-1.497896	10.054717	7.029753

H	-0.864511	10.462092	8.480761
H	-1.992424	11.404817	7.803802
H	2.573763	10.237795	6.865644
H	1.353854	9.611169	7.728642
H	2.259925	10.806748	8.353084
H	-2.304614	13.141013	4.074828
H	-2.547398	12.142343	5.508648
H	-2.382638	13.669626	6.975472
H	-1.765117	15.154323	6.261355
H	-2.304377	11.232466	2.419190
H	-1.071004	9.247650	1.506094
H	0.889843	8.330694	2.803791
H	1.523521	9.421431	4.948792
H	-0.060533	13.715987	3.656549
H	-0.386229	15.183408	4.580268
H	1.977123	15.979271	3.934197
H	4.422473	15.802051	4.466666
H	5.220532	13.867629	5.880885
H	3.554633	12.229482	6.715084
H	-1.384603	16.246394	8.497358
H	-0.001624	16.332810	10.586162
H	1.580410	14.427972	11.079059
H	1.731095	12.549054	9.458543

$[Ru(TPA)(NH_3)_2]^{3+}$, S = 1/2, solution

Ru	0.443713	11.936681	6.559027
N	-0.797691	13.328898	5.582118
N	-0.029639	10.838543	4.861258
N	1.920699	13.161466	5.791558
N	0.307469	13.313733	8.112286
N	1.761738	10.475981	7.415914
N	-1.089071	10.841536	7.578636
C	-1.784462	12.527284	4.799498
C	-1.079606	11.356811	4.165163
C	-1.433679	14.181044	6.630835
C	-0.537900	14.341721	7.831507
C	0.036942	14.134903	4.629810
C	0.617708	9.753479	4.394543
C	1.471978	14.201237	5.047038
C	3.221148	13.106717	6.150033
C	1.065656	13.349104	9.225036
C	-1.491955	10.789090	2.964838
C	-0.619243	15.448698	8.671171
C	0.249941	9.140268	3.201895
C	2.326179	15.220688	4.638262

C	4.128665	14.083664	5.762372
C	1.021060	14.421824	10.107503
C	-0.821872	9.664235	2.476792
C	0.168495	15.490716	9.823584
C	3.674077	15.160303	4.996473
H	-1.338207	9.973892	7.092465
H	-0.769916	10.562371	8.511830
H	-1.970871	11.337646	7.736519
H	2.569412	10.262703	6.821330
H	1.300076	9.573049	7.570326
H	2.161997	10.729091	8.324331
H	-2.289878	13.147676	4.044467
H	-2.556730	12.154562	5.487895
H	-2.357366	13.679766	6.959515
H	-1.725340	15.158278	6.221441
H	-2.330446	11.230435	2.423856
H	-1.133619	9.204826	1.536297
H	0.802084	8.265886	2.854838
H	1.449386	9.376064	4.989082
H	0.004683	13.638002	3.647561
H	-0.394546	15.136652	4.497121
H	1.931786	16.048422	4.046839
H	4.361553	15.950338	4.686676
H	5.171864	14.001062	6.069907
H	3.533844	12.268108	6.771273
H	-1.297531	16.265050	8.418190
H	0.117425	16.352856	10.492095
H	1.653464	14.414179	10.996204
H	1.729773	12.503564	9.403599

$[Ru(TPA)(NH_3)_2]^{4+}$, S = 1, gas phase

Ru	0.466564	11.968599	6.616806
N	-0.802646	13.340480	5.627343
N	0.029970	10.963586	4.860077
N	1.939641	13.118293	5.808114
N	0.281132	13.390449	8.099422
N	1.779944	10.422307	7.428140
N	-1.153844	10.877726	7.623286
C	-1.814729	12.533074	4.856542
C	-1.098067	11.387894	4.198810
C	-1.481350	14.224544	6.650162
C	-0.621062	14.392956	7.869274
C	0.045793	14.134582	4.649871
C	0.779738	9.952719	4.337994
C	1.500822	14.143703	5.012504

C	3.264338	13.028273	6.112973
C	1.061197	13.414771	9.215989
C	-1.517278	10.770296	3.029435
C	-0.764425	15.441193	8.770077
C	0.402151	9.295124	3.175135
C	2.385532	15.084967	4.498582
C	4.195619	13.928769	5.618848
C	0.945201	14.426739	10.158196
C	-0.768992	9.697634	2.511526
C	0.019479	15.459411	9.935876
C	3.753780	14.979172	4.794184
H	-1.482129	10.049761	7.106735
H	-0.888305	10.523273	8.552833
H	-1.999812	11.431462	7.806559
H	2.674616	10.311356	6.931834
H	1.358483	9.481961	7.424426
H	2.049723	10.572953	8.411585
H	-2.337082	13.173166	4.126808
H	-2.583429	12.164805	5.553859
H	-2.435746	13.751525	6.934893
H	-1.746434	15.191584	6.196318
H	-2.416651	11.120960	2.514476
H	-1.086701	9.196194	1.590424
H	1.022293	8.479282	2.791029
H	1.695564	9.670432	4.858765
H	-0.057175	13.666625	3.656823
H	-0.354631	15.154856	4.555593
H	2.017472	15.895959	3.863384
H	4.465226	15.709340	4.393154
H	5.252172	13.817148	5.879408
H	3.581615	12.224245	6.777303
H	-1.487365	16.238969	8.575165
H	-0.087239	16.274835	10.659920
H	1.578303	14.410300	11.050621
H	1.791975	12.614851	9.349728

$[Ru(TPA)(NH_3)_2]^{4+}$, S = 1, solution

Ru	0.434368	11.922632	6.607698
N	-0.822852	13.311860	5.610903
N	0.000876	10.943582	4.850964
N	1.913949	13.114585	5.838090
N	0.282905	13.363544	8.069285
N	1.704833	10.402575	7.360990
N	-1.091055	10.864051	7.648521
C	-1.831027	12.523156	4.837628

C	-1.115868	11.377788	4.193756
C	-1.480520	14.195835	6.631278
C	-0.589238	14.377247	7.818633
C	0.035245	14.094975	4.647262
C	0.747880	9.932687	4.342671
C	1.470268	14.139529	5.060183
C	3.218108	13.050173	6.199756
C	1.092657	13.389493	9.153522
C	-1.529801	10.769340	3.019433
C	-0.666351	15.464910	8.677919
C	0.372532	9.288135	3.174841
C	2.339230	15.126552	4.613385
C	4.132717	13.997527	5.770623
C	1.042108	14.445050	10.050425
C	-0.785200	9.702143	2.507913
C	0.154120	15.498066	9.808706
C	3.688403	15.054004	4.967095
H	-1.322962	9.982770	7.171896
H	-0.772721	10.601346	8.590100
H	-1.979358	11.356065	7.792022
H	2.610966	10.306819	6.886519
H	1.268063	9.471863	7.332387
H	1.929572	10.557038	8.354025
H	-2.329458	13.163999	4.095591
H	-2.604448	12.149729	5.524503
H	-2.409556	13.705374	6.962273
H	-1.761110	15.155551	6.176876
H	-2.420347	11.142141	2.510996
H	-1.097397	9.210204	1.584001
H	0.991513	8.473408	2.796525
H	1.652130	9.645031	4.876162
H	-0.012828	13.595239	3.666778
H	-0.380613	15.102663	4.515054
H	1.955596	15.940853	3.996745
H	4.386509	15.822679	4.628427
H	5.175870	13.912311	6.077582
H	3.524260	12.239765	6.860663
H	-1.365173	16.273025	8.456268
H	0.105416	16.347116	10.494094
H	1.702456	14.437838	10.918703
H	1.788583	12.560832	9.291140

[Ru(TPA)(^{cis}NH$_3$)(^{trans}NH$_2$)]$^+$, S = 0, gas phase

Ru	0.335985	11.948480	6.633851
N	-0.894499	13.382403	5.596025

N	0.015572	10.895751	4.879824
N	1.821713	13.095815	5.819896
N	0.276216	13.371161	8.127726
N	1.513406	10.542410	7.616101
N	-1.087757	10.683201	7.648706
C	-1.805277	12.525377	4.804433
C	-1.030529	11.391120	4.159111
C	-1.537551	14.149073	6.684951
C	-0.595257	14.380800	7.850025
C	-0.032066	14.218570	4.716167
C	0.726555	9.873098	4.373519
C	1.430146	14.165927	5.088864
C	3.130602	12.940846	6.106643
C	1.079244	13.482692	9.201122
C	-1.372467	10.864801	2.917217
C	-0.656418	15.526736	8.638462
C	0.427258	9.288674	3.143134
C	2.343270	15.120939	4.642691
C	4.096189	13.845902	5.679194
C	1.053967	14.592643	10.043706
C	-0.640990	9.789329	2.399809
C	0.175471	15.637737	9.757656
C	3.698491	14.962045	4.938160
H	-1.704885	10.067534	7.115518
H	-0.274419	10.125298	8.010047
H	-1.619010	11.049282	8.440709
H	2.100697	9.958423	7.014063
H	2.162523	10.899819	8.322124
H	-2.363890	13.093891	4.037729
H	-2.542393	12.102111	5.502855
H	-2.382157	13.543599	7.048736
H	-1.951202	15.109945	6.329339
H	-2.208164	11.292808	2.359761
H	-0.899719	9.358725	1.430308
H	1.031284	8.455554	2.779752
H	1.558148	9.521415	4.986211
H	-0.124546	13.831942	3.688959
H	-0.391497	15.260499	4.689259
H	1.992301	15.978220	4.064265
H	4.430308	15.697157	4.597092
H	5.144222	13.677030	5.932471
H	3.366100	12.057520	6.701497
H	-1.355857	16.324479	8.380341
H	0.137608	16.526903	10.390218
H	1.722870	14.632032	10.905157
H	1.760204	12.648585	9.376721

[Ru(TPA)(^{cis}NH$_3$)(^{trans}NH$_2$)]$^+$, *S* = 0, solution

Ru	0.346015	11.925317	6.644705
N	-0.889349	13.346826	5.620181
N	0.026285	10.875243	4.888893
N	1.835156	13.074214	5.829323
N	0.292734	13.353516	8.134243
N	1.568609	10.521904	7.624088
N	-1.171595	10.758849	7.605783
C	-1.800681	12.500618	4.815445
C	-1.024858	11.367510	4.173776
C	-1.546049	14.104887	6.709374
C	-0.598473	14.348536	7.864638
C	-0.035621	14.200570	4.748018
C	0.745192	9.860833	4.375241
C	1.427655	14.147565	5.109526
C	3.148039	12.930788	6.105398
C	1.119656	13.487582	9.187424
C	-1.367955	10.841221	2.931188
C	-0.660013	15.500065	8.646326
C	0.446632	9.278882	3.143987
C	2.329954	15.115633	4.666690
C	4.102701	13.848787	5.680488
C	1.096944	14.604638	10.020522
C	-0.630873	9.773497	2.407600
C	0.193559	15.632926	9.746265
C	3.688694	14.968006	4.951498
H	-1.815745	10.229860	7.013015
H	-0.558092	10.083601	8.081323
H	-1.739493	11.222587	8.318615
H	2.198863	10.065560	6.953865
H	2.230911	10.985872	8.255851
H	-2.341112	13.079243	4.046080
H	-2.551404	12.078094	5.499225
H	-2.378103	13.486243	7.078810
H	-1.971046	15.058440	6.352779
H	-2.207135	11.271078	2.380509
H	-0.889599	9.345068	1.436679
H	1.058313	8.453957	2.773757
H	1.586344	9.515653	4.979545
H	-0.131320	13.834392	3.713934
H	-0.397371	15.240859	4.743469
H	1.961622	15.973837	4.100612
H	4.410827	15.714494	4.613198
H	5.153820	13.687965	5.927725
H	3.404044	12.047797	6.692120
H	-1.375260	16.284071	8.389753

H	0.156936	16.528026	10.371167
H	1.787975	14.663012	10.863681
H	1.823468	12.670179	9.353795

$[Ru(TPA)(^{cis}NH_3)(^{trans}NH_2)]^{2+}$, S = 1/2, gas phase

Ru	0.424373	11.911276	6.653108
N	-0.841387	13.326188	5.627773
N	0.030477	10.879872	4.870061
N	1.906545	13.120930	5.823254
N	0.329211	13.371348	8.136788
N	1.644533	10.638802	7.452196
N	-1.149859	10.769222	7.608757
C	-1.796526	12.493054	4.848519
C	-1.045584	11.356140	4.186983
C	-1.477694	14.150990	6.690400
C	-0.544067	14.375447	7.860738
C	0.003036	14.136262	4.695504
C	0.737955	9.853163	4.362659
C	1.459505	14.154950	5.073426
C	3.218440	13.058140	6.123583
C	1.134702	13.463955	9.212192
C	-1.430246	10.803531	2.968818
C	-0.615942	15.509147	8.666456
C	0.397103	9.247312	3.155003
C	2.319693	15.147507	4.604247
C	4.136718	14.001498	5.675552
C	1.097697	14.562372	10.067576
C	-0.704674	9.727272	2.445917
C	0.212719	15.605250	9.788881
C	3.680813	15.070614	4.901576
H	-1.601158	10.085709	6.992275
H	-0.768628	10.221663	8.387369
H	-1.906194	11.318967	8.026507
H	2.652461	10.601890	7.296332
H	1.375504	9.821363	8.004975
H	-2.348554	13.089681	4.103122
H	-2.544817	12.088960	5.547762
H	-2.363740	13.603060	7.050031
H	-1.845238	15.109882	6.290976
H	-2.291224	11.210182	2.434378
H	-0.995003	9.276643	1.494174
H	0.994129	8.413162	2.782260
H	1.589648	9.519686	4.956960
H	-0.072491	13.683473	3.694483
H	-0.395611	15.158604	4.605439

H	1.923463	15.970559	4.005914
H	4.372164	15.835072	4.540300
H	5.189948	13.898527	5.942043
H	3.542359	12.231634	6.754808
H	-1.318947	16.307815	8.421071
H	0.166943	16.485390	10.434192
H	1.761033	14.595392	10.933664
H	1.816215	12.626741	9.371129

[Ru(TPA)(^{cis}NH$_3$)(^{trans}NH$_2$)]$^{2+}$, S = 1/2, solution

Ru	0.422050	11.909889	6.645733
N	-0.842577	13.324776	5.625315
N	0.032080	10.878313	4.876427
N	1.904605	13.119238	5.814316
N	0.334449	13.364476	8.123777
N	1.632966	10.634730	7.448113
N	-1.143848	10.793562	7.585603
C	-1.795398	12.490258	4.848184
C	-1.042823	11.353405	4.194605
C	-1.479679	14.145380	6.688100
C	-0.542838	14.364470	7.853385
C	-0.002236	14.139628	4.697881
C	0.737250	9.846562	4.379822
C	1.451962	14.159144	5.077678
C	3.214431	13.055070	6.118945
C	1.149450	13.455209	9.190591
C	-1.428064	10.794448	2.979086
C	-0.607603	15.499073	8.659687
C	0.394602	9.234646	3.176680
C	2.308092	15.165425	4.629045
C	4.127581	14.012223	5.690390
C	1.119522	14.553807	10.044846
C	-0.705555	9.714736	2.464177
C	0.230523	15.595687	9.772887
C	3.666903	15.091763	4.933328
H	-1.583849	10.101857	6.971362
H	-0.780513	10.260785	8.381577
H	-1.908681	11.356173	7.968051
H	2.638435	10.582894	7.281754
H	1.336689	9.822870	7.994320
H	-2.339558	13.084605	4.096904
H	-2.539731	12.078788	5.545745
H	-2.358450	13.593327	7.055655
H	-1.841605	15.106287	6.291495
H	-2.287186	11.207531	2.447599

H	-0.995993	9.260088	1.514506
H	0.989519	8.396546	2.810116
H	1.587019	9.516459	4.978972
H	-0.070881	13.691605	3.694527
H	-0.401176	15.161268	4.613563
H	1.902467	15.994523	4.046336
H	4.354417	15.868531	4.591610
H	5.179717	13.911933	5.961534
H	3.536623	12.217183	6.736036
H	-1.312606	16.294284	8.411045
H	0.192093	16.477476	10.416257
H	1.793283	14.588609	10.902496
H	1.833921	12.618757	9.340070

$[Ru(TPA)(^{cis}NH_3)(^{trans}NH_2)]^{3+}$, S = 1, gas phase

Ru	0.469252	11.969508	6.600660
N	-0.815660	13.361658	5.609166
N	0.032412	10.887563	4.882464
N	1.923522	13.165546	5.788299
N	0.326837	13.351723	8.134544
N	1.695899	10.679154	7.406917
N	-1.098180	10.854079	7.621445
C	-1.779987	12.529418	4.824562
C	-1.039316	11.378133	4.183013
C	-1.462321	14.189422	6.676188
C	-0.561104	14.359775	7.879032
C	0.014344	14.184120	4.661459
C	0.715612	9.818866	4.406545
C	1.473715	14.192584	5.014730
C	3.245524	13.087219	6.086801
C	1.099757	13.392763	9.246695
C	-1.431799	10.799085	2.983052
C	-0.675038	15.440664	8.746239
C	0.363285	9.196669	3.214649
C	2.346146	15.153612	4.513151
C	4.165944	14.004531	5.604101
C	1.014895	14.439630	10.155774
C	-0.727321	9.690350	2.490968
C	0.117350	15.482996	9.901526
C	3.711544	15.059284	4.801762
H	-1.255601	9.931855	7.197079
H	-0.847498	10.668428	8.600070
H	-2.021490	11.297080	7.667984
H	2.671207	10.531490	7.129817
H	1.448890	9.972850	8.108567

H	-2.311704	13.133870	4.072547
H	-2.548841	12.142279	5.511633
H	-2.383346	13.673895	6.994686
H	-1.781528	15.165803	6.280983
H	-2.285023	11.204405	2.434008
H	-1.028741	9.220422	1.550555
H	0.937616	8.336673	2.862774
H	1.557344	9.468460	5.005177
H	-0.084082	13.747075	3.654729
H	-0.384660	15.207467	4.595474
H	1.962517	15.968821	3.895101
H	4.411623	15.803373	4.411762
H	5.222097	13.898683	5.861447
H	3.564027	12.278024	6.742195
H	-1.384091	16.243346	8.529846
H	0.032710	16.324263	10.595201
H	1.647469	14.437211	11.046315
H	1.795591	12.564174	9.388439

[Ru(TPA)(^{cis}NH$_3$)(^{trans}NH$_2$)]$^{3+}$, S = 1, solution

Ru	0.457537	11.950915	6.579174
N	-0.816183	13.350291	5.587894
N	0.015291	10.869190	4.873722
N	1.915113	13.174977	5.788905
N	0.334594	13.334915	8.105828
N	1.687170	10.671366	7.378613
N	-1.060401	10.871275	7.619786
C	-1.782783	12.525270	4.808327
C	-1.051085	11.364910	4.181195
C	-1.446023	14.189413	6.648324
C	-0.539167	14.346414	7.844773
C	0.015835	14.160090	4.639123
C	0.687317	9.792972	4.414059
C	1.460844	14.202435	5.028774
C	3.222274	13.113275	6.129479
C	1.111823	13.363058	9.208783
C	-1.452312	10.781126	2.986004
C	-0.629981	15.436975	8.703740
C	0.325020	9.166278	3.228664
C	2.321910	15.199048	4.579195
C	4.132109	14.066132	5.698188
C	1.049844	14.418863	10.107800
C	-0.760381	9.665955	2.504915
C	0.170385	15.473722	9.848059
C	3.675064	15.128717	4.911975

H	-1.273183	9.980335	7.157233
H	-0.743926	10.630897	8.565257
H	-1.958985	11.345910	7.747553
H	2.612497	10.438136	7.006118
H	1.464552	10.060612	8.171008
H	-2.296601	13.128791	4.045177
H	-2.553192	12.139840	5.492309
H	-2.366525	13.685031	6.982132
H	-1.742955	15.170759	6.252280
H	-2.300458	11.201730	2.443454
H	-1.066666	9.193809	1.568834
H	0.890555	8.299321	2.884491
H	1.525069	9.443360	5.017647
H	-0.040060	13.688391	3.645418
H	-0.400007	15.171983	4.534556
H	1.926748	16.017924	3.975984
H	4.366470	15.901606	4.569216
H	5.179724	13.979229	5.989197
H	3.530259	12.291624	6.774754
H	-1.326063	16.244930	8.472786
H	0.108468	16.323708	10.531257
H	1.689608	14.412392	10.991307
H	1.790659	12.520398	9.346862

[Ru(TPA)(NH_2)$_2$], S = 0, gas phase

Ru	0.311694	11.924465	6.622737
N	-0.940054	13.378509	5.624971
N	0.001711	10.902146	4.895174
N	1.817450	13.055807	5.751488
N	0.332209	13.337961	8.101166
N	1.674391	10.519131	7.329244
N	-1.385122	11.028065	7.445960
C	-1.835166	12.489324	4.848218
C	-1.032459	11.402667	4.161687
C	-1.575086	14.055145	6.771603
C	-0.570780	14.336642	7.867944
C	-0.087582	14.251866	4.787511
C	0.762467	9.911896	4.394706
C	1.397525	14.167353	5.103873
C	3.133497	12.860007	5.961353
C	1.211489	13.484720	9.111061
C	-1.317229	10.907510	2.893830
C	-0.581826	15.500195	8.631208
C	0.516172	9.364427	3.135245
C	2.298781	15.146345	4.686703

C	4.087337	13.789769	5.555164
C	1.242361	14.618660	9.921315
C	-0.538109	9.865365	2.369760
C	0.333489	15.650458	9.680870
C	3.666059	14.960354	4.915902
H	-1.354649	11.095970	8.469587
H	-1.349235	10.012394	7.296397
H	2.065550	10.766147	8.248055
H	1.165289	9.652819	7.550741
H	-2.465683	13.037129	4.121995
H	-2.463514	11.986496	5.603406
H	-2.298083	13.327743	7.175201
H	-2.105966	14.983050	6.482301
H	-2.142982	11.334962	2.320574
H	-0.750707	9.461545	1.377191
H	1.150690	8.556877	2.763919
H	1.571356	9.591064	5.060200
H	-0.214790	13.939872	3.738094
H	-0.426537	15.301201	4.842071
H	1.931981	16.044453	4.183455
H	4.388075	15.716913	4.599874
H	5.146016	13.599265	5.744791
H	3.360266	11.912102	6.464651
H	-1.308484	16.283470	8.404002
H	0.336389	16.556047	10.291401
H	1.976559	14.685496	10.726931
H	1.905301	12.654309	9.254408

[Ru(TPA)(NH_2)$_2$], S = 0, solution

Ru	0.302426	11.884198	6.625172
N	-0.895451	13.332843	5.599458
N	-0.038594	10.856408	4.873449
N	1.842048	13.071158	5.845151
N	0.312498	13.314133	8.103848
N	1.557177	10.468375	7.574124
N	-1.332241	10.924287	7.503751
C	-1.846728	12.502902	4.829438
C	-1.107589	11.348922	4.181046
C	-1.500500	14.132815	6.684095
C	-0.524332	14.354082	7.819802
C	-0.020657	14.132158	4.700612
C	0.660978	9.835455	4.341801
C	1.420144	14.156174	5.146277
C	3.141403	12.995797	6.199396
C	1.154739	13.431366	9.149123

C	-1.484474	10.818504	2.950296
C	-0.504837	15.530131	8.565596
C	0.328561	9.250828	3.120713
C	2.284962	15.196394	4.810326
C	4.064884	13.988123	5.881590
C	1.212781	14.573788	9.945880
C	-0.766945	9.745072	2.409607
C	0.371939	15.648552	9.650931
C	3.632538	15.116466	5.177617
H	-0.900677	10.185787	8.074241
H	-1.820564	10.370672	6.786939
H	2.231366	10.111743	6.884019
H	2.184340	10.948495	8.231159
H	-2.396669	13.083307	4.066865
H	-2.579283	12.104018	5.544733
H	-2.341991	13.546665	7.079210
H	-1.893638	15.097606	6.319659
H	-2.335435	11.251330	2.420092
H	-1.053375	9.313660	1.447938
H	0.927806	8.421886	2.738757
H	1.516105	9.489499	4.925794
H	-0.046022	13.659528	3.706373
H	-0.411126	15.154981	4.574285
H	1.899781	16.060788	4.264941
H	4.326831	15.920447	4.923472
H	5.106073	13.876931	6.191787
H	3.415766	12.101433	6.762152
H	-1.176655	16.346757	8.293121
H	0.398514	16.563221	10.247182
H	1.916944	14.613585	10.779566
H	1.807237	12.576836	9.336554

$[Ru(TPA)(NH_2)_2]^+$, S = 1/2, gas phase

Ru	0.348140	11.864006	6.656324
N	-0.873116	13.343606	5.592211
N	0.019129	10.822395	4.888908
N	1.876070	13.127267	5.763429
N	0.323788	13.321275	8.140035
N	1.708396	10.677073	7.419727
N	-1.277083	11.059402	7.409956
C	-1.785441	12.476576	4.805024
C	-1.009408	11.341787	4.163302
C	-1.516713	14.075819	6.708427
C	-0.558032	14.318545	7.858006
C	-0.035151	14.201047	4.719808

C	0.731727	9.801606	4.386114
C	1.429837	14.204584	5.093035
C	3.185270	13.003775	6.037458
C	1.150540	13.452087	9.189652
C	-1.338123	10.826665	2.911464
C	-0.611356	15.474300	8.634695
C	0.444341	9.230336	3.147673
C	2.295463	15.221941	4.688711
C	4.111576	13.968249	5.648476
C	1.136484	14.573433	10.016924
C	-0.611107	9.748471	2.397292
C	0.241183	15.605176	9.734566
C	3.658189	15.102043	4.969576
H	-1.549702	10.125455	7.099682
H	-1.411640	11.102952	8.420906
H	1.701974	9.688655	7.160771
H	1.895478	10.705532	8.424143
H	-2.348453	13.044419	4.044397
H	-2.493311	12.037965	5.522345
H	-2.317302	13.422680	7.085304
H	-1.965692	15.026137	6.372599
H	-2.160696	11.267904	2.345113
H	-0.861092	9.328780	1.420531
H	1.046925	8.396894	2.782916
H	1.561796	9.449381	5.001567
H	-0.105785	13.807144	3.693269
H	-0.432633	15.228895	4.685682
H	1.906021	16.091869	4.155555
H	4.355895	15.884357	4.662430
H	5.168633	13.832791	5.883995
H	3.443428	12.095568	6.590990
H	-1.320357	16.264301	8.379340
H	0.208736	16.502889	10.355614
H	1.824296	14.631669	10.862125
H	1.848936	12.630407	9.357872

[Ru(TPA)(NH$_2$)$_2$]$^+$, S = 1/2, solution

Ru	0.360153	11.839218	6.659756
N	-0.847494	13.295513	5.611836
N	0.004899	10.782568	4.895900
N	1.897474	13.095615	5.805865
N	0.320305	13.289926	8.153559
N	1.735923	10.621331	7.451230
N	-1.238445	10.986010	7.431583
C	-1.777852	12.465957	4.804740

C	-1.028510	11.305527	4.180875
C	-1.490229	14.076105	6.697922
C	-0.548841	14.295159	7.864059
C	-0.004339	14.136928	4.720942
C	0.678601	9.730020	4.403583
C	1.443034	14.167950	5.125510
C	3.208022	13.014215	6.098625
C	1.124468	13.402523	9.223616
C	-1.392785	10.774502	2.944993
C	-0.607338	15.446810	8.647735
C	0.356279	9.142263	3.181997
C	2.290606	15.207851	4.739620
C	4.117090	14.000777	5.728528
C	1.103381	14.517509	10.058583
C	-0.698562	9.672948	2.438033
C	0.225161	15.561596	9.763715
C	3.649682	15.125289	5.043627
H	-1.867265	10.417393	6.864215
H	-1.770754	11.444185	8.171051
H	2.319975	10.110318	6.785163
H	2.377334	11.025374	8.136575
H	-2.287084	13.060321	4.029045
H	-2.539593	12.065855	5.486703
H	-2.340683	13.481983	7.061953
H	-1.880199	15.038460	6.330446
H	-2.215680	11.228000	2.389188
H	-0.973966	9.242525	1.472649
H	0.931783	8.286068	2.825909
H	1.502070	9.353342	5.012656
H	-0.044450	13.704644	3.708910
H	-0.417840	15.154015	4.645545
H	1.880687	16.065960	4.203551
H	4.332356	15.926724	4.752297
H	5.171440	13.887983	5.986979
H	3.511064	12.127331	6.654999
H	-1.303929	16.243073	8.378975
H	0.190939	16.456551	10.388931
H	1.774378	14.561467	10.918188
H	1.808793	12.572428	9.407578

$[Ru(TPA)(NH_2)_2]^{2+}$, $S = 1$, gas phase

Ru	0.390865	11.886328	6.642357
N	-0.825255	13.321928	5.600402
N	-0.004125	10.846238	4.864463
N	1.938178	13.132870	5.798474

N	0.318316	13.348996	8.128531
N	1.650185	10.634824	7.412036
N	-1.100414	10.970740	7.459199
C	-1.791504	12.500609	4.811925
C	-1.057980	11.346685	4.165563
C	-1.469003	14.142877	6.667633
C	-0.544819	14.358991	7.845110
C	0.026348	14.130585	4.673073
C	0.676764	9.790194	4.382242
C	1.477395	14.174042	5.070904
C	3.246543	13.087478	6.111143
C	1.108428	13.424093	9.215825
C	-1.443274	10.790317	2.949065
C	-0.618898	15.487508	8.658015
C	0.332832	9.180314	3.178336
C	2.320646	15.195978	4.634676
C	4.151659	14.058412	5.695044
C	1.067429	14.516594	10.078066
C	-0.744111	9.686604	2.449165
C	0.194427	15.568031	9.792698
C	3.680379	15.137286	4.943921
H	-1.553099	10.135486	7.081076
H	-1.440492	11.169237	8.402533
H	2.644970	10.586931	7.187987
H	1.380554	9.858059	8.016587
H	-2.318334	13.111693	4.061669
H	-2.542134	12.106913	5.512347
H	-2.346476	13.580167	7.021677
H	-1.832666	15.101735	6.266781
H	-2.286628	11.214075	2.400168
H	-1.036435	9.232516	1.499610
H	0.906880	8.322053	2.824703
H	1.508690	9.433977	4.990800
H	-0.028171	13.663392	3.677262
H	-0.386883	15.145161	4.567138
H	1.914765	16.026180	4.052915
H	4.359759	15.924260	4.608771
H	5.204604	13.970483	5.968140
H	3.582349	12.253511	6.728015
H	-1.313730	16.292483	8.410271
H	0.145135	16.443242	10.444586
H	1.716328	14.537678	10.955400
H	1.780059	12.580355	9.381641

[Ru(TPA)(NH$_2$)$_2$]$^{2+}$, S = 1, solution

Ru	0.383277	11.876932	6.640811
N	-0.832667	13.308599	5.604028
N	-0.004918	10.837920	4.871917
N	1.926906	13.121183	5.798095
N	0.325268	13.340254	8.116631
N	1.638521	10.620705	7.403937
N	-1.115931	10.980778	7.456447
C	-1.794413	12.489122	4.813169
C	-1.057136	11.336895	4.173482
C	-1.477464	14.124302	6.671522
C	-0.543405	14.344488	7.837185
C	0.014436	14.125416	4.682581
C	0.685271	9.788451	4.392284
C	1.460748	14.169934	5.086701
C	3.231674	13.075826	6.120962
C	1.140001	13.425873	9.183052
C	-1.435046	10.782894	2.953271
C	-0.599814	15.483365	8.637602
C	0.348430	9.181934	3.185464
C	2.298369	15.208306	4.678670
C	4.129798	14.064093	5.731859
C	1.117975	14.529403	10.030840
C	-0.728588	9.685887	2.454186
C	0.238012	15.576783	9.751227
C	3.654189	15.154395	5.000315
H	-1.602503	10.172820	7.062455
H	-1.506453	11.246968	8.362351
H	2.627155	10.561225	7.155687
H	1.364980	9.840780	8.001947
H	-2.312199	13.099260	4.057568
H	-2.547880	12.091108	5.507652
H	-2.345351	13.555266	7.037249
H	-1.844917	15.081292	6.272726
H	-2.275382	11.214001	2.406494
H	-1.013295	9.235475	1.500777
H	0.930364	8.329716	2.831024
H	1.518728	9.439774	5.003407
H	-0.030276	13.663070	3.684381
H	-0.401873	15.137996	4.582480
H	1.881910	16.045078	4.115086
H	4.328430	15.956168	4.691293
H	5.180083	13.979594	6.014972
H	3.566665	12.230574	6.722420
H	-1.297188	16.283555	8.384013
H	0.206377	16.462100	10.390107

H	1.790599	14.562883	10.889336
H	1.818593	12.585669	9.338279

[Ru(TPA)(^{trans}NH$_3$)(^{cis}NH$_2$)]$^+$, S = 0, gas phase

Ru	0.408687	11.903235	6.576024
N	-0.829373	13.290924	5.597164
N	0.064944	10.844522	4.829271
N	1.913776	13.129606	5.765898
N	0.377058	13.312625	8.083627
N	1.501444	10.377759	7.629760
N	-1.065409	10.768680	7.516268
C	-1.771328	12.442228	4.822244
C	-1.012609	11.322301	4.145413
C	-1.476797	14.051415	6.695511
C	-0.513965	14.310747	7.830311
C	-0.009195	14.146597	4.691672
C	0.763279	9.825237	4.300072
C	1.444636	14.205941	5.090801
C	3.224801	13.079595	6.068042
C	1.207648	13.441909	9.133317
C	-1.398552	10.783830	2.921589
C	-0.569793	15.458266	8.616992
C	0.422822	9.227079	3.087827
C	2.273634	15.264195	4.721683
C	4.116715	14.090433	5.718958
C	1.192179	14.552561	9.974437
C	-0.678835	9.711637	2.383230
C	0.290944	15.584624	9.711716
C	3.633539	15.209184	5.036699
H	-1.732244	10.270485	6.921318
H	-1.630095	11.241114	8.226372
H	1.985614	9.645791	7.106831
H	0.575504	10.008048	7.967349
H	2.089331	10.604004	8.434020
H	-2.356577	13.026408	4.090199
H	-2.465995	11.998033	5.548643
H	-2.281683	13.410332	7.084639
H	-1.929952	14.991154	6.335966
H	-2.260999	11.199196	2.396469
H	-0.971964	9.270083	1.428637
H	1.020253	8.396704	2.707844
H	1.627808	9.485848	4.873119
H	-0.052699	13.698091	3.686785
H	-0.440900	15.157152	4.606771
H	1.856722	16.121534	4.189041

H	4.301899	16.025551	4.755569
H	5.171110	13.999922	5.985539
H	3.551517	12.192162	6.613256
H	-1.288322	16.244267	8.376278
H	0.258220	16.474342	10.343675
H	1.884843	14.602969	10.816238
H	1.911868	12.624590	9.295538

[Ru(TPA)(^{trans}NH$_3$)(^{cis}NH$_2$)]$^+$, S = 0, solution

Ru	0.399360	11.888429	6.582686
N	-0.835041	13.282561	5.603593
N	0.046504	10.831622	4.838053
N	1.907492	13.114620	5.786792
N	0.361735	13.297693	8.093714
N	1.603373	10.425848	7.581041
N	-1.114261	10.764169	7.509538
C	-1.780002	12.450598	4.817223
C	-1.030615	11.315332	4.156015
C	-1.480779	14.058812	6.691306
C	-0.523184	14.300751	7.834034
C	-0.007233	14.134755	4.699996
C	0.733566	9.800363	4.312795
C	1.441449	14.190498	5.107945
C	3.216808	13.065053	6.101555
C	1.190171	13.423087	9.147520
C	-1.425389	10.773168	2.935988
C	-0.573642	15.451062	8.618439
C	0.382925	9.200632	3.104114
C	2.272849	15.250283	4.745802
C	4.109581	14.077215	5.758140
C	1.178353	14.537047	9.984701
C	-0.716960	9.692710	2.399935
C	0.284226	15.574804	9.715627
C	3.630257	15.196123	5.071390
H	-1.744519	10.346917	6.814730
H	-1.745528	11.365431	8.051393
H	2.109930	9.755595	6.998058
H	0.846452	9.915156	8.056229
H	2.267984	10.735139	8.293729
H	-2.335429	13.043574	4.071088
H	-2.504927	12.025229	5.524890
H	-2.309779	13.444881	7.073270
H	-1.903521	15.008074	6.323754
H	-2.284163	11.199816	2.413835
H	-1.016172	9.251254	1.446730

H	0.973127	8.363387	2.726898
H	1.595216	9.450634	4.884364
H	-0.044115	13.687820	3.694357
H	-0.435932	15.145127	4.613966
H	1.853832	16.106350	4.212866
H	4.299952	16.014297	4.797181
H	5.161517	13.987756	6.035952
H	3.544425	12.181957	6.653357
H	-1.284779	16.240241	8.366561
H	0.257012	16.468349	10.343137
H	1.871327	14.585177	10.826834
H	1.890056	12.603513	9.317501

[Ru(TPA)(^{trans}NH$_3$)(^{cis}NH$_2$)]$^{2+}$, S = 1/2, gas phase

Ru	0.348602	11.890892	6.612062
N	-0.831153	13.298079	5.610163
N	-0.008008	10.823704	4.841937
N	1.921815	13.100973	5.793895
N	0.317158	13.334604	8.129692
N	1.698612	10.447410	7.515624
N	-1.154216	10.981085	7.422071
C	-1.792398	12.488146	4.800061
C	-1.057990	11.339277	4.144408
C	-1.488695	14.106430	6.680068
C	-0.561276	14.334887	7.851768
C	0.020050	14.128520	4.695651
C	0.667364	9.781662	4.326722
C	1.470468	14.165009	5.094045
C	3.231432	13.031743	6.096139
C	1.114228	13.446620	9.206037
C	-1.442256	10.811302	2.914274
C	-0.644889	15.471244	8.653730
C	0.328926	9.195865	3.108914
C	2.320807	15.192282	4.685761
C	4.144946	14.006155	5.706312
C	1.071566	14.544953	10.061101
C	-0.746632	9.717775	2.389236
C	0.177383	15.578712	9.779016
C	3.680697	15.112606	4.991120
H	-1.649288	10.181079	7.022351
H	-1.514491	11.191115	8.354640
H	2.501661	10.186446	6.935627
H	1.178799	9.581288	7.685375
H	2.084954	10.713017	8.426088
H	-2.310022	13.111577	4.053654

H	-2.548012	12.089662	5.491185
H	-2.351868	13.522806	7.033669
H	-1.871960	15.058882	6.282243
H	-2.282366	11.250800	2.372718
H	-1.037696	9.286234	1.428962
H	0.905412	8.347552	2.735994
H	1.509482	9.404728	4.909853
H	-0.032305	13.676281	3.692981
H	-0.398879	15.142115	4.607497
H	1.921588	16.041800	4.127807
H	4.366466	15.902918	4.677866
H	5.198868	13.899577	5.968693
H	3.561719	12.169073	6.677149
H	-1.353950	16.262858	8.403461
H	0.121120	16.459079	10.423057
H	1.733501	14.584607	10.927902
H	1.814235	12.629482	9.390044

$[Ru(TPA)(^{trans}NH_3)(^{cis}NH_2)]^{2+}$, S = 1/2, solution

Ru	0.340487	11.878303	6.611036
N	-0.839557	13.290511	5.607055
N	-0.013503	10.814199	4.850341
N	1.908418	13.095587	5.801441
N	0.322439	13.319732	8.117400
N	1.679442	10.449019	7.490026
N	-1.169717	10.984622	7.416571
C	-1.797022	12.480803	4.798510
C	-1.061225	11.329633	4.152108
C	-1.491907	14.095588	6.678821
C	-0.554069	14.321023	7.841130
C	0.009341	14.122370	4.696921
C	0.667089	9.772463	4.342836
C	1.454422	14.160265	5.105506
C	3.212104	13.035430	6.128613
C	1.144570	13.435254	9.173720
C	-1.440656	10.801629	2.920448
C	-0.613163	15.468523	8.629485
C	0.331370	9.188496	3.123325
C	2.299377	15.201121	4.720447
C	4.118187	14.026195	5.762381
C	1.125679	14.547051	10.012453
C	-0.741552	9.709978	2.399351
C	0.232954	15.583228	9.735041
C	3.653575	15.133054	5.048508
H	-1.658196	10.180412	7.018528

H	-1.575468	11.250872	8.314850
H	2.532983	10.281211	6.949535
H	1.212904	9.540436	7.556539
H	1.988378	10.668385	8.440667
H	-2.306125	13.100112	4.044131
H	-2.555468	12.081679	5.485851
H	-2.350093	13.511255	7.041665
H	-1.871994	15.050239	6.285652
H	-2.276612	11.249170	2.379905
H	-1.027021	9.280027	1.436748
H	0.911676	8.341554	2.753633
H	1.505544	9.398936	4.932470
H	-0.033832	13.672439	3.693170
H	-0.408993	15.135363	4.610611
H	1.891036	16.050346	4.169557
H	4.334661	15.935690	4.757272
H	5.166972	13.930006	6.047633
H	3.539559	12.174684	6.712695
H	-1.318359	16.260807	8.372394
H	0.199644	16.474607	10.365289
H	1.810476	14.594523	10.860710
H	1.844750	12.616080	9.345641

[Ru(TPA)(^{trans}NH$_3$)(^{cis}NH$_2$)]$^{3+}$, S = 1, gas phase

Ru	0.367053	11.937982	6.590417
N	-0.831966	13.333253	5.604728
N	0.010793	10.863493	4.852318
N	1.907296	13.123513	5.793283
N	0.304106	13.339457	8.130704
N	1.726430	10.484801	7.484311
N	-1.125066	11.024796	7.452371
C	-1.791020	12.519655	4.785642
C	-1.048072	11.371152	4.142915
C	-1.500898	14.141376	6.678109
C	-0.591836	14.338821	7.869035
C	0.014528	14.179132	4.684419
C	0.694994	9.804240	4.361715
C	1.471427	14.174639	5.049504
C	3.226833	13.024099	6.086218
C	1.085593	13.414716	9.230605
C	-1.429916	10.811502	2.929626
C	-0.707695	15.435058	8.717368
C	0.355628	9.198470	3.158407
C	2.350316	15.146164	4.578130
C	4.158071	13.947359	5.634197

C	1.005401	14.475442	10.124245
C	-0.726737	9.706221	2.430419
C	0.094547	15.505380	9.863693
C	3.713688	15.032164	4.866833
H	-1.604504	10.199734	7.076501
H	-1.476807	11.247945	8.388983
H	2.559730	10.279931	6.921954
H	1.259418	9.580498	7.617491
H	2.078601	10.734608	8.414710
H	-2.296208	13.146339	4.034667
H	-2.566014	12.128932	5.462276
H	-2.390517	13.578997	7.003258
H	-1.855240	15.103832	6.280399
H	-2.273691	11.229894	2.375888
H	-1.019001	9.251238	1.479831
H	0.931788	8.343119	2.798647
H	1.537489	9.438055	4.950111
H	-0.078923	13.767125	3.667051
H	-0.387360	15.202220	4.646440
H	1.974626	15.982134	3.983406
H	4.420754	15.782372	4.502208
H	5.213563	13.823631	5.885957
H	3.542661	12.186634	6.708386
H	-1.425974	16.227186	8.493349
H	0.009600	16.357217	10.544121
H	1.648910	14.493915	11.006571
H	1.797114	12.604471	9.397204

[Ru(TPA)(^{trans}NH$_3$)(^{cis}NH$_2$)]$^{3+}$, S = 1, solution

Ru	0.336090	11.900161	6.600602
N	-0.856162	13.305548	5.609704
N	0.013715	10.861375	4.846619
N	1.882124	13.102352	5.814553
N	0.320574	13.329483	8.111723
N	1.638907	10.450328	7.479352
N	-1.155937	11.024416	7.462321
C	-1.811235	12.488545	4.801967
C	-1.056726	11.353966	4.154756
C	-1.507980	14.114662	6.683115
C	-0.570732	14.325405	7.846262
C	-0.008959	14.141613	4.692407
C	0.733845	9.838572	4.342943
C	1.439033	14.150923	5.080172
C	3.189833	13.016539	6.139220
C	1.145673	13.422420	9.172162

C	-1.428792	10.803521	2.934705
C	-0.640879	15.451251	8.659950
C	0.403815	9.244854	3.131596
C	2.307834	15.148302	4.644798
C	4.110454	13.966846	5.721953
C	1.111204	14.515180	10.029036
C	-0.696087	9.730328	2.419679
C	0.206145	15.546527	9.767029
C	3.662028	15.053668	4.964664
H	-1.612986	10.182590	7.097140
H	-1.521299	11.286155	8.382571
H	2.531050	10.328617	6.990029
H	1.196759	9.525508	7.479694
H	1.877004	10.636822	8.457728
H	-2.321778	13.107940	4.050290
H	-2.573261	12.080619	5.482133
H	-2.379867	13.546946	7.042134
H	-1.870506	15.073390	6.287085
H	-2.281449	11.220081	2.396255
H	-0.976948	9.284916	1.462771
H	1.006493	8.416691	2.756551
H	1.589498	9.497552	4.925455
H	-0.076434	13.712480	3.680657
H	-0.415173	15.160686	4.634492
H	1.917974	15.984979	4.062907
H	4.360637	15.825432	4.634283
H	5.159313	13.859264	6.001186
H	3.500847	12.177191	6.760458
H	-1.353289	16.242534	8.421713
H	0.164196	16.424829	10.414896
H	1.792770	14.554756	10.879758
H	1.852172	12.606149	9.327148

[Ru(TPA)(MeCN)(NH_3)]$^{2+}$ (2^{2+}), S = 0, gas phase

Ru	2.327740	-0.940649	15.550610
N	2.362331	-0.863937	17.557101
N	4.293029	-1.572103	15.230067
N	2.294722	-1.108498	13.450509
N	0.361070	-0.318654	15.255984
N	1.547826	-2.850248	15.426770
N	3.033603	1.119049	15.649897
C	2.396408	-0.800993	18.716685
C	4.680956	-1.431917	13.932069
C	5.139252	-2.136780	16.109044
C	3.677256	-0.768740	13.009518

C	1.249983	-0.150151	12.991564
C	1.975869	-2.522215	13.062392
C	0.070127	-0.136483	13.939197
C	-0.631406	-0.257538	16.161433
C	1.337147	-3.310111	14.172661
C	1.087482	-3.569033	16.470369
C	2.445538	-0.734840	20.170166
C	5.932782	-1.858089	13.497873
C	6.412415	-2.572635	15.744472
H	4.772359	-2.238976	17.131873
H	3.853757	-1.056109	11.959850
H	3.789087	0.324826	13.057109
H	0.926850	-0.365000	11.960193
H	1.704189	0.853401	12.980736
H	2.922842	-3.022519	12.806303
H	1.357850	-2.539047	12.151538
C	-1.232202	0.104605	13.509534
H	-0.345153	-0.424490	17.201234
C	-1.953500	-0.002546	15.802149
C	0.643005	-4.495173	13.930998
H	1.277426	-3.155930	17.460563
C	0.402974	-4.768285	16.305300
H	2.134236	-1.698666	20.603557
H	1.771622	0.053797	20.540516
H	3.469819	-0.510730	20.507594
H	6.214718	-1.743166	12.449108
C	6.818805	-2.430507	14.417698
H	7.067338	-3.020609	16.493849
C	-2.261705	0.178837	14.453427
H	-1.439529	0.237214	12.445747
H	-2.725297	0.044209	16.572296
C	0.168475	-5.240049	15.011449
H	0.483946	-4.833093	12.904963
H	0.055026	-5.317588	17.181736
H	7.807060	-2.767064	14.096806
H	-3.288993	0.370670	14.136152
H	-0.374774	-6.172970	14.846239
H	3.468267	1.527540	14.818606
H	3.734782	1.208354	16.391086
H	2.274902	1.756278	15.909221

$[Ru(TPA)(MeCN)(NH_3)]^{2+}$ (2^{2+}), *S* = 0, solution

Ru	2.327322	-0.953845	15.541541
N	2.361572	-0.879345	17.537690
N	4.287159	-1.569100	15.235001

N	2.298019	-1.124081	13.445046
N	0.372915	-0.330408	15.252385
N	1.538416	-2.858588	15.420521
N	3.025541	1.083132	15.644154
C	2.413868	-0.818071	18.694809
C	4.681113	-1.425654	13.940676
C	5.135114	-2.106325	16.128912
C	3.679221	-0.783028	13.004445
C	1.251070	-0.171512	12.982026
C	1.981481	-2.536824	13.059159
C	0.079873	-0.148439	13.937309
C	-0.610273	-0.259803	16.166596
C	1.329517	-3.315704	14.165319
C	1.058757	-3.563289	16.464763
C	2.477530	-0.754398	20.144930
C	5.945761	-1.829809	13.521351
C	6.419781	-2.517193	15.778067
H	4.760811	-2.204298	17.149634
H	3.860705	-1.091314	11.962632
H	3.792957	0.309637	13.042984
H	0.920108	-0.403037	11.957870
H	1.699102	0.833206	12.963259
H	2.928380	-3.043206	12.816506
H	1.371770	-2.556294	12.143826
C	-1.224553	0.098342	13.515191
H	-0.316665	-0.426375	17.204573
C	-1.932301	0.002401	15.813264
C	0.614096	-4.488326	13.922118
H	1.247137	-3.148922	17.455098
C	0.352624	-4.749827	16.296601
H	2.211465	-1.733508	20.572836
H	1.773391	0.004679	20.519480
H	3.497045	-0.488506	20.464378
H	6.226857	-1.715115	12.472798
C	6.833639	-2.377036	14.452749
H	7.076270	-2.943976	16.538216
C	-2.246662	0.180723	14.464977
H	-1.430422	0.223601	12.450644
H	-2.698126	0.054287	16.588993
C	0.118513	-5.219910	15.001612
H	0.455861	-4.817430	12.893386
H	-0.013787	-5.286655	17.173155
H	7.831038	-2.696323	14.142549
H	-3.275154	0.374864	14.152782
H	-0.444174	-6.141027	14.834883
H	3.319205	1.546801	14.781312

H	3.827040	1.151558	16.277177
H	2.302386	1.685920	16.045628

[Ru(TPA)(MeCN)(NH$_3$)]$^{3+}$, S = 1/2, gas phase

Ru	2.286027	-1.034347	15.526128
N	2.326878	-0.947635	17.589290
N	4.273556	-1.556914	15.224979
N	2.270121	-1.171907	13.437242
N	0.366326	-0.302250	15.238258
N	1.515538	-2.921657	15.390449
N	2.964085	1.031531	15.669148
C	2.386855	-0.859721	18.744152
C	4.665377	-1.430277	13.920517
C	5.134675	-2.054474	16.139297
C	3.653018	-0.808244	12.985959
C	1.217546	-0.221969	12.947705
C	1.979902	-2.596324	13.031596
C	0.063143	-0.130290	13.918193
C	-0.592284	-0.139624	16.176260
C	1.337410	-3.393047	14.128149
C	1.060192	-3.640824	16.445617
C	2.458088	-0.755274	20.190390
C	5.939984	-1.809247	13.516257
C	6.424350	-2.447061	15.795477
H	4.764362	-2.142705	17.161664
H	3.824290	-1.113867	11.941985
H	3.746140	0.288406	13.007584
H	0.872971	-0.498477	11.939919
H	1.680608	0.773832	12.855063
H	2.938512	-3.080935	12.786597
H	1.377686	-2.612806	12.111279
C	-1.227255	0.199378	13.518588
H	-0.292714	-0.298754	17.213337
C	-1.897481	0.202187	15.839634
C	0.681200	-4.596838	13.892473
H	1.224529	-3.213972	17.434007
C	0.413179	-4.856578	16.277829
H	2.193287	-1.721664	20.650559
H	1.757553	0.017302	20.548075
H	3.480637	-0.480731	20.498809
H	6.237580	-1.704671	12.470358
C	6.835970	-2.320903	14.465354
H	7.092632	-2.847417	16.560705
C	-2.222371	0.371139	14.489893
H	-1.456269	0.330083	12.458458

H	-2.646338	0.328393	16.624375
C	0.213059	-5.342438	14.979820
H	0.546123	-4.956111	12.869713
H	0.066438	-5.412366	17.151306
H	7.843343	-2.621022	14.164503
H	-3.241017	0.634187	14.192413
H	-0.301120	-6.293142	14.815501
H	3.237636	1.518246	14.810338
H	3.777601	1.096243	16.290769
H	2.240753	1.618204	16.099837

$[Ru(TPA)(MeCN)(NH_3)]^{3+}$, S = 1/2, solution

Ru	2.300199	-1.025882	15.520200
N	2.338188	-0.947195	17.552878
N	4.275066	-1.542601	15.221831
N	2.276939	-1.171753	13.433790
N	0.382573	-0.307879	15.238696
N	1.518354	-2.917524	15.388336
N	2.967312	0.999454	15.678921
C	2.396745	-0.864266	18.704087
C	4.665340	-1.420481	13.921979
C	5.126721	-2.021818	16.149137
C	3.656245	-0.817307	12.975767
C	1.226616	-0.222779	12.948904
C	1.975547	-2.590850	13.032475
C	0.079359	-0.137793	13.923071
C	-0.564777	-0.157428	16.184291
C	1.331599	-3.379785	14.129115
C	1.059085	-3.622198	16.446212
C	2.463985	-0.768761	20.147451
C	5.944157	-1.796478	13.527467
C	6.418650	-2.409496	15.813659
H	4.748460	-2.095445	17.169396
H	3.827309	-1.150921	11.941781
H	3.756733	0.277762	12.984516
H	0.878126	-0.501670	11.944553
H	1.684349	0.775460	12.873665
H	2.927723	-3.086765	12.788428
H	1.367980	-2.602667	12.117105
C	-1.217183	0.174224	13.528815
H	-0.253840	-0.313781	17.218031
C	-1.874962	0.165627	15.851523
C	0.650466	-4.570218	13.893876
H	1.233904	-3.195198	17.432876
C	0.388481	-4.825039	16.278777

H	2.162200	-1.730345	20.590957
H	1.784752	0.025238	20.494317
H	3.494864	-0.530639	20.452022
H	6.234151	-1.699899	12.479986
C	6.833333	-2.293758	14.484837
H	7.082829	-2.796865	16.587445
C	-2.206119	0.329125	14.504312
H	-1.442896	0.294429	12.468107
H	-2.619607	0.278326	16.640604
C	0.174003	-5.303126	14.982050
H	0.504942	-4.914530	12.868739
H	0.033275	-5.370060	17.154214
H	7.841795	-2.593622	14.191541
H	-3.229670	0.573446	14.211700
H	-0.362539	-6.240505	14.819578
H	3.284366	1.475939	14.830574
H	3.744996	1.055659	16.343792
H	2.222183	1.585287	16.069426

[Ru(TPA)(MeCN)(NH_2)]$^+$, S = 0, gas phase

Ru	4.019025	10.486551	10.808711
N	4.071058	9.542181	12.697740
N	5.877212	9.607058	10.611413
N	3.299752	8.563997	10.221292
N	2.191802	11.127543	11.535076
N	3.950702	11.383229	9.069036
N	4.898537	12.127678	11.713296
C	5.521120	9.450086	13.007874
C	3.389613	8.222205	12.627143
C	3.340842	10.495782	13.577973
C	6.576479	9.386054	9.485271
C	6.315304	9.069318	11.781947
C	3.300091	7.660980	11.226974
C	3.122091	8.133502	8.960215
C	2.085249	10.965704	12.883060
C	1.139994	11.599266	10.844966
C	3.927772	11.960109	8.058206
H	5.770113	12.379790	11.236862
H	4.320316	12.964833	11.584591
H	5.720938	8.764458	13.848278
H	5.826552	10.471618	13.283799
H	3.864462	7.499205	13.309963
H	2.357143	8.360832	12.985771
H	4.005569	11.370259	13.669489
H	3.124752	10.067713	14.571052

H	6.173936	9.840464	8.578326
C	7.741249	8.621574	9.468149
C	7.463309	8.282007	11.831772
C	3.145959	6.295504	10.987767
C	2.944599	6.788420	8.644886
H	3.133520	8.905506	8.187830
C	0.903903	11.268166	13.555436
C	-0.065760	11.934350	11.458425
H	1.285615	11.702857	9.768245
C	3.899074	12.686634	6.795515
H	8.277861	8.472790	8.529605
C	8.192794	8.055085	10.661082
H	7.784994	7.856030	12.784073
H	3.165842	5.591296	11.822253
C	2.963589	5.848165	9.677212
H	2.802896	6.487257	7.605471
H	0.844086	11.119936	14.635369
C	-0.188775	11.765518	12.837806
H	-0.891858	12.317197	10.856883
H	4.559021	13.567280	6.845173
H	2.876700	13.030214	6.570407
H	4.238991	12.042288	5.968786
H	9.097331	7.443612	10.682143
H	2.840691	4.783667	9.466731
H	-1.120980	12.012804	13.350160

$[Ru(TPA)(MeCN)(NH_2)]^+$, S = 0, solution

Ru	4.094871	10.521621	10.856722
N	4.132182	9.534401	12.720663
N	5.963016	9.668707	10.676085
N	3.338353	8.652445	10.223683
N	2.239232	11.108608	11.546584
N	4.014852	11.377031	9.086438
N	4.930642	12.231380	11.736422
C	5.576921	9.490074	13.069673
C	3.528428	8.172780	12.613348
C	3.329286	10.416388	13.608274
C	6.672214	9.452820	9.553636
C	6.401579	9.143367	11.853309
C	3.279188	7.718420	11.196070
C	3.061192	8.295672	8.954505
C	2.089738	10.883082	12.880888
C	1.196105	11.574566	10.836707
C	3.949873	11.871933	8.035716
H	5.803373	12.449500	11.239479

H	4.348280	13.039907	11.485175
H	5.777984	8.800093	13.903546
H	5.848218	10.510238	13.375745
H	4.160798	7.439651	13.135662
H	2.564705	8.173423	13.144147
H	3.947876	11.302490	13.804355
H	3.078338	9.927940	14.562392
H	6.263196	9.885090	8.639488
C	7.853268	8.712979	9.551150
C	7.564536	8.379151	11.916080
C	2.950767	6.389460	10.917130
C	2.716368	6.994537	8.603426
H	3.128606	9.087841	8.208373
C	0.875367	11.115314	13.522166
C	-0.040756	11.843407	11.420144
H	1.373915	11.725428	9.771088
C	3.829302	12.453439	6.707436
H	8.396878	8.568782	8.616814
C	8.308375	8.163794	10.751563
H	7.878889	7.958784	12.872177
H	2.921550	5.660914	11.728949
C	2.663556	6.018674	9.603768
H	2.501378	6.753726	7.561731
H	0.783900	10.908627	14.589226
C	-0.207043	11.609079	12.786300
H	-0.856155	12.224624	10.804418
H	4.793499	12.870474	6.379863
H	3.078730	13.258525	6.713090
H	3.514955	11.682923	5.986547
H	9.223736	7.570439	10.782539
H	2.405927	4.985695	9.363805
H	-1.164919	11.800328	13.273005

[Ru(TPA)(MeCN)(NH_2)]$^{2+}$, S = 1/2, gas phase

Ru	4.136231	10.482679	10.819600
N	4.184397	9.517389	12.677955
N	6.036468	9.638947	10.662680
N	3.300195	8.621986	10.218249
N	2.261292	11.081545	11.514686
N	4.007917	11.298799	8.959836
N	4.923060	12.118490	11.502085
C	5.631314	9.493885	13.059644
C	3.619457	8.125542	12.590907
C	3.350667	10.379581	13.574069
C	6.770940	9.442784	9.554561

C	6.487120	9.168379	11.856525
C	3.182229	7.715182	11.207275
C	2.913111	8.288770	8.970705
C	2.109959	10.843262	12.845209
C	1.221355	11.551161	10.804890
C	3.951887	11.820005	7.927120
H	5.919223	12.341502	11.448236
H	4.388680	12.951946	11.756584
H	5.814212	8.791542	13.887620
H	5.887873	10.503076	13.413318
H	4.371066	7.411525	12.959837
H	2.760761	8.048110	13.274824
H	3.958170	11.259293	13.831529
H	3.098559	9.857547	14.510195
H	6.355799	9.834817	8.624561
C	7.995192	8.778186	9.584405
C	7.694844	8.482239	11.954461
C	2.667299	6.438135	10.973736
C	2.391913	7.037895	8.663005
H	3.031542	9.056535	8.205859
C	0.895626	11.070389	13.487624
C	-0.016601	11.814054	11.387805
H	1.397718	11.717214	9.741013
C	3.880936	12.487051	6.637431
H	8.564031	8.646994	8.662343
C	8.465735	8.288649	10.803680
H	8.032939	8.108443	12.923054
H	2.583505	5.724824	11.796669
C	2.265508	6.091811	9.684683
H	2.091515	6.811137	7.638685
H	0.796439	10.865083	14.555408
C	-0.184090	11.569098	12.751417
H	-0.833421	12.201007	10.776099
H	3.515878	13.517953	6.770813
H	3.192043	11.947643	5.968416
H	4.878724	12.520299	6.172313
H	9.419069	7.758611	10.860918
H	1.860139	5.098635	9.478906
H	-1.143355	11.758937	13.238055

$[Ru(TPA)(MeCN)(NH_2)]^{2+}$, S = 1/2, solution

Ru	4.215533	10.469255	10.820462
N	4.272148	9.462723	12.649505
N	6.145395	9.703397	10.705359
N	3.277220	8.653815	10.208547

N	2.318820	11.009108	11.466999
N	4.107111	11.267833	8.963628
N	4.959157	12.095680	11.558613
C	5.695438	9.555911	13.097967
C	3.898187	8.022258	12.464762
C	3.313821	10.188250	13.536280
C	6.908931	9.541365	9.612310
C	6.607687	9.301615	11.919212
C	3.110291	7.767139	11.211978
C	2.680642	8.430713	9.023451
C	2.106100	10.663023	12.762789
C	1.308922	11.505097	10.732722
C	4.041589	11.704830	7.894533
H	5.949460	12.347039	11.534668
H	4.397193	12.853833	11.950464
H	5.899719	8.854756	13.921196
H	5.859314	10.578733	13.464515
H	4.829726	7.440990	12.387266
H	3.368157	7.654334	13.354543
H	3.839879	11.072315	13.925517
H	3.018087	9.566456	14.393790
H	6.478851	9.876901	8.666910
C	8.180700	8.976796	9.679714
C	7.864786	8.717728	12.052798
C	2.317791	6.629493	11.059988
C	1.892444	7.307514	8.792490
H	2.838261	9.180362	8.247972
C	0.849939	10.804167	13.348453
C	0.033787	11.687127	11.262400
H	1.539460	11.753044	9.695293
C	3.944908	12.250228	6.554073
H	8.771154	8.866785	8.768713
C	8.666892	8.558480	10.919423
H	8.205246	8.391952	13.037259
H	2.195009	5.941607	11.898511
C	1.700226	6.393625	9.831516
H	1.430678	7.164648	7.814246
H	0.703614	10.506121	14.388147
C	-0.200612	11.328676	12.591057
H	-0.758789	12.096304	10.633848
H	4.333467	13.280234	6.547595
H	2.892034	12.256905	6.232455
H	4.536661	11.635104	5.859030
H	9.656965	8.105398	11.004733
H	1.074953	5.509493	9.688281
H	-1.191882	11.449406	13.033521

[Ru(TPA)(MeCN)$_2$]$^{2+}$ (XI^{2+}), *S* = 0, gas phase

Ru	2.332781	-0.935979	15.553615
N	2.361875	-0.863169	17.568270
N	4.289775	-1.604708	15.249324
N	2.309806	-1.100559	13.460811
N	0.364834	-0.317358	15.248686
N	1.556017	-2.850529	15.433439
N	2.992966	0.978401	15.569483
C	2.419522	-0.772303	18.722874
C	4.686541	-1.460949	13.956416
C	5.117205	-2.186221	16.132714
C	3.701851	-0.766076	13.039342
C	1.282892	-0.122134	13.001643
C	1.974678	-2.507355	13.064217
C	0.091105	-0.112193	13.933440
C	-0.631428	-0.258044	16.147331
C	1.352130	-3.307387	14.177181
C	1.102913	-3.575671	16.475558
C	3.355770	2.076008	15.645393
C	2.491264	-0.657293	20.172153
C	5.933516	-1.907797	13.527768
C	6.384916	-2.642859	15.774464
H	4.742445	-2.283099	17.153016
H	3.882431	-1.030968	11.984700
H	3.822258	0.322452	13.134922
H	0.974739	-0.316500	11.961884
H	1.750518	0.873974	13.026143
H	2.911255	-3.011167	12.779059
H	1.334956	-2.509285	12.168539
C	-1.204705	0.149338	13.495199
H	-0.353644	-0.438485	17.187204
C	-1.947708	0.016355	15.780261
C	0.671472	-4.499787	13.932956
H	1.287226	-3.160874	17.466331
C	0.431414	-4.781438	16.307226
C	3.811305	3.453314	15.754018
H	2.286100	-1.631318	20.643227
H	1.750712	0.073473	20.533638
H	3.496008	-0.322264	20.475149
H	6.224987	-1.791253	12.481971
C	6.801614	-2.501642	14.450687
H	7.027402	-3.105327	16.525663
C	-2.241284	0.220233	14.431449
H	-1.400830	0.300113	12.431697
H	-2.726114	0.061612	16.543937
C	0.204380	-5.251992	15.011421

H	0.516716	-4.837239	12.906046
H	0.087235	-5.336690	17.181247
H	2.968045	4.111189	16.016756
H	4.242092	3.791461	14.798706
H	4.582263	3.533092	16.536579
H	7.785528	-2.855048	14.134692
H	-3.263801	0.427867	14.108232
H	-0.328450	-6.190694	14.843945

$[Ru(TPA)(MeCN)_2]^{2+}$ (XI^{2+}), *S* = 0, solution

Ru	2.337364	-0.926810	15.562429
N	2.364818	-0.870551	17.571726
N	4.287352	-1.585124	15.255973
N	2.305796	-1.082119	13.473559
N	0.373680	-0.311896	15.273383
N	1.567007	-2.838141	15.443770
N	2.998343	0.979828	15.561969
C	2.393764	-0.823421	18.728849
C	4.681539	-1.435434	13.963893
C	5.113949	-2.163091	16.142638
C	3.694995	-0.750291	13.043746
C	1.280117	-0.099450	13.022355
C	1.961132	-2.483853	13.070274
C	0.096511	-0.093800	13.961588
C	-0.616082	-0.269949	16.180430
C	1.359197	-3.290088	14.186872
C	1.122034	-3.563645	16.488945
C	3.376386	2.073510	15.588402
C	2.428125	-0.771856	20.179933
C	5.932058	-1.874949	13.536963
C	6.383797	-2.611460	15.784027
H	4.736274	-2.262599	17.161920
H	3.870685	-1.031876	11.993808
H	3.819878	0.338663	13.125996
H	0.964107	-0.295254	11.986443
H	1.746426	0.896617	13.048331
H	2.888033	-2.989505	12.759842
H	1.301365	-2.475214	12.190595
C	-1.203198	0.165804	13.533139
H	-0.331913	-0.465917	17.215890
C	-1.934444	0.001839	15.821122
C	0.678989	-4.483767	13.944034
H	1.309404	-3.150572	17.479737
C	0.451292	-4.769523	16.319922
C	3.854350	3.444256	15.611475

H	2.143536	-1.752057	20.592882
H	1.722993	-0.008597	20.543533
H	3.443322	-0.517767	20.521815
H	6.215961	-1.755253	12.489796
C	6.800603	-2.464858	14.460189
H	7.026272	-3.071523	16.536740
C	-2.234000	0.220453	14.475371
H	-1.398875	0.321434	12.470677
H	-2.708509	0.031031	16.589890
C	0.219022	-5.237152	15.023596
H	0.520154	-4.812017	12.915085
H	0.111035	-5.323655	17.196141
H	3.039668	4.128180	15.327610
H	4.687276	3.559669	14.900839
H	4.204522	3.700468	16.623219
H	7.786561	-2.813040	14.144446
H	-3.259319	0.424061	14.158761
H	-0.315862	-6.174508	14.856052

$[Ru(TPA)(MeCN)_2]^{3+}$, S = 1/2, gas phase

Ru	2.298056	-1.000242	15.514917
N	2.334335	-0.913155	17.577521
N	4.258135	-1.612666	15.229737
N	2.274132	-1.135441	13.434659
N	0.362759	-0.308365	15.237131
N	1.553587	-2.891491	15.401811
N	2.960436	0.960535	15.552158
C	2.396353	-0.793486	18.727937
C	4.655866	-1.486602	13.927886
C	5.094728	-2.149490	16.142272
C	3.671998	-0.810306	13.000088
C	1.255333	-0.139058	12.969416
C	1.921454	-2.541803	13.018138
C	0.081977	-0.083565	13.920435
C	-0.608400	-0.187952	16.165830
C	1.361566	-3.365658	14.142728
C	1.150058	-3.627389	16.466846
C	3.324376	2.054204	15.666261
C	2.470518	-0.645187	20.170524
C	5.915227	-1.912656	13.522558
C	6.369247	-2.587393	15.798353
H	4.717607	-2.227761	17.162879
H	3.839621	-1.096603	11.950427
H	3.797528	0.280323	13.067839
H	0.931635	-0.354133	11.940028

H	1.742217	0.848425	12.957071
H	2.838846	-3.035458	12.660556
H	1.228954	-2.517732	12.163696
C	-1.203211	0.256605	13.513394
H	-0.321289	-0.384692	17.199706
C	-1.909715	0.161829	15.822327
C	0.743665	-4.593355	13.922554
H	1.324829	-3.195135	17.450907
C	0.542071	-4.864014	16.312696
C	3.780453	3.422597	15.806821
H	2.178356	-1.587152	20.662839
H	1.791512	0.157687	20.500905
H	3.500047	-0.389148	20.469774
H	6.219027	-1.811504	12.478235
C	6.787512	-2.467248	14.469439
H	7.020694	-3.018407	16.561226
C	-2.213872	0.384008	14.475550
H	-1.416553	0.428128	12.456033
H	-2.672006	0.252993	16.598702
C	0.329004	-5.355883	15.018527
H	0.594956	-4.955332	12.902776
H	0.235054	-5.431804	17.193199
H	2.942370	4.115450	15.621302
H	4.584170	3.629149	15.080714
H	4.164004	3.587165	16.827123
H	7.782443	-2.805672	14.168082
H	-3.229327	0.653203	14.173129
H	-0.154853	-6.324135	14.864961

$[Ru(TPA)(MeCN)_2]^{3+}$, S = 1/2, solution

Ru	2.290406	-1.005604	15.520378
N	2.325664	-0.925243	17.560043
N	4.246452	-1.598553	15.244050
N	2.269431	-1.127900	13.443929
N	0.369258	-0.298247	15.245826
N	1.553748	-2.892396	15.399788
N	2.946264	0.929875	15.564875
C	2.391254	-0.816545	18.708000
C	4.645645	-1.474522	13.947108
C	5.071790	-2.124075	16.169221
C	3.667021	-0.810485	13.010526
C	1.264743	-0.118291	12.984109
C	1.897186	-2.523264	13.016170
C	0.094964	-0.061396	13.933523
C	-0.593586	-0.185998	16.180097

C	1.366306	-3.359466	14.140957
C	1.164356	-3.628217	16.466877
C	3.313521	2.022032	15.642359
C	2.470289	-0.686876	20.147954
C	5.908995	-1.898250	13.551999
C	6.348833	-2.557610	15.833550
H	4.684487	-2.197971	17.186215
H	3.835763	-1.116088	11.967887
H	3.800122	0.279766	13.068036
H	0.939639	-0.327653	11.955503
H	1.757346	0.865412	12.986234
H	2.796962	-3.019425	12.621654
H	1.175060	-2.478126	12.188974
C	-1.190264	0.281598	13.529789
H	-0.303680	-0.395577	17.210666
C	-1.893621	0.166121	15.837462
C	0.759145	-4.592178	13.919277
H	1.338281	-3.194171	17.450332
C	0.566208	-4.868080	16.308942
C	3.777013	3.387775	15.733039
H	2.154748	-1.631771	20.616867
H	1.807598	0.127265	20.479597
H	3.507195	-0.459474	20.439114
H	6.207103	-1.801463	12.506805
C	6.772864	-2.442836	14.507231
H	6.993972	-2.981091	16.604585
C	-2.196443	0.398603	14.493450
H	-1.394896	0.454731	12.472130
H	-2.652869	0.248161	16.616348
C	0.355011	-5.356088	15.014371
H	0.612449	-4.943293	12.896502
H	0.265324	-5.436737	17.189704
H	2.956271	4.067314	15.454053
H	4.622731	3.531554	15.042548
H	4.097608	3.599378	16.764707
H	7.769428	-2.780229	14.213784
H	-3.211914	0.666462	14.193172
H	-0.123691	-6.325629	14.860301

Transition State {[Ru(TPA)(NH_2)(NH_3)]·····[Ru(TPA)(NH_2)(NH_3)]}$^{4+}$, *S* = 0, solution

Ru	-3.18818	11.7017	6.259959
N	-1.23877	10.88394	6.031141
N	-2.02344	13.36503	6.781331
N	-2.9547	12.05139	4.22051
N	-3.70588	9.803322	5.59723

N	-4.92402	12.68125	6.472121
N	-3.30559	11.30495	8.381374
C	-0.39898	11.56425	7.050657
C	-0.73509	13.03739	7.065032
C	-1.40533	9.422999	6.270264
C	-2.66828	8.930411	5.61033
C	-0.72449	11.17078	4.658143
C	-2.38357	14.66039	6.775753
C	-1.80227	11.57998	3.694529
C	-3.89783	12.52677	3.387866
C	-4.85516	9.449542	4.999532
C	0.214358	14.01252	7.358631
C	-2.78829	7.657148	5.058925
C	-1.48767	15.6842	7.071645
C	-1.58528	11.54506	2.316844
C	-3.7462	12.5401	2.006695
C	-5.04543	8.195553	4.429818
C	-0.16566	15.35612	7.372171
C	-3.9973	7.276114	4.47153
C	-2.56898	12.02935	1.456789
H	-2.673	11.9103	8.912885
H	-4.2308	11.50749	8.76951
H	-3.09035	10.35167	8.686278
H	-5.30879	13.18231	5.66998
H	-4.92752	13.35696	7.240312
H	0.674535	11.40878	6.862114
H	-0.62284	11.12223	8.032071
H	-1.48212	9.266123	7.355886
H	-0.52879	8.858149	5.918067
H	1.242753	13.71315	7.567802
H	0.564725	16.13472	7.602384
H	-1.83066	16.71987	7.05783
H	-3.42207	14.8774	6.522018
H	-0.01075	12.00614	4.725444
H	-0.16071	10.30894	4.27222
H	-0.64674	11.14097	1.933221
H	-2.41912	12.0086	0.375301
H	-4.54813	12.93548	1.38179
H	-4.80836	12.90712	3.843876
H	-1.9343	6.978444	5.086697
H	-4.1104	6.279669	4.039582
H	-6.00493	7.954659	3.97013
H	-5.64298	10.19577	5.008466
H	-10.1548	8.178111	4.52073
H	-9.48757	8.935097	2.225922
H	-11.6634	9.197922	5.860625

H	-9.76203	7.132401	6.99472
C	-9.2642	8.6508	4.961209
H	-8.49772	7.867341	5.065173
C	-8.88362	9.717365	2.68861
H	-12.9434	11.19793	5.26205
H	-7.87369	5.841048	7.98954
C	-10.9191	9.775648	6.429579
C	-9.30999	8.085801	7.311483
N	-9.56803	9.167963	6.33085
C	-8.70439	9.715308	4.070562
H	-8.41776	10.74483	0.841263
C	-7.24826	6.732717	7.920032
C	-12.0584	11.81048	5.442523
C	-8.29344	10.72532	1.925897
H	-11.2239	9.740055	7.486945
H	-9.77609	8.362829	8.268078
C	-7.82154	7.939111	7.526543
C	-10.9206	11.22284	5.99178
H	-5.42135	5.74982	8.541092
C	-5.88694	6.687872	8.231624
C	-12.0465	13.17133	5.132214
H	-12.9294	13.64789	4.700983
N	-7.97037	10.66241	4.69652
C	-7.56303	11.71906	2.580196
N	-7.0751	9.066732	7.391321
N	-9.79009	11.94325	6.215101
Ru	-8.18069	10.72541	6.76765
C	-5.14213	7.863211	8.150776
C	-7.43824	11.65646	3.962898
C	-10.8857	13.90562	5.374841
C	-5.77686	9.025948	7.72452
H	-9.7168	10.72899	9.038124
H	-7.10088	12.54367	2.035632
C	-9.77904	13.25133	5.908843
N	-8.72377	10.88705	8.847287
H	-8.21354	10.24673	9.462821
H	-4.07995	7.88963	8.39856
H	-10.8235	14.97059	5.145961
H	-6.90695	12.42506	4.518433
H	-5.23968	9.962606	7.627709
H	-8.84931	13.79228	6.089882
N	-6.85408	12.19277	7.157177
H	-8.53336	11.82626	9.206638
H	-6.5111	12.22986	8.119967
H	-7.10901	13.15398	6.920311
